# Activation of Dormant Secondary Metabolite Production by Introducing Neomycin Resistance into the Deep-Sea Fungus, *Aspergillus versicolor* ZBY-3

**DOI:** 10.3390/md12084326

**Published:** 2014-07-29

**Authors:** Yuan Dong, Cheng-Bin Cui, Chang-Wei Li, Wei Hua, Chang-Jing Wu, Tian-Jiao Zhu, Qian-Qun Gu

**Affiliations:** 1 Beijing Institute of Pharmacology and Toxicology, Beijing 100850, China; E-Mails: cndongy@163.com (Y.D.); sdrlcw@126.com (C.-W.L.); huawei0917@outlook.com (W.H.); wucj2009@163.com (C.-J.W.); 2 Key Laboratory of Marine Drugs, Chinese Ministry of Education, Institute of Marine Drugs and Food, School of Medicine and Pharmacy, Ocean University of China, Qingdao 266003, China; E-Mails: zhutj@ouc.edu.cn (T.-J.Z.); guqianq@ouc.edu.cn (Q.-Q.G.)

**Keywords:** *Aspergillus versicolor* ZBY-3, deep-sea fungus, neomycin resistance, ultrasound, antitumor activity, secondary metabolite production

## Abstract

A new ultrasound-mediated approach has been developed to introduce neomycin-resistance to activate silent pathways for secondary metabolite production in a bio-inactive, deep-sea fungus, *Aspergillus versicolor* ZBY-3. Upon treatment of the ZBY-3 spores with a high concentration of neomycin by proper ultrasound irradiation, a total of 30 mutants were obtained by single colony isolation. The acquired resistance of the mutants to neomycin was confirmed by a resistance test. In contrast to the ZBY-3 strain, the EtOAc extracts of 22 of the 30 mutants inhibited the human cancer K562 cells, indicating that these mutants acquired a capability to produce antitumor metabolites. HPLC-photodiode array detector (PDAD)-UV and HPLC-electron spray ionization (ESI)-MS analyses of the EtOAc extracts of seven bioactive mutants and the ZBY-3 strain indicated that diverse secondary metabolites have been newly produced in the mutant extracts in contrast to the ZBY-3 extract. The followed isolation and characterization demonstrated that six metabolites, cyclo(d-Pro-d-Phe) (**1**), cyclo(d-Tyr-d-Pro) (**2**), phenethyl 5-oxo-l-prolinate (**3**), cyclo(l-Ile-l-Pro) (**4**), cyclo(l-Leu-l-Pro) (**5**) and 3β,5α,9α-trihydroxy-(22*E*,24*R*)-ergosta-7,22-dien-6-one (**6**), were newly produced by the mutant u2n2h3-3 compared to the parent ZBY-3 strain. Compound **3** was a new compound; **2** was isolated from a natural source for the first time, and all of these compounds were also not yet found in the metabolites of other *A. versicolor* strains. Compounds **1**–**6** inhibited the K562 cells, with inhibition rates of 54.6% (**1**), 72.9% (**2**), 23.5% (**3**), 29.6% (**4**), 30.9% (**5**) and 51.1% (**6**) at 100 μg/mL, and inhibited also other human cancer HL-60, BGC-823 and HeLa cells, to some extent. The present study demonstrated the effectiveness of the ultrasound-mediated approach to activate silent metabolite production in fungi by introducing acquired resistance to aminoglycosides and its potential for discovering new compounds from silent fungal metabolic pathways. This approach could be applied to elicit the metabolic potentials of other fungal isolates to discover new compounds from cryptic secondary metabolites.

## 1. Introduction

Marine microbial natural products, especially those derived from fungi, have come to be rich and promising sources of novel compounds with biological and pharmaceutical properties, providing a number of drug leads and other healthcare ingredients [1,2,3,4,5,6]. Although a great number of novel and bioactive compounds have been increasingly discovered from marine microbial sources [1,2,3,4,5,6,7,8,9,10,11], the great biosynthetic potential of microbial isolates, including the fungi, has so far not been fully elicited, because their major biosynthetic pathways that produce secondary metabolites are silent in laboratory culture conditions [12,13,14,15,16]. Various strategies have been developed to activate silent pathways to elicit the metabolic potentials of microbial isolates [12,13,14,15,16,17,18,19,20,21,22,23,24,25,26]. One strain-many compounds (OSMAC) [17], co-cultivation [18] and chemical epigenetics [19,20] strategies have been widely applied by microbial chemists to access cryptic secondary metabolites [13,14,15,16,17,18,19,20,21], because the culture-based, simple procedures outlined by these strategies are suitable for microbial chemists. Ribosome engineering [22,23] has been also able to activate silent pathways by introducing drug-resistance mutations in bacteria to discover new antibacterial agents [24]. This strategy has been recently extended to fungi [27]. The similar mutation-based mutagenesis strategy that was developed just recently [28] may also suit microbial chemists for the discovery of novel bioactive compounds from silent pathways of fungal secondary metabolites [28,29,30]. In spite of the strategies mentioned, additional simple approaches that are suitable for use by microbial chemists are still needed for research work on secondary metabolites from silent pathways.

Ultrasound has been widely applied in various fields, for instance, clinical use both for diagnosis and therapy [31,32,33,34], applications in industrial food technology for processing, preservation and extraction [35,36,37], use in fermentation biotechnology to enhance microbial productivity [38,39], application in chemistry and chemical engineering [40,41], and so on. Ultrasound has been also used to investigate the effects on biological systems [42,43,44] and both drug [43,44] and gene [43,44,45,46,47,48,49] delivery. The transient membrane permeability induced by ultrasonic exposure was known to enhance the entry of exogenous substances into cells and cellular organelles [42,43,44,45,46,47,48,49]. On the other hand, neomycin, an aminoglycoside antibiotic that attacks the ribosome, has been used in ribosome engineering to modulate ribosomal function to alter secondary metabolisms by introducing drug-resistance mutations in bacteria [22], but not yet in fungi. Another aminoglycoside antibiotic attacking the ribosome, gentamicin, has also been used in bacteria but not in fungi in ribosome engineering [22], until a dimethyl sulfoxide (DMSO)-mediated new approach was developed to introduce gentamicin-resistance into a fungus [27]. Generally, aminoglycoside antibiotics that attack the ribosome are antibacterial, but not antifungal. The insensitivity of fungi to the aminoglycoside antibiotics restricted the application of aminoglycoside antibiotics to fungi in ribosome engineering. As the introduction of drug-resistance to aminoglycoside antibiotics has been known to activate silent biosynthetic pathways of bacterial secondary metabolites [22,23,24], it is worthwhile to investigate a proper method to introduce acquired resistance to the antibiotics in fungi to further investigate fungal secondary metabolites from silent pathways.

**Figure 1:**
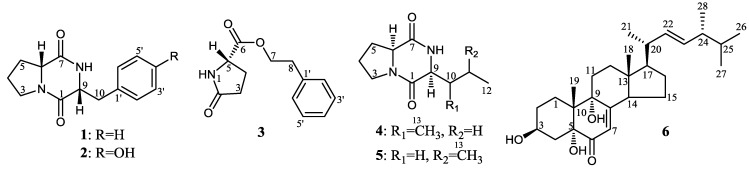
Structures of **1**–**6** newly produced by neomycin-resistant mutant u2n2h3-3.

During ongoing research for developing new approaches to activate the silent pathways of secondary metabolites in fungi [28,29,30], we tested neomycin to introduce drug-resistance into a deep-sea fungus, *Aspergillus versicolor* ZBY-3, to awaken dormant metabolite production. Previously, we have reported that the acquired resistance of a marine-derived *Penicillium purpurogenum* G59 to gentamicin could be successfully introduced by combined use of DMSO, well known as a membrane permeabilizer, relying on its effect on the penetration of gentamicin into cells [27]. This has resulted in the activated production of antitumor secondary metabolites originally silent in the G59 strain [27]. In the present study, in view of the documented effect of ultrasound on membrane permeability [42,43,44,45,46,47,48,49], we used ultrasound as a mediator to introduce neomycin resistance into *A.* *versicolor* ZBY-3, which was inactive in the production of antitumor metabolites. The acquired resistance to neomycin, as testified by the resistance test, was introduced into the ZBY-3 strain by treatment of the ZBY-3 spores with a high concentration of neomycin combined with proper ultrasound irradiation. Activated production of the antitumor metabolites originally silent in the ZBY-3 strain by the introduction of the drug-resistance in mutants was evidenced both by bioassays and HPLC-photodiode array detector (PDAD)-UV and HPLC-electron spray ionization (ESI)-MS analyses. The followed isolation and characterization further demonstrated that six antitumor metabolites, **1**–**6** (Figure 1), were newly produced by a neomycin-resistant mutant, u2n2h3-3, which also resulted in the discovery of the new compound **3**.

We report herein recent results of our research work on the activation of silent antitumor metabolites in *A.* *versicolor* ZBY-3 by ultrasound-mediated introduction of neomycin resistance.

## 2. Results and Discussion

### 2.1. Mutant Selection and Resistance Test

According to our previous experience in the treatment of *P. purpurogenum* G59 spores by gentamicin in aqueous DMSO at 4 °C to introduce drug resistance [27], and further, in view of the documented effect of ultrasound on the membrane permeability [42,43,44,45,46,47,48,49], we tested the introduction of drug resistance in *A.* *versicolor* ZBY-3 by neomycin treatment of ZBY-3 spores at 4 °C for different times after ultrasound irradiation of the spores in the presence of neomycin at different concentrations. In preliminary tests searching for suitable conditions, the treatment of ZBY-3 spores with 200–800 mg/mL neomycin at 4 °C did not inhibit strain growth on potato dextrose agar (PDA) plates at 28 °C. The strain grew as well as the ZBY-3 strain without the spores having been treated by neomycin, as described below in the resistance test, indicating the insensitivity of the ZBY-3 strain to neomycin. Similarly, treatment of the ZBY-3 spores only with ultrasound irradiation by 2 s of irradiation every 4 s with an 8-min duration cycle at 200 or 400 W of output power also did not inhibit the strain growth on PDA plates at 28 °C. In contrast, ultrasound irradiation of the spores by 2 s of irradiation every 4 s with an 8-min duration cycle at 800 W of output power resulted in the complete inhibition of the strain’s growth, in spite of the presence or absence of neomycin. Fortunately, ultrasound irradiation for 2 s every 4 s with an 8-min duration cycle at 200 or 400 W of output power in the presence of 200 or 400 mg/mL neomycin properly inhibited the strain’s growth, which made its use suitable for selecting drug-resistant mutants from PDA plates by single colony isolation.

Thus, in the present study, ultrasound irradiation of the ZBY-3 spores was run by the 2-s irradiation every 4 s with an 8-min duration cycle at 200 or 400 W of output power. Fresh spore suspensions with the same spore density and 200 or 400 mg/mL neomycin in water were subjected to ultrasound irradiation. After ultrasound irradiation, the irradiated spore suspension was kept at 4 °C for up to 72 h for further treatment of the spores with the neomycin contained therein. During the treatment period, each 100 µL portion of the treated spore suspensions was spread on PDA plates at 3, 12, 24 and 48 h of the treatment and incubated at 28 °C for 3–5 days. Mutant colonies developed on the PDA plates were selected by single colony isolation during the incubation period with different appearances. A total of 30 mutants were selected, including 12 from the 200-W ultrasound irradiation and 18 from the 400-W ultrasound irradiation groups. Among them, 15 of the 30 mutants were from the 200 and 15 from the 400 mg/mL neomycin treatment groups, respectively (Table 1).

All of the above-mentioned 30 mutants and their parent ZBY-3 strain were serially passaged four times by transfers under nonselective conditions onto PDA plates, followed by a further four times of passage over two years, to obtain the first to eighth passages of the mutants and the parent strain. Of them, the fourth, sixth and eighth passages were used in the first, second and third rounds of fermentations for the MTT assay to evaluate the effects of their culture extracts on K562 cells, respectively, and the extracts from the third round of fermentation of seven bioactive mutants and the parent ZBY-3 strain were also used in the HPLC-PDAD-UV and HPLC-ESI-MS analyses. The eighth passage of a bioactive mutant, u2n2h3-3, and the ZBY-3 strain was used in the resistance test and also in the large-scale fermentation for the investigation of newly produced metabolites by the mutant, u2n2h3-3. All of the 30 mutants and parent ZBY-3 strain maintained their genetic stability well over two years during the eight passages, as indicated by the effects of their culture extracts on the K562 cells, as shown in Section 2.2.

**Table 1:** Mutant numbers selected by ultrasound-mediated neomycin treatment of ZBY-3 spores ^a^.

Ultrasound (W)	Neomycin (mg/mL)	Treatment Time at 4 °C				Total
		3 h	12 h	24 h	48 h	
200	200	4	1	1	2	8
	400	1	2	1	0	4
	Sum	5	3	2	2	12
400	200	1	0	2	4	7
	400	2	1	3	5	11
	Sum	3	1	5	9	18
Total		8	4	7	11	30

^a^ After ultrasound irradiation of the fresh ZBY-3 spore suspensions at the given ultrasound power and neomycin concentration, the spore suspensions were kept at 4 °C to treat the spores with neomycin. In the treatment period, each 100 μL of the spore suspensions was spread on PDA plates at the given treatment time and incubated at 28 °C for 3–5 days. Mutant colonies developed on the PDA plates were selected by single colony isolation during the incubation period to obtain mutants showing the numbers in this table.

**Figure 2:**
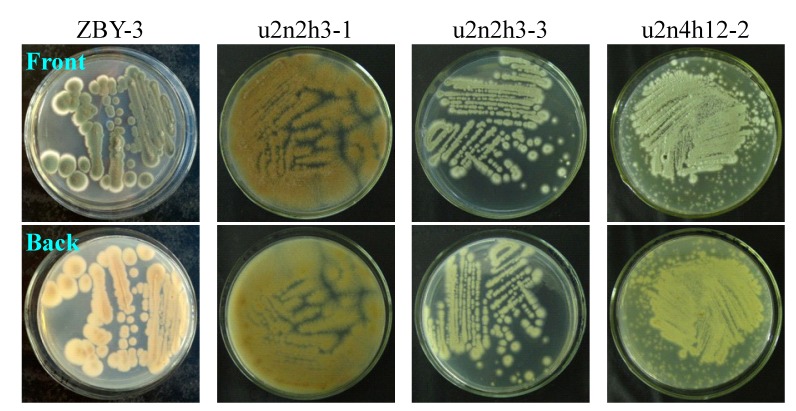
Phenotypes of the ZBY-3 strain and selected mutants growing on PDA plates by incubation at 28 °C for three days.

The strain growth and spore formation of the 30 mutants and the parent ZBY-3 strain were not so obviously different, but their major difference mainly appeared in the morphology, the color of mycelia or pigment formation, when grown on PDA plates at 28 °C. The phenotypic differences of the parent ZBY-3 strain and the mutants are shown by photographs of some selected mutants and the ZBY-3 strain growing on PDA plates as typical examples in Figure 2. The phenotypes of these mutants and parent ZBY-3 strain were not fully identical with the appearances of their corresponding colonies observed during mutant selection on the PDA plates on which the spore suspensions were spread soon after neomycin treatment. The stress from the ultrasound-mediated neomycin treatment likely affected the phenotypic development of these colonies. However, once the colonies were picked up, streaked out on PDA plates and then incubated at 28 °C, their own phenotypes appeared, as shown by typical examples in Figure 2. Corresponding to the phenotypic differences on the PDA plates, as shown in Figure 2, similar differences in the colors of mycelia and culture broths were also observed by fermentation in liquid medium.

**Figure 3:**
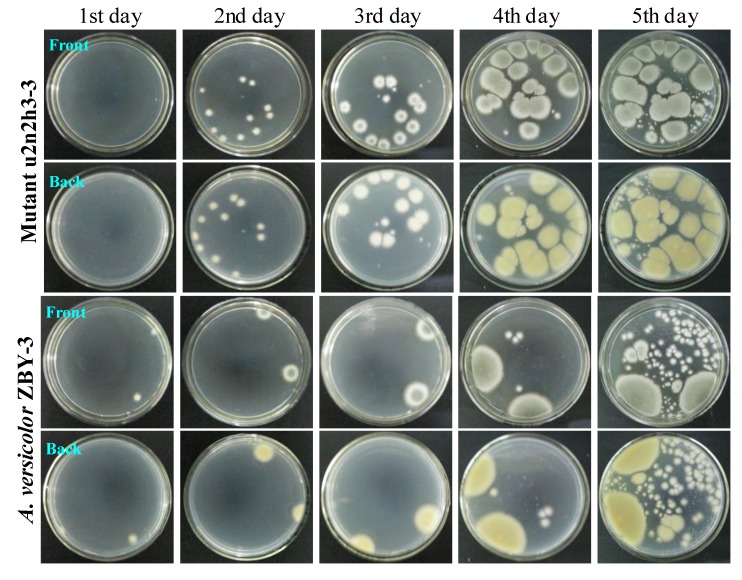
The growth of *A.* *versicolor* ZBY-3 and its mutant, u2n2h3-3, on PDA plates by incubation at 28 °C for different times (day) after treatment of their spores with neomycin. The fresh ZBY-3 and u2n2h3-3 spore suspensions were treated with 200 mg/mL neomycin, as done for selecting the mutant, and each 100 μL portion of the treated spore suspensions was spread on PDA plates, incubated at 28 °C and photographed at the given incubation times (day).

The acquired resistance of the mutants to neomycin was testified by a resistance test using a mutant, u2n2h3-3, and the parent strain, ZBY-3. After ultrasound irradiation of the fresh u2n2h3-3 and ZBY-3 spore suspensions by 2-s irradiation every 4 s with an 8-min duration cycle at 200 W of ultrasound power in the presence of 200 mg/mL of neomycin, the spore suspensions were kept at 4 °C for 3 h for further treatment of the spores with neomycin, as done for selecting the mutant, u2n2h3-3. Each 100 μL of the spore suspensions was then spread on PDA plates and incubated at 28 °C for five days to examine their day-by-day growth. Many colonies of the mutant, u2n2h3-3, appeared on the second day of incubation and quickly grew up to become confluent, indicating the acquired resistance of the mutant to neomycin treatment in comparison to the mutants’ growth with the parent ZBY-3 strain (Figure 3). The mutant grew far better than the control ZBY-3 strain in the resistance test, as seen in Figure 3. In contrast to the mutant growth, although two single colonies of the ZBY-3 strain appeared early on the first day of incubation and grew well with incubations, a few other colonies appeared as late as on the fourth day of incubation and, further, more colonies later on the fifth day of incubation (Figure 3). These colonies, especially those developed at earlier, before the fourth day of incubation, should be mutant colonies resistant to the neomycin treatment, which just reproduced a similar phenomenon allowing single colony isolation in the mutant selection. The many colonies coming into appearance later on the fifth day of incubation, both for the mutant and parent strains, in Figure 3, seem likely to be formed from fresh spores of the early developing colonies on the plates.

Incidentally, when 100 μL of the fresh ZBY-3 spore suspension were spread on the PDA plate without treatment, as in the resistance test, and incubated at 28 °C, the stain grew very well. A lot of fine colony species appeared throughout the surface of the PDA plate on the second day of incubation, quickly growing into a confluent lawn on the third day of incubation, and the color of colonies or the confluent lawn differed from those shown in Figure 3 for the mutant and the control parent strain in the resistance test.

Thus, the resistance test results demonstrated that the treatment of ZBY-3 spores with neomycin by ultrasound radiation has successfully introduced the drug resistance into the mutant, u2n2h3-3.

### 2.2. Estimation of the Activated Antitumor Metabolite Production in the Mutants by Bioassays

In order to preliminarily estimate the activated production of silent bioactive metabolites in the mutants, we tested the antitumor activities of the EtAOc extracts of the ZBY-3 strain and the 30 mutant cultures. The mutants and the control ZBY-3 strain were fermented at the same time and with the same conditions to obtain their ethyl acetate (EtOAc) extracts. Then, the extracts were subjected to the MTT assay using K562 cells to evaluate their antitumor activities. Extracts of 22 mutants, 73.3% of the 30 mutants, inhibited the K562 cells with inhibition rate (IR%) values larger than 20% at 100 µg/mL (Table 2). Among them, extracts of 20 mutants possessing 66.6% of the 30 mutants inhibited K562 cells by IR% over 30% at 100 µg/mL, and 16 of them, accounting for 53.3% of the 30 mutants, showed more significant inhibition of the K562 cells (IR% > 40% at 100 µg/mL). In contrast, the ZBY-3 extract did not inhibit the K562 cells (an IR% of 1.8% at 100 µg/mL). These assay data indicated that these mutants have acquired the metabolic capability to produce antitumor metabolites by the introduction of neomycin resistance.

**Table 2:** Results of the MTT assay on K562 cells for the ZBY-3 and 30 mutant samples at 100 μg/mL ^a^.

Strain ^b^	Condition for Selecting Mutant Strain ^c^			IR% ^d^ (Mean ± SD, *n* = 3)
	Ultrasound (W)	Neomycin (mg/mL)	Treated Time at 4 °C (h)	
ZBY-3	-	-	-	1.8 ± 2.0
u2n2h3-1	200	200	3	67.1 ± 2.3 *
u2n2h3-2	200	200	3	17.2 ± 3.3
u2n2h3-3	200	200	3	62.9 ± 2.4 *
u2n2h3-4	200	200	3	61.4 ± 5.9 *
u2n2h12-1	200	200	12	6.0 ± 3.9
u2n2h24-1	200	200	24	48.9 ± 6.9 *
u2n2h48-1	200	200	48	22.9 ± 2.2 **
u2n2h48-2	200	200	48	14.9 ± 1.2
u2n4h3-1	200	400	3	16.7 ± 2.8
u2n4h12-1	200	400	12	72.0 ± 2.0 *
u2n4h12-2	200	400	12	83.3 ± 2.6 *
u2n4h24-1	200	400	24	32.5 ± 0.6 ***
u4n2h3-1	400	200	3	54.4 ± 8.4 *
u4n2h24-1	400	200	24	9.7 ± 1.8
u4n2h24-2	400	200	24	71.1 ± 1.0 *
u4n2h48-1	400	200	48	43.1 ± 21.7 *
u4n2h48-2	400	200	48	2.4 ± 6.1
u4n2h48-3	400	200	48	30.4 ± 8.9 ***
u4n2h48-4	400	200	48	55.7 ± 5.1 *
u4n4h3-1	400	400	3	60.8 ± 6.9 *
u4n4h3-2	400	400	3	71.3 ± 8.4 *
u4n4h12-1	400	400	12	49.8 ± 3.2 *
u4n4h24-1	400	400	24	38.5 ± 3.1 ***
u4n4h24-2	400	400	24	44.6 ± 35.7 *
u4n4h24-3	400	400	24	49.6 ± 2.7 *
u4n4h48-1	400	400	48	1.1 ± 17.8
u4n4h48-2	400	400	48	12.9 ± 3.8
u4n4h48-3	400	400	48	56.6 ± 5.8 *
u4n4h48-4	400	400	48	39.8 ± 1.8 ***
u4n4h48-5	400	400	48	22.3 ± 7.1 **

^a^ Test samples are the EtOAc extracts of the parent ZBY-3 and its 30 mutant cultures; ^b^ The nomenclature of the mutants: u2 or u4 indicates the 200 or 400 W of output power of ultrasound irradiation; n2 or n4 indicates the 200 or 400 mg/mL of neomycin contained in the spore suspension. Arabic numerals between h and a hyphen indicate the treatment times (hours) of the spore suspension at 4 °C after ultrasound irradiation, Arabic numerals following a hyphen are the serial number of the mutants selected at the same treatment conditions; ^c^ The fresh ZBY-3 spore suspension with neomycin at the given concentration was subjected to ultrasound irradiation by 2 s of irradiation every 2 s with an 8-min duration cycle at the given output power, and the spores were further treated with the contained neomycin at 4 °C for the given times. Then, a 100-μL portion of the spore suspension was spread on PDA plates and incubated at 28 °C for 3–5 days to obtain the mutants by single colony isolation; ^d^ The triplicate MTT tests were carried out using samples from three rounds of individual fermentations upon the fourth, sixth and eighth passages of the 30 mutants and the parent ZBY-3 strain, respectively. The data marked with asterisks show the IR%: *, IR% > 40%; **, IR% in 30%–20%; ***, IR% in 40%–30%. Docetaxel was used as a positive control in the MTT assay, inhibiting the K562 cells with an IR% of 55.7 ± 16.9 at 100 μg/mL.

### 2.3. Chromatographic Analysis of Metabolite Production Induced by the Introduction of Drug Resistance

To examine secondary metabolites produced in the mutants by the introduction of drug resistance, the EtOAc extracts of the ZBY-3 strain and seven bioactive mutants were subjected to HPLC-PDAD-UV and HPLC-ESI-MS analyses. The control ZBY-3 and seven mutant extracts produced different HPLC profiles, and many new peaks were detected in the mutant extracts, as shown in Figures S1 and S2 in the Supplementary Information for the HPLC-PDAD-UV and HPLC-ESI-MS analyses, respectively. New peaks in the mutant extracts were verified by their UV (Figure S1) and MS (Figure S2) spectra and indicated that diverse secondary metabolites were being newly produced by these mutants. These analyses, coupled with the above bioassay results, indicated that some biosynthetic pathways originally silent in the parent ZBY-3 strain were activated in these mutants to produce bioactive secondary metabolites. Incidentally, over production of some weakly-expressed secondary metabolites in the parent ZBY-3 strain by the mutants was also detected in the HPLC-PDAD-UV and HPLC-ESI-MS analyses.

### 2.4. Antitumor Metabolites 1–6 Produced by Mutant u2n2h3-3 by Introducing Drug Resistance

#### 2.4.1. Fermentation, Isolation and Identification of Known Compounds **1**–**2** and **4**–**6**

Large-scale fermentation and extraction of the bioactive mutant, u2n2h3-3, gave an EtOAc extract that inhibited the K562 cells with an IR% value of 62.9% at 100 µg/mL. However, the control ZBY-3 extract that was obtained by fermentation of the ZBY-3 strain at the same time and at the same conditions showed no inhibitory effect on the K562 cells (an IR% of 3.8% at 100 μg*/*mL). Repeated column chromatography of the mutant extract, tracing newly-produced antitumor metabolites by direct comparison with the control ZBY-3 extract under the guidance of bioassay and thin layer chromatography (TLC), afforded fractions containing **1**–**6** as the main components. Further separation of the fractions by preparative HPLC yielded **1**–**6** (Figure 1).

Compound **1**, 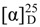 +10.9 (*c* 0.14, MeOH), gave a *pseudo*-molecular ion peak at *m*/*z* 245 [M + H]^+^ in positive ESI-MS. The ^1^H and ^13^C NMR data of **1** in CDCl_3_ were identical with those of cyclo(d-Pro-d-Phe) [50] and cyclo(l-Pro-l-Phe) [51]; however, the positive sign of optical rotation in MeOH was identical with the sign of the former in EtOH [50], but not the negative sign of the latter in MeOH [51]. Thus, **1** was identified as cyclo(d-Pro-d-Phe). Compound **2**, 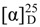 +11.9 (*c* 0.32, MeOH), giving a *pseudo*-molecular ion peak at *m*/*z* 261 [M + H]^+^ in positive ESI-MS, showed ^1^H and ^13^C NMR signals identical with those of cyclo(l-Tyr-l-Pro) in CDCl_3_ [52], but the sign of the optical rotation in MeOH was opposite of the negative sign of cyclo(l-Tyr-l-Pro) in MeOH [52]. Thus, **2** was identified as cyclo(d-Tyr-d-Pro). Compounds **4**, 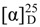 −59.1 (*c* 0.46, MeOH), and **5**, 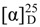 −105.8 (*c* 0.77, MeOH), giving the same *pseudo*-molecular ion peak at *m*/*z* 211 [M + H]^+^ in positive ESI-MS, were identified as cyclo(l-Ile-l-Pro) [53] and cyclo(l-Leu-l-Pro) [54], respectively, by comparison of their physicochemical and spectroscopic data with those in the literature [53,54].

The d and l configuration of the amino acids in **1**–**2** and **4**–**5** was also determined by the Marfey’s method [55] using d- and l-proline (Pro), d- and l-phenylalanine (Phe), d- and l-tyrosine (Tyr), d- and l-isoleucine (Ile) and d- and l-leucine (Leu) as standards. Compounds **1**, **2**, **4** and **5** were hydrolyzed with 6 N HCl at 110 °C for 2 h to produce hydrolysates containing relevant amino acids, and the standards were also treated the same as **1**–**2** and **4**–**5** at the same time and the same conditions, according to the procedure in the literature [30,56]. The hydrolysates of both compounds and standards were then reacted with Marfey’s reagent, 1-fluoro-2,4-dinitrophenyl-5-l-alanineamide (FDAA) [55], at 45 °C for 1 h, to derivatize amino acids in the hydrolysates. HPLC analyses of the Marfey’s derivatives from **1**–**2** and **4**–**5**, using the derivatives of the d- and l-standards as references, demonstrated the d-Pro and d-Phe in **1**, d-Tyr and d-Pro in **2**, l-Ile and l-Pro in **4** and l-Leu and l-Pro in **5**.

Compound **6**, 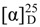 −37 (*c* 0.22, CHCl_3_), was identified as 3β,5α,9α-trihydroxy-(22*E*,24*R*)-ergosta-7,22-dien-6-one on the basis of physicochemical and spectroscopic data [57].

#### 2.4.2. Structural Determination of New Compound **3**

Compound **3**, a crystalline powder from MeOH, melting point (m.p.) 73–75 °C, 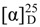 −29.2 (*c* 0.21, MeOH), was assigned the molecular formula C_13_H_15_NO_3_ by HRESIMS (measured 234.1122 [M + H]^+^, calculated for C_13_H_16_NO_3_ [M + H]^+^ 234.1130). The ^1^H and ^13^C NMR spectra of **3** in CD_3_OD showed signals from a mono-alkylated benzene ring and several methylene/methine groups together with the two carbonyl carbon signals in the ^13^C NMR spectrum (Table 3). Analysis of the ^1^H-^1^H COSY, HMQC and HMBC spectra (Table 3) established the planar structure of phenethyl 5-oxoprolinate for **3**. Then, the absolute configuration at C-5 in **3** was assigned to be *S* according to the negative sign of its optical rotation, by comparison with those of 5-oxo-l-proline ([α]_D_ −11.2 (*c* 2.84, H_2_O) in the literature [58]) and methyl 5-oxo-l-prolinate ([α]_D_ −5.6 (*c* 2.8, H_2_O) in the literature [58]). The 5*S* configuration of **3** was also determined further by the Marfey’s method [55], using d- and l-glutamic acid (Glu) as reference standards, by detecting the l-Glu (2*S*-Glu) in the hydrolysate of **3**, as described above for **1**, **2**, **4** and **5**, except for the hydrolysis of **3** with 6 N HCl at 110 °C for 12 h. Thus, the structure of **3** could be determined to be phenethyl 5-oxo-l-prolinate with 5*S* absolute configuration. A literature survey showed that although three patent application publications covered the same planar structure with common structures [59,60,61], only two of them [59,60] really dealt a little with the compound, and no more data were provided, except for the melting point in the literature [60] and the melting point, IR (1735 and 1680 cm^−1^) and ^1^H NMR data in the literature [59]. Although the literature [59] recorded the ^1^H NMR data for the compound that they prepared chemically, these data were quite different from the ^1^H NMR data of **3** (Table 3). Therefore, we report herein **3** as a new compound.

#### 2.4.3. HPLC-PDAD-UV/HPLC-ESI-MS Analyses for Detecting **1**–**6** in u2n2h3-3 Extract

The EtOAc extracts of the mutant, u2n2h3-3, and the strain ZBY-3 were subjected to HPLC-PDAD-UV and HPLC-ESI-MS analyses, using crude **1**–**6** samples as reference standards, to confirm the activated production of **1**–**6** by the introduction of neomycin-resistance in the mutant, u2n2h3-3.

In the HPLC-PDAD-UV analysis, compounds **1**–**3** and **6** were detected in the mutant extract, but not in the control ZBY-3 extract, both by retention times and UV spectra; however, **4** and **5** were hardly identified by the HPLC-PDAD-UV analysis, both in the mutant u2n2h3-3 and the ZBY-3 extracts, because of the lack of their typical UV absorptions and the baseline drift (Figure S3 in the Supplementary Information). In contrast, in the HPLC-ESI-MS analysis, **1**–**6** were all detected in the mutant u2n2h3-3 extract by selective *pseudo*-molecular ion ([M + H]^+^, [M + Na]^+^, [M + NH_4_]^+^, [M − H]^−^ and/or [M + Cl]^−^) monitoring with both extracted ion chromatograms and related MS spectra, but none of these metabolites were detected in the ZBY-3 extract (Figure S4 in the Supplementary Information). These analyses indicated that the production of **1**–**6** in the mutant u2n2h3-3 extract was caused by the activation of silent metabolic pathways in the ZBY-3 strain by the introduction of drug resistance in the mutant. Additional investigations are needed for the interpretation of the affected pathways and the exploration of related mechanisms of their activation.

**Table 3:** Four hundred megahertz ^1^H NMR and 100 MHz ^13^C NMR data of **3** in CD_3_OD ^a^.

Position	δ_C_^b^	δ_H_ (*J* in Hz) ^b^	COSY ^c^	HMBC ^d^
2	181.1 s	-	-	-
3	30.2 t	2.28–2.22 (2H, m, H-3)	H-4	C-2, C-4, C-5
4	25.9 t	2.48–2.34 (1H, m, Ha-4)	H-5	C-2, C-3, C-5, C-6
		2.05–1.94 (1H, m, Hb-4)	H-5	
5	57.1 d	4.23 (1H, dd, *J* = 9.1, 4.2 Hz, H-5)	H_2_-4	C-2, C-3, C-4, C-6
6	173.9 s	-	-	-
7	66.9 t	4.41 (1H, dd, *J* = 10.8, 6.8 Hz, Ha-7)	H-8	C-6, C-8, C-1′
		4.35 (1H, dd, *J* = 10.8, 6.8 Hz, Hb-7)	H-8	
8	35.9 t	2.97 (2H, t, *J* = 6.8 Hz, H_2_-8)	H_2_-7	C-7, C-1′, C-2′,6′
1′	139.1 s	-	-	-
2′,6′	129.5 d	7.18–7.26 (2H, m, H-2′,6′)	H-3′,4′,5′	C-8, C-4′
3′,5′	130.0 d	7.20–7.32 (2H, m, H-3′,5′)	H-2′,4′,6′	C-1′
4′	127.6 d	7.20–7.32 (1H, m, H-4′)	H-2′,6′	C-2′,6′

^a^ Signal assignments were based on the results of ^1^H-^1^H COSY, HMQC and HMBC experiments; ^b^ Chemical shift values (δ_H_ and δ_C_) were recorded using the solvent signals (CD_3_OD: δ_H_ 3.31/δ_C_ 49.0) as references, respectively; ^c^ The numbers in each line of this column indicate the protons that correlated with the proton in the corresponding line in ^1^H-^1^H COSY; ^d^ The numbers in each line of this column indicate the carbons that showed HMBC correlations with the proton in the corresponding line in the HMBC experiments optimized for the 8.3 Hz of the long-range *J*_CH_ value.

#### 2.4.4. Inhibitory Effect of **1**–**6** on Several Human Cancer Cell Lines

Antitumor activities of **1***–***6** were tested by the MTT assay coupled with morphological examination of the tumor cells under an inverted microscope on human cancer K562, HL-60, BGC-823 and HeLa cell lines. In the MTT assay, compounds **1**–**6** inhibited some of the tested four cell lines to varying extents with IR% at 100 µg/mL in Table 4. The half-inhibitory concentrations (IC_50_) of **1**–**3** and **6** on the tested cell lines are shown in Table 5. The positive control, docetaxel, inhibited these cell lines with IR% values of 31.4% (K562), 41.2% (HL-60), 42.2% (BGC-823) and 41.3% (HeLa) at 100 µg/mL.

Prior to MTT treatment of K562, HL-60, BGC-823 and HeLa cells in the MTT assay, the morphology of the cells treated with **1**–**6** at 100 μg/mL were examined under an inverted microscope at first. In the morphological examination, a portion of the HeLa cells treated with **1**–**6**, the HL-60 cells treated with **6** and the BGC-823 cells treated with **1** and **2** showed apoptotic changes in the cell morphology, including cell desquamation and rounding, typical of apoptosis in adherent cells (HeLa and BGC-823), and apoptotic bodies, typical of the apoptosis in suspension cells (HL-60), respectively. Furthermore, larger sized, roundish cells in the suspension of K562 cells treated with **1**, **2** and **5** possessed a major portion, compared to the control K562 cells, revealing the inhibited cell cycle of the K562 cells at the G2/M phase. The others all showed necrotic cell morphology, indicating the cytotoxicity of the compounds on these cells.

**Table 4:** Inhibition rate (IR%) of **1**–**6** on human cancer cells by the MTT assay.

Compound	IR% at 100 µg/mL			
	K562	HL-60	BGC-823	HeLa
1	54.6	92.5	91.6	35.8
2	72.9	94.2	78.1	36.7
3	23.5	50.1	35.1	85.6
4	29.6	24.2	49.4	1.0
5	30.9	28.8	38.3	3.8
6	51.1	90.0	66.5	54.5

**Table 5:** Half-inhibitory concentration (IC_50_) on human cancer cells for **1**–**3** and **6**.

Compound	IC_50_ in µg/mL (µM)			
	K562	HL-60	BGC-823	HeLa
1	97.5 (399.6)	55.0 (225.4)	59.1 (242.2)	>150 (614.7)
2	68.8 (264.6)	53.0 (203.8)	69.0 (265.4)	>150 (576.9)
3	>150 (643.7)	100.0 (429.2)	>150 (643.7)	49.0 (210.3)
6	98.1 (220.9)	39.5 (88.9)	78.9 (177.7)	86.3 (194.4)

### 2.5. Discussions

Neomycin has been identified for weak antioomycete activities on crop oomycete pathogens within several *Phytophthora* species, known as pseudofungi, but not antifungal activities on tested 10 eufungi species, including some of the *Penicillium* and *Aspergillus* species [62]. Indeed, neomycin was also inactive for *Aspergillus* *versicolor* ZBY-3, as shown by our preliminary tests on treatment of the ZBY-3 spores with a high concentration of 400 or 800 mg/mL neomycin in the present study as described. In fact, we also tested, at first, selecting neomycin-resistant mutants from the ZBY-3 strain using drug-containing PDA plates by the routine method in bacterial ribosome engineering to obtain drug-resistant mutants [22]. However, when fresh ZBY-3 spores were spread on PDA plates containing a high concentration of 400 or 800 mg/mL neomycin and incubated at 28 °C for 3–5 days, the ZBY-3 strain grew very well: a lot of fine colony species appeared throughout the PDA plate surface on the second day of incubation and quickly grew into a confluent lawn on the third day of incubation, the same as the growth of the strain on the PDA plates that did not contain neomycin, and no colonies resistant to the neomycin could develop on the PDA plates. These results also indicated the insensitiveness of the ZBY-3 strain to the neomycin. It was thus impossible to obtain drug-resistant mutants from the ZBY-3 strain by neomycin-containing plates, as in bacterial ribosome engineering [22].

The low intracellular concentration of aminoglycosides restricted by fungal membrane permeability, coupled with their lower binding affinity to the eukaryotic rRNA than to the prokaryotic rRNA, was considered to be a major cause resulting in the insensitivity of fungi to aminoglycosides. This has been overcome by the combined use of DMSO on the basis of its effect on membrane permeability to enhance the sensitivity of fungi to aminoglycoside antibiotics, resulting in the successful introduction of gentamicin-resistance into a *Penicillium* species [27]. Instead of the use of DMSO [27], in the present study, we tested the effect of ultrasound on regulating the penetration of the neomycin into fungal cells and found that the treatment of ZBY-3 spores by a high concentration of neomycin in combination with suitable ultrasound irradiation could inhibit strain growth, allowing the development of resistant colonies on PDA plates. This enabled us to carry out single colony isolation to select a total of 30 neomycin-resistant mutants in present study. The acquired resistance of the mutants to neomycin was testified by the resistance test with a mutant, u2n2h3-3, and the parent strain, ZBY-3. After treatment of their spores with the same 200 mg/mL of neomycin as that for selecting the mutant, the mutant grew well on a PDA plate at 28 °C and quickly grew to form confluent lawns early on the fourth day of incubation (Figure 3), whereas the growth of the ZBY-3 strain was significantly inhibited, so as to develop limited colonies on the fourth day of incubation (Figure 3), as also observed in the mutant selection. Thus, the resistance test demonstrated that the neomycin-resistance has been successfully introduced into the mutant, u2n2h3-3.

The effect of the introduction of neomycin resistance on the secondary metabolite production in the ZBY-3 strain was demonstrated both by bioassays and chemical analyses. The EtOAc extract of the ZBY-3 strain did not inhibit K562 cells, whereas those from 22 mutants significantly inhibited K562 cells compared to the ZBY-3 extract (Table 2), indicating that the 22 mutants have acquired the metabolic capability to produce antitumor metabolites. HPLC-PDAD-UV and HPLC-ESI-MS analyses of the EtOAc extracts of seven bioactive mutants and the ZBY-3 stain also supported the altered secondary metabolite production in these mutants (Figures S1 and S2 in the Supplementary Information). This was further evidenced by the following elucidation of six antitumor metabolites **1**–**6** (Figure 1) from one of the seven bioactive mutants, u2n2h3-3, confirming that all of them were newly produced by the mutant compared to the parent ZBY-3 strain (Figures S3 and S4 in the Supplementary Information), which also resulted in the discovery of the new compound **3**. All of **1**–**6** showed inhibitory effects on the K562 cells and also on some of the other three human cancer cell lines tested, to varying extents (Table 4 and Table 5). These results indicated that the production of **1**–**6** in mutant u2n2h3-3 was caused by the introduction of neomycin resistance, activating some metabolic pathways originally silent in parent ZBY-3 strain. It is of biogenetic interest that the production of both dd (**1** and **2**) and ll (**4** and **5**) diketopiperazines was activated in the mutant together with the new compound **3** and the known compound **6** by introduction of the acquired resistance to neomycin. An exploration of the regulatory mechanisms for their activated production requires further investigations into the affected pathways.

A literature survey showed that **3** was a new compound, and all of **1**–**6** were also not yet found in the secondary metabolites of other *Aspergillus* *versicolor* strains, although quite a number of metabolites, including many new compounds with novel skeletal structures, have been reported from *Aspergillus* *versicolor* so far. Diketopiperazines consisting of ll-amino acid residues, such as **4** and **5**, are generally seen in the secondary metabolites of a wide range of common microbial species, but those derived from dd- or dl-amino acid residues were rarely reported from natural sources. To our knowledge, the present study is the first time that the isolation of cyclo(d-Pro-d-Phe) (**1**) and cyclo(d-Pro-d-Tyr) (**2**) from fungal metabolites has been reported, although several dd- or dl-diketopiperazines have so far been reported from marine bacteria [50,51,63] and yeast [64] or marine sponges [65], including **1** from marine bacteria [50]. Further, in the present study, this is also the first time that **2** has been isolated from a natural source, although a chemical preparation of **2** has been recorded in a patent application publication [66].

Compound **1** was initially identified for antibacterial activity on *Vibrio anguillarum* (MIC 0.03 µg/mL) [50] and **2** for the plant disease controlling and plant growth promoting effects by inducing plant pathogen-resistant genes PR-1 and PDF1.2 as signal transducing molecules in plant [66], but no other bioactivities were reported for both compounds. Compound **5** has been reported to show a weak inhibitory effect on the release of β-glucuronidase from rat polymorphonuclear leukocytes induced by platelet-activating factor, though the similar compound **4** did not show the same activity [67]. At the same time, no significant cytotoxicity was recorded for both **4** and **5** on several human cancer cell lines, including the human cancer BGC-823 cell line, in the literature [67]. The very weak inhibitory effect of **4** and **5** on the BGC-823 and the other three human cancer cell lines tested in the present bioassay (Table 4) was consistent with the previous record on BCG-823 and the other four human cancer cell lines in the literature [67]. On the other hand, the present bioassay results for **6** on the human cancer HeLa and the other three human cancer cell lines (Table 4 and Table 5) reconfirmed its cytotoxicity, initially identified in the rat hepatoma HTC cells [68] and HeLa cells [69] in the literature [68,69]. Besides, the present bioassay results for **1**–**3** on the four tested human cancer cell lines were the first record of their cytotoxicity assay, although their effects were not so promising.

Both the above-mentioned bioassays and the chemical investigations in the present study demonstrated the effectiveness of the presented ultrasound-mediated approach to activate silent metabolite production by introducing acquired resistance to aminoglycoside antibiotics in fungi. The present results also showed the potential of this approach for discovering new compounds with antitumor activity from silenced fungal metabolic pathways. This approach could be applied to elicit the metabolic potential of other fungal isolates, to discover new compounds from cryptic secondary metabolites.

## 3. Experimental Section

### 3.1. General Experimental

Melting points were measured on a Beijing Tiandiyu X-4 exact micro melting point apparatus (Tiandiyu Science and Technology Co., Ltd., Beijing, China), and the temperatures were not corrected. Optical rotations were measured on an Optical Activity Limited polAAr 3005 spectropolarimeter (Optical Activity Limited, Ramsey, UK). ESIMS was recorded on an Applied Biosystems API 3000 LC-MS spectrometer (AB SCIEX, Framingham, MA, USA). ^1^H and ^13^C NMR spectra were obtained on a JEOL JNM-GX 400 (400 MHz ^1^H and 100 MHz ^13^C NMR) NMR spectrometer (JEOL Ltd., Tokyo, Japan). The chemical shifts of ^1^H and ^13^C NMR signals were recorded in δ values using the solvent signals (CDCl_3_: δ_H_ 7.26/δ_C_ 77.1; CD_3_OD: δ_H_ 3.31/δ_C_ 49.0) as references, respectively.

Precoated silica gel GF_254_ plates (10 cm × 20 cm, 0.25-mm thickness, Yantai Chemical Industrial Institute, Yantai, China) were used in TLC, and spots were detected under UV lights (254 and 365 nm) or by using a 10% sulfuric acid reagent or Vaughan’s reagent [29,30]. Silica gel H (200–300 mesh, Yantai Chemical Industrial Institute, Yantai, China) and Sephadex™ LH-20 (GE Healthcare, Uppsala, Sweden) were used for column chromatography. HPLC was performed on a Waters HPLC system equipped with Waters 600 controller, Waters 600 pump, Waters 2414 refractive index detector, Waters 2996 (for analytical HPLC) or 2998 (for preparative HPLC) photodiode array detector and Waters Empower™ software (Waters, Milford, MA, USA). A SunFire C_18_ (5 µm, 4.6 mm × 250 mm; Waters, Milford, MA, USA), Venusil MP C_18_ (5 µm, 100 Å, 4.6 mm × 250 mm; Agela Technologies, Tianjin, China) and Senshu Pak C_18_ column (4.6 × 250 mm; Senshu Scientific Co., Ltd., Tokyo, Japan) were used in analytical HPLC, and a Senshu Pak C_18_ column (20 × 250 mm; Senshu Scientific Co., Ltd., Tokyo, Japan) was used in preparative HPLC.

Ultrasound irradiation of the ZBY-3 spores was performed using a JY92-IID ultrasonic cell crusher that produces fixed ultrasonic waves in the 20–25 KHz frequency range and is adjustable in the output powers in a range of 0–900 W (Beijing Hondxinchen Biotechnology Co., Ltd., Beijing, China). ZHWY-2102 rotary shakers (Shanghai ZhiCheng Analyzing Instrument Manufactory Co., Ltd., Shanghai, China) were used for fermentation. A VERSAmax-BN03152 micro plate reader (Molecular Device) was used to read the optical density (OD), and an AE31 EF-INV inverted microscope (Motic China Group Co., Ltd., Xiamen, Fujian, China) was used for the examination of tumor cell morphology.

The Marfey’s reagent, 1-fluoro-2,4-dinitrophenyl-5-l-alanineamide (FDAA), was purchased from J&K Scientific Ltd. (Beijing, China; Lot No. LISOM24). The standard materials used in the Marfey analysis were purchased from ShangHai HanHong Chemical Co., Ltd. (Shanghai, China). For the following standards, the convention is “standard (abbr.), Lot No.”: l-proline (l-Pro), BH-01702-121210; d-proline (d-Pro), BH-01701-120901; l-phenylalanine (l-Phe), BH-01602-120701; d-phenylalanine (d-Phe), BH-01601-121201; l-tyrosine (l-Tyr), BH-02102-140515; d-tyrosine (d-Tyr), BH-02101-140320; l-glutamic acid (l-Glu), BH-00702-110501; d-glutamic acid (d-Glu), BH-00701-110501; l-isoleucine (l-Ile), BH-01205-140610; d-isoleucine (d-Ile), BH-01204-140218; l-leucine (l-Leu), BH-01302-110501; d-leucine (d-Leu), BH-01301-110101.

The human chronic myelogenous leukemia K562 cell line was provided by Prof. Dr. Lili Wang (Beijing Institute of Pharmacology and Toxicology, Beijing, China). Human acute promyelocytic leukemia HL-60, human cervical cancer HeLa and human gastric adenocarcinoma BGC-823 cell lines were provided by Prof. Dr. Wenxia Zhou (Beijing Institute of Pharmacology and Toxicology, Beijing, China). Fetal bovine serum was purchased from Tianjin Hao Yang Biological manufacture Co., Ltd. (Tianjin, China). The RPMI-1640 medium was purchased from Gibco (Lot No. 1403238) and MTT from Amresco (Lot No. 0793). Streptomycin (Lot No. 071104) and penicillin (Lot No. X1103302) were purchased from North China Pharmaceutical Group Corporation, China. Docetaxel (DOC, Lot No. 20110326) was purchased from Beijing Chimivo Technology Co., Ltd. (Beijing, China).

### 3.2. MTT Assay

EtOAc extracts and fractions were dissolved in MeOH at 10 mg/mL, and the MeOH solutions were used in MTT assays. Pure compounds and DOC were dissolved in MeOH to prepare 10.0-mg/mL stock solutions, respectively, and a serial dilution was made for the MTT assay. DOC and MeOH were used as positive and blank controls, respectively.

The MTT assay was performed according to our previous procedure [29,30], and exponentially growing K562, HL-60, HeLa and BGC-823 cells were treated with samples at 37 °C for 24 h. The assay was run in triplicate, and the OD value was read at 570 nm. The IR% was calculated using OD mean values by the formula, IR% = (OD_control_ − OD_sample_)/OD_control_ × 100%, and the IC_50_ value for a sample was obtained from its IR% values at different concentrations.

### 3.3. Original Strain and Spore Suspension Preparation

The fungal strain ZBY-3 that was used as the original strain in present study was isolated from a deep-sea water sample collected at a depth of 800 m in the southeast Pacific (south latitude 24.3444°, east longitude 174.2170°) during the round-the-world ocean research of Dayangyihao in May, 2007. This strain was identified as *Aspergillus versicolor* by sequence analysis of the Internal Transcribed Spacer (ITS) region of the rDNA and by morphological characteristics.

A spore suspension of the ZBY-3 strain was prepared using fresh spores according to the procedure reported [27,28,29,30]. The spore density of the suspension was adjusted by monitoring the OD measured at 600 nm on a VERSAmax-BN03152 micro plate reader to reach a value of 0.35. The spore suspension was used in the following experiments, so that the spore density was kept the same in all of the experiments concerned.

### 3.4. Ultrasound Irradiation of the ZBY-3 Spores

As preliminary tests to search for suitable conditions for introducing neomycin resistance in the ZBY-3 strain, ultrasound irradiation was performed on fresh ZBY-3 spore suspensions with 100, 200, 400 or 800 mg/mL neomycin in water. The different concentration of neomycin was used to examine its effect on the strain growth by treatment of the spores at the tested conditions. A 4-mL Eppendorf tube with 2 mL of the spore suspension was put in an ice water bath, and the cylindrical head of the JY92-IID ultrasonic cell crusher was inserted into the spore suspension approximately 0.5 cm. The ultrasound irradiation was run at 200, 400 or 800 W of output power by 2 s of irradiation every 4 s with an 8-min duration cycle. After ultrasound irradiation, the irradiated spore suspension was saved at 4 °C in a refrigerator for up to 72 h for further treatment of the spores with neomycin. During the treatment period, each 100-µL portion of the treated spore suspension was spread on PDA plates, incubated at 28 °C for 3–5 days and the strain growth examined. An additional three 4-mL Eppendorf tubes each with 2 mL of fresh ZBY-3 spore suspensions without neomycin in water were subjected to ultrasound irradiation at 200, 400 or 800 W of output power by 2 s of irradiation every 4 s with an 8-min duration cycle and treated in the same manner to examine the effect of the ultrasound irradiation on the strain growth. In parallel, another four 4-mL Eppendorf tubes each with 2 mL of fresh ZBY-3 spore suspensions with 100, 200, 400 or 800 mg/mL of neomycin in water were also treated in the same manner, except that the ultrasound irradiation was not performed, to test the effect of neomycin on the strain growth. Furthermore, one 4-mL Eppendorf tube with 2 mL of fresh spore suspension in water was directly kept at 4 °C at the same time and was used as a common control for all of the test tubes mentioned above. In the preliminary tests, ultrasound irradiation of the ZBY-3 spores at 800 W of output power resulted in the complete inhibition of the strain growth on PDA plates at 28 °C, in spite of the presence or absence of neomycin, whereas treatment of the ZBY-3 spores only with 100, 200, 400 or 800 mg/mL of neomycin or only with 200 or 400 W of output power ultrasound irradiation did not affect the strain growth on PDA plates at 28 °C. Thus, all of these treatment methods could not be used for selecting mutant colonies. In contrast, the ultrasound irradiation at 200 or 400 W of output power in the presence of 200 or 400 mg/mL of neomycin properly inhibited strain growth, which was able to be used for selecting drug-resistant mutants formed on the PDA plates by single colony isolation.

Thus, the ultrasound irradiation in the following experiments was routinely run at 200 or 400 W of output power by 2 s of irradiation every 4 s with the 8-min duration cycle on ZBY-3 spore suspensions containing 200 or 400 mg/mL of neomycin in water.

### 3.5. Mutant Selection

To each of the six sterilized 4-mL Eppendorf tubes with or without a duplicate of 400 and 800 mg of neomycin was added 400 μL of the ZBY-3 spore suspension. Then, 1,600 μL of sterilized, distilled water were added to each tube to obtain two series of ZBY-3 spore suspensions with 0, 200 and 400 mg/mL of gentamycin, respectively. The tubes containing neomycin were shaken enough to dissolve the neomycin. The two series of spore suspensions were subjected to ultrasound irradiation at 200 and 400 W of output powers, respectively, as described in Section 3.4. Among them, the four tubes with neomycin were used as test groups, and the two tubes without neomycin were used as controls for the 200- and 400-W ultrasound irradiations, respectively. After ultrasound irradiation, the tubes were immediately saved at 4 °C in a refrigerator for up to 72 h for further treatment of the spores with the neomycin. During the treatment period, each 100-µL portion of the treated spore suspensions was sampled and spread on PDA plates at 3, 12, 24 and 48 h of the treatment and incubated at 28 °C for 3–5 days. Mutants from the test groups were obtained by single colony isolation, selecting colonies with different appearances during the incubation period.

### 3.6. Resistance Test for Acquired Resistance of Mutant u2n2h3-3 to Neomycin

The resistance test was carried out in the same manner and the same conditions for selecting the mutant, u2n2h3-3. Fresh u2n2h3-3 and ZBY-3 spore suspensions with 200 mg/mL neomycin in water were subjected to ultrasound irradiation (200 W output power), as described in Section 3.4, and the spore suspensions were kept at 4 °C for 3 h to further treat the spores with neomycin, as done for selecting the mutant, u2n2h3-3. Each 100 μL of the treated spore suspensions was then spread on PDA plates, incubated at 28 °C and examined for day-by-day growth up to five days.

### 3.7. Fermentation and Sample Preparation for the MTT Assay and Chemical Analysis

The 30 mutants and their parent ZBY-3 strain were inoculated into a 500 mL Erlenmeyer flask containing 200 mL of liquid medium (glucose 2%, maltose 4%, corn flour 0.2%, mannitol 4%, glutamic acid 2%, yeast extract 0.6%, MgSO_4_·7H_2_O 0.06% and KH_2_PO_4_ 0.1% in artificial sea water), respectively, and fermented at 28 °C for 13 days on a rotary shaker at 180 rpm. To each 200 mL of the fermentation broth was added 400 mL of acetone and extracted by ultrasonication for 1.5 h. The aqueous acetone solution obtained by filtration was concentrated under reduced pressure to remove the acetone. Then, the remaining water layer was extracted three times with equal volumes of EtOAc to obtain crude extracts for the MTT assay and chemical analysis (TLC, HPLC-PDAD-UV and HPLC-ESI-MS).

### 3.8. HPLC-PDAD-UV and HPLC-ESI-MS Analyses

The EtOAc extracts of 7 bioactive mutants, u2n2h3-1, u2n2h3-3, u2n2h3-4, u2n4h12-1, u2n4h12-2, u4n4h3-2 and u4n4h24-3, and the ZBY-3 strain were subjected to HPLC-PDAD-UV and HPLC-ESI-MS analyses. The EtOAc extracts dissolved in MeOH at 10.0 mg/mL, which were used in the MTT assay, were also used in both the HPLC-PDAD-UV and HPLC-ESI-MS analyses.

The HPLC-PDAD-UV analysis was performed on an analytical Venusil MP C_18_ column (5 µm, 100 Å, 4.6 mm × 250 mm; Agela Technologies, Tianjin, China) on a Waters HPLC system equipped with a Waters 2996 photodiode array detector (PDAD). Each 10 µL of sample solutions was injected into the column and eluted with a MeOH-H_2_O linear gradient (20% → 100% MeOH in 60 min followed by 30 min with isocratic 100% MeOH) mobile phase (0.8 mL/min flow rate). The acquired photodiode array data were processed by the Empower™ software (Waters, Milford, MA, USA) to obtain the aimed for HPLC chromatograms and UV spectra. Newly-produced secondary metabolites in the mutant extracts were verified both by retention times (*t*_R_) and UV spectra compared to the control ZBY-3 extract.

The HPLC-ESI-MS analysis was performed on an LC-MS equipment equipped with an Agilent 1100 HPLC system, AB Sciex API 3000 LC-MS/MS system and AB Sciex Analyst 1.4 software (AB SCIEX, Framingham, MA, USA). HPLC was carried out on the same Venusil MP C_18_ column (5 µm, 100 Å, 4.6 mm × 250 mm) at the same conditions for HPLC-PDAD-UV analysis. The mass detector was set to scan a range from *m*/*z* 150–1500 in both positive and negative modes. The acquired data were processed by Analyst 1.4 software. Newly-produced secondary metabolites in the mutant extracts were confirmed both by *t*_R_ values and MS spectra compared to the control ZBY-3 extract.

### 3.9. Experiments for Investigation on Compounds 1–6 from Mutant u2n2h3-3

#### 3.9.1. Large-Scale Fermentation and EtOAc Extract Preparation

Mutant u2n2h3-3 is a neomycin-resistant mutant obtained by ultrasound irradiation of the ZBY-3 spores at 200 W of output power in the presence of 200 mg/mL of neomycin. The time course of the mutant fermentation showed that the relative percentage of the mycelia and the inhibitory effect of the EtOAc extract on K562 cells both reached maxima at the 13th day of fermentation, while the pH of the broth remained at 6.0, unchanged throughout the fermentation period tested from the third day of fermentation. According to the results, a large-scale fermentation of the mutant, u2n2h3-3, was carried out for 13 days to obtain the bioactive EtOAc extract, as described below.

An approximately 20-mL spore suspension of the mutant, u2n2h3-3, was prepared using fresh spores, as described in Section 3.3, for the parent ZBY-3 strain. An aliquot (50 µL) of the spore suspension was inoculated into each of 300 Erlenmeyer flasks (500 mL) containing 200 mL of liquid medium (glucose 2%, maltose 4%, corn flour 0.2%, mannitol 4%, glutamic acid 2%, yeast extract 0.6%, MgSO_4_·7H_2_O 0.06% and KH_2_PO_4_ 0.1% in artificial sea water) and fermented at 28 °C for 13 days on a rotary shaker at 180 rpm. The broth (60 L) was filtrated to separate into a filtrate and a mycelial cake. The filtrate (59 L) was extracted three times with equal volumes of EtOAc (3 × 59 L) to give an extract (24.3 g). The mycelial cake was extracted with 3 L acetone-water (2:1) by ultrasonication for 1.5 h. The aqueous acetone solution obtained by filtration was evaporated under reduced pressure to remove the acetone, and the remaining water layer (1 L) was extracted three times with equal volumes of EtOAc (3 × 1 L) to obtain another extract (12 g). Since the extracts, both from the filtrate and the mycelia, showed the same spots on TLC analysis, the two extracts were combined to obtain an EtOAc extract (36.3 g) that inhibited K562 cells with an IR% of 62.9% at 100 μg*/*mL. This extract was used for the isolation of the antitumor metabolites, **1**–**6**.

The parent ZBY-3 strain was also fermented at the same time and the same conditions using two 500-mL Erlenmeyer flasks with 200 mL of the same liquid medium. Extraction of the whole broth (400 mL), as described for the mutant, u2n2h3-3, provided an EtOAc extract (200 mg) that did not inhibit K562 cells (an IR% of 6.8% at 100 µg/mL). This extract was used in the MTT assay and TLC analysis for the separation of **1**–**6** and also in the HPLC-PDAD-UV and HPLC-ESI-MS analyses for detecting **1**–**6** as the negative control.

#### 3.9.2. Isolation of Compounds **1**–**6**

The EtOAc extract (36 g) of mutant u2n2h3-3 was subjected to vacuum liquid chromatography (VLC) on a silica gel column dry-packed with 255 g silica gel (bed 6.5 × 21.2 cm). A stepwise elution by boiling point (b.p.) 60–90 °C petroleum ether (P)-dichloromethane (D) (100:0 → 6:94) → dichloromethane (D)-acetone (A) (100:0 → 6:1) → dichloromethane (D)-methanol (M) (15:1 → 0:100) gave 34 fractions, **Fr-1**–**Fr-34**. Nine of the fractions, **Fr-3** (2.5 g, eluted by PD 20:1), **Fr-6** (3.4 g, eluted by PD 15:1), **Fr-9** (2.2 g, eluted by PD 8:1), **Fr-11** (1.7 g, eluted by PD 2:1), **Fr-13** (0.8 g, eluted by PD 1:5), **Fr-20** (2.1 g, eluted by DA 6:1), **Fr-21** (3.1 g, eluted by DM 15:1), **Fr-22** (1.5 g, eluted by DA 12:1) and **Fr-23** (1.1 g, eluted by DA 10:1), inhibited K562 cells with IR% values of 73.6%, 75.7%, 64.7%, 52.9%, 58.5%, 70.7%, 77.6%, 78.0% and 57.0% at 100 μg/mL, respectively.

**Fr-3** (2.5 g) was subjected to a Sephadex LH-20 column (bed 1.5 × 135 cm in DM 1:1) and eluted with DM (1:1) to obtain 7 fractions, **Fr**-**3-1**–**Fr**-**3**-**7**, in the order of elution. **Fr**-**3**-**3** (450 mg) was separated by VLC on a silica gel column dry-packed with 12 g silica gel (bed 2.5 × 6 cm). A stepwise elution with b.p. 60–90 °C petroleum ether (P)-ethyl acetate (E) (100:0 → 0:100) and then EM (100:0 → 0:100) gave 7 fractions, **Fr**-**3**-**3**-**1**–**Fr**-**3**-**3**-**7**. **Fr**-**3**-**3**-**2** (320 mg, eluted by PE 99:1 → 97:3) gave a crude crystalline precipitate in CH_2_Cl_2_-MeOH solution at room temperature during chromatography. This precipitate was filtered and subjected, without weighing, to preparative HPLC (column: Senshu Pak C_18_ column, 20 × 250 mm; mobile phase: 90% aqueous MeOH; flow rate: 6 mL/min; detector wave length: 246 nm) to obtain **6** (18 mg, *t*_R_ = 28 min) as a white crystalline powder from CH_2_Cl_2_.

**Fr**-**6** (3.4 g) was further separated into 12 fractions, **Fr**-**6**-**1**–**Fr**-**6**-**12**, by VLC on a silica gel column dry-packed with 25 g silica gel (bed 2 × 12 cm) through stepwise elution with PE (100:0 → 0:100) and DM (90:10 → 0:100). **Fr**-**6**-**2** (240 mg, eluted by PE 99:1 → 96:4) was subjected to a Sephadex LH-20 column (bed 1.5 × 135 cm in DM 1:1) and eluted with DM (1:1) to obtain five fractions, **Fr**-**6**-**2**-**1**–**Fr**-**6**-**2**-**5**. **Fr**-**6**-**2**-**3** (50 mg) was separated by preparative HPLC at the same conditions for **6**, except for the use of 40% aqueous MeOH as the mobile phase to obtain **4** (10 mg, *t*_R_ = 31 min), colorless crystals, and **5** (15 mg, *t*_R_ = 33 min), crystalline powder, both from MeOH, respectively.

**Fr**-**9** (2.2 g) was subjected to a Sephadex LH-20 column (bed 1.5 × 135 cm in DM 1:1) and eluted with DM (1:1) to separate into 7 fractions, **Fr**-**9**-**1**–**Fr**-**9**-**7**. **Fr**-**9**-**4** (230 mg) was then separated by preparative HPLC at the same conditions for **6**, except for the use of 50% aqueous MeOH as the mobile phase to obtain **3** (12 mg, *t*_R_ = 27 min) as white crystals from MeOH.

**Fr**-**20** (2.1 g) was separated into 12 fractions, **Fr**-**20**-**1**–**Fr**-**20**-**12**, by VLC on a silica gel column dry-packed with 18 g silica gel (bed 2 × 12 cm) through stepwise elution with PE (100:0 → 0:100) and DM (90:10 → 0:100). **Fr**-**20**-**5** (240 mg, eluted by PE 99:1 → 96:4) was subjected to Sephadex LH-20 column chromatography (bed 1.5 × 135 cm in DM 1:1) eluted with DM (1:1) to obtain five fractions, **Fr**-**20**-**5**-**1**–**Fr**-**20**-**5**-**5**. **Fr**-**20**-**5-3** (140 mg) was separated by preparative HPLC at the same conditions for **6**, except for the use of 45% aqueous MeOH as the mobile phase to obtain **1** (16 mg, *t*_R_ = 35 min), crystalline powder, and **2** (12 mg, *t*_R_ = 38 min), crystalline solid, both from MeOH, respectively.

#### 3.9.3. Physicochemical and Spectroscopic Data of **1**–**6**

Phenethyl 5-oxo-l-prolinate (**3**): Crystalline powder (MeOH), m.p. 73–75 °C. 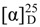 −29.2 (*c* 0.21, MeOH). Positive ion ESIMS *m*/*z*: 234 [M + H]^+^, 256 [M + Na]^+^, 272 [M + K]^+^. Positive HRESIMS *m*/*z*: measured 234.1122 [M + H]^+^, calculated for C_13_H_16_NO_3_ [M + H]^+^ 234.1130; measured 256.0943 [M + Na]^+^, calculated for C_13_H_15_NO_3_Na [M + Na]^+^ 256.0950; measured 272.0681 [M + K]^+^, calculated for C_13_H_15_NO_3_K [M + K]^+^ 272.0689; measured 489.1986 [2M + Na]^+^, calculated for C_26_H_30_N_2_O_6_Na [M + Na]^+^ 489.2002. ^1^H and ^13^C NMR: Table 3.

The data for five known compounds, **1**, **2** and **4**−**6**, are given in the Supplementary Information.

#### 3.9.4. Marfey Analysis for **1**–**5**

To each of six 2-mL ampoules was added 50 µL of **1**–**5** solutions in MeOH at 1 mg/mL, respectively. After blowing inside the ampoules with nitrogen gas to dryness, 100 µL aqueous 6 N HCl were added into each ampoule. After the ampoule was sealed by a blast burner, the ampoules were kept at 110 °C for 2 h to hydrolyze **1**–**5**. Then, the head of the ampoules was cut out; reaction solutions were blown with nitrogen gas to dryness, and the ampoules were maintained *in vacuo* for 2 h to clean up the remaining HCl. At the same time, each 50 µg of the standards, in 50 µL of aqueous 6 N HCl, l-Pro, d-Pro, l-Phe, d-Phe, l-Tyr, d-Tyr, l-Glu, d-Glu, l-Ile, d-Ile, l-Leu and d-Leu, was also treated in the same manner at the same time and same conditions. Both hydrolysates of the standards and **1**–**5** were each dissolved in 10 μL of distilled water, mixed with 10 μL of 10 mM FDAA in acetone and 10 μL of 1 N NaHCO_3_ aqueous solution and then reacted at 45 °C for 1 h to derivatize amino acids in the hydrolysates. The reaction mixtures were neutralized with 5 μL 2 N HCl, respectively. Then, the reaction mixtures were filtered, and the filtrates were subjected to HPLC analysis.

In the above experiments, compound **3** was not hydrolyzed to afford glutamic acid to produce its FDAA derivative. Therefore, additional hydrolysis and derivatization with FDAA were carried out for **3**, by hydrolysis with 6 N HCl at 110 °C for 6, 12 and 24 h, respectively, in the same manner and same conditions. In these experiments, the hydrolysis at 110 °C for 6 h also could not hydrolyze **3**, but the hydrolysis at 110 °C for 12 and 24 h hydrolyzed **3** to produce the aimed at FDAA derivative of glutamic acid. HPLC analysis for **3** was thus carried out on the FDAA derivatives from 12 and 24 h of hydrolysis using the FDAA derivatives of d- and l-Glu as reference standards.

HPLC analysis of the FDAA derivatives was performed on a Watters SunFire C_18_ column (5 µm, 4.6 mm × 250 mm, room temperature) with an acetonitrile-water (containing 0.1% HCOOH) linear gradient (20% → 70% acetonitrile in 30 min → 100% acetonitrile in 10 min, followed by 10 min with isocratic 100% acetonitrile) mobile phase (0.6 mL/min flow rate). The HPLC analysis for the FDAA derivatives from **3** was specially carried out on the same SunFire C_18_ column with a MeOH-H_2_O (containing 0.1% HCOOH) linear gradient (20% → 100% MeOH in 60 min followed by 30 min with isocratic 100% MeOH) mobile phase (0.6 mL/min flow rate) for better separation of the FDAA derivatives of l-Glu and d-Glu. The acquired photodiode array data were processed by Empower™ software (Waters, Milford, MA, USA), and the targeted FDAA derivatives were detected at 340 nm.

In the HPLC conditions, each pair of d- and l-standard FDAA derivatives appeared as separated peaks as given by their retention times (*t*_R_) in Table 6. The FDAA derivatives of the **1**–**5** hydrolysates were analyzed by the injection of the derivatives alone and co-injection with those of related l- and d-standards, respectively.

**Table 6:** Retention times (*t*_R_) for the 1-fluoro-2,4-dinitrophenyl-5-l-alanineamide (FDAA) derivatives of the l- and d-standards.

HPLC Condition ^a^	FDAA Derivative	*t*_R_ (min)	FDAA Derivative	*t*_R_ (min)
A	l-proline (Pro)	28.77	d-proline (Pro)	29.75
**A**	l-phenylalanine (Phe)	38.47	d-phenylalanine (Phe)	40.33
A	l-tyrosine (Tyr)	30.27	d-tyrosine (Tyr)	31.37
A	l-glutamic acid (Glu)	28.07	d-glutamic acid (Glu)	28.50
B	l-glutamic acid (Glu)	52.65	d-glutamic acid (Glu)	56.87
A	l-isoleucine (Ile)	35.13	d-isoleucine (Ile)	37.55
A	l-leucine (Leu)	35.57	d-leucine (Leu)	39.37

^a^ HPLC column: Watters SunFire C_18_ column (5 µm, 4.6 mm × 250 mm), room temperature. A and B indicate the mobile phases as follows. A: acetonitrile-water (containing 0.1% HCOOH) in a linear gradient, 20% → 70% acetonitrile in 30 min → 100% acetonitrile in 10 min followed by 10 min with isocratic 100% acetonitrile, and a 0.6 mL/min flow rate; B: MeOH-H_2_O (containing 0.1% HCOOH) in a linear gradient, 20% → 100% MeOH in 60 min followed by 30 min with isocratic 100% MeOH, and a 0.6 mL/min flow rate.

#### 3.9.5. Examination of **1**–**6** in EtOAc Extracts of Mutant u2n2h3-3 and the Parent ZBY-3 Strain

Examination of **1**–**6** in the EtOAc extracts of mutant u2n2h3-3 and strain ZBY-3 was carried out by HPLC-PDAD-UV and HPLC-ESI-MS analyses. A crude **1**–**6** sample in MeOH at 10 and 1 mg/mL was used as the reference in the HPLC-PDAD-UV and HPLC-ESI-MS analyses, respectively. HPLC-PDAD-UV and HPLC-ESI-MS were performed at the conditions given in Section 3.8.

In the HPLC-PDAD-UV analysis, **1**–**6** appeared as peaks with retention times of 30.4 min for **1**, 17.5 min for **2**, 37.7 min for **3**, 26.3 min for **4**, 26.6 min for **5** and 69.8 min for **6**. Compounds **1**–**3** and **6** were detected in the mutant extract, but not in the ZBY-3 extract, both by retention times and UV spectra; however, **4** and **5** were hardly identified by the HPLC-PDAD-UV analysis, both in the mutant and ZBY-3 extracts, because of the lack of their typical UV absorptions and the baseline drift (Figure S3 in the Supplementary Information).

In the HPLC-ESI-MS analysis, retention times of the **1**–**6** ion peaks were slightly shortened compared to those in the HPLC-PDAD-UV analysis, because of the shortened flow length from the outlet of HPLC column to the inlet of MS in the HPLC-ESI-MS. Their ion peaks appeared at the retention times of 23–26 min for **1**, 13–15 min for **2**, 31–33 min for **3**, 20.5–22.5 min for **4**, 21–23 min for **5** and 63–65 min for **6**. By selective ion ([M + H]^+^, [M + Na]^+^, [M + NH_4_]^+^, [M − H]^−^ and/or [M + Cl]^−^) monitoring with both the extracted ion chromatograms and the related MS spectra, **1**–**6** were all detected in the mutant u2n2h3-3 extract, but none of these metabolites were detected in the ZBY-3 extract (Figure S4 in the Supplementary Information).

## 4. Conclusions

An ultrasound-mediated new approach to introduce drug-resistance to activate silenced metabolic pathways in fungi has been developed by work on a bio-inactive, deep-sea fungus, *A. versicolor* ZBY-3. Upon treatment of the ZBY-3 spores with a high concentration of neomycin under proper ultrasound irradiation, a total of 30 mutants were obtained by single colony isolation. The acquired resistance of the mutants to neomycin was confirmed by the resistance test. Twenty two of the 30 mutants acquired the metabolic capability to produce antitumor metabolites, as indicated by the antitumor effects of their EtOAc extracts, which was evidenced also by HPLC-PDAD-UV and HPLC-ESI-MS analyses of the extracts of the ZBY-3 strain and the seven bioactive mutants. Elucidation of six newly-produced antitumor metabolites by mutant u2n2h3-3 compared to the parent ZBY-3 strain resulted in the discovery of a new compound. Both bioassays and chemical investigations demonstrated the effectiveness of this approach to activate silent metabolite production and the potential for discovering new antitumor compounds from silenced fungal metabolic pathways, which could be applied to other fungal metabolite studies.

## Supplementary Files

Supplementary File 1
