# Supplementary material for: Activation of Dormant Secondary Metabolite Production by Introducing Neomycin Resistance into the Deep-Sea Fungus, Aspergillus versicolor ZBY-3
Source: Mar Drugs. 2014 Jul 29;12(8):4326–52. doi: 10.3390/md12084326 (PMC4145319; doi:10.3390/md12084326)
Supplement: Supplementary File 1 [file marinedrugs-12-04326-s001.pdf]

Supplementary Information

S1. Physicochemical and spectroscopic data of five known compounds **1**, **2** and **4**–**6** S2

Figure S1. HPLC-PDAD-UV analysis of the EtOAc extracts of the control ZBY-3 strain and selected mutants S4

Figure S2. HPLC-ESI-MS analysis of the EtOAc extracts of the control ZBY-3 strain and selected mutants S12

Figure S3. HPLC-PDAD-UV analysis of **1**–**6** and the EtOAc extracts of the mutant u2n2h3-3 and the control ZBY-3 strain S48

Figure S4. HPLC-ESI-MS analysis of **1**–**6** and the EtOAc extracts of the mutant u2n2h3-3 and the control ZBY-3 strain S50

Figure S5. Positive ESI-MS and HR-ESI-MS spectra of **3** S56

Figure S6. ^{1}H NMR spectrum of **3** in CD_3OD S57

Figure S7. ^{13}C NMR spectrum of **3** in CD_3OD S58

Figure S8. ^{1}H-^{1}H COSY spectrum of **3** in CD_3OD S59

Figure S9. HMQC spectrum of **3** in CD_3OD S60

Figure S10. HMBC spectrum of **3** in CD_3OD S61

S1. Physicochemical and Spectroscopic Data of Five Known Compounds 1, 2 and 4–6

Cyclo(D-Pro-D-Phe) (**1**): Crystalline powder (MeOH), m.p. 150–151 °C, $[\alpha]_D^{25}$ +10.9 (*c* 0.14, MeOH). Positive ion ESIMS *m*/*z*: 245 $[M + H]^+$, 267 $[M + Na]^+$, 283 $[M + K]^+$; negative ion ESIMS *m*/*z*: 243 $[M - H]^-$. Positive HRESIMS *m*/*z*: measured 245.1283 $[M + H]^+$, calcd for $C_{14}H_{17}N_2O_2$ $[M + H]^+$ 245.1290; measured 267.1101 $[M + Na]^+$, calcd for $C_{14}H_{16}N_2O_2Na$ $[M + Na]^+$ 267.1109. ^{1}H NMR (400 MHz, $CDCl_3$) δ: 7.39–7.20 (5H, m, H-2′−H-6′), 5.63 (1H, br s, 8-NH), 4.28 (1H, dd, *J* = 10.7, 2.8 Hz, H-9), 4.08 (1H, br t, *J* = 7.5 Hz, H-6), 3.71–3.53 (3H, m, H_2-3, Ha-10), 2.77 (1H, dd, *J* = 14.5, 10.8 Hz, Hb-10), 2.38–2.30 (1H, m, Hb-5), 2.07–1.84 (3H, m, H_2-4, Ha-5). ^{13}C NMR (100 MHz, $CDCl_3$) δ: 169.6 (C-1), 165.2 (C-7), 136.0 (C-1′), 129.5 (2C, C-2′,6′), 129.2 (2C, C-3′5′), 127.7 (C-4′), 59.3 (C-6), 56.3 (C-9), 45.6 (C-3), 36.9 (C-10), 28.5 (C-5), 22.7 (C-4).

Cyclo-(D-Pro-D-Tyr) (**2**): Crystalline solid (MeOH), m.p. 147–149 ℃, $[\alpha]_D^{25}$ +11.9 (*c* 0.32, MeOH). Positive ion ESIMS *m*/*z*: 261 $[M + H]^+$, 283 $[M + Na]^+$, 299 $[M + K]^+$; negative ion ESIMS *m*/*z*: 259 $[M - H]^-$. Positive HRESIMS *m*/*z*: measured 261.1231 $[M + H]^+$, calcd for $C_{14}H_{17}N_2O_3$ $[M + H]^+$ 261.1239; measured 283.1049 $[M + Na]^+$, calcd for $C_{14}H_{16}N_2O_3Na$ $[M + Na]^+$ 283.1059; measured 543.2210 $[2M + Na]^+$, calcd for $C_{28}H_{32}N_4O_6Na$ $[2M + Na]^+$ 543.2220. ^{1}H NMR (400 MHz, $CDCl_3$) δ: 7.08 (2H, d, *J* = 8.3 Hz, H-2′,6′), 6.80 (2H, d, *J* = 8.3 Hz, H-3′,5′), 5.63 (1H, br s, 8-NH), 4.21 (1H, br d, *J* = 8.8 Hz, H-9), 4.09 (1H, t, *J* = 7.50 Hz, H-6), 3.69–3.49 (3H, m, H_2-3, Hb-10), 2.73 (1H, dd, *J* = 14.5, 10.6 Hz, Ha-10), 2.43–2.22 (1H, m, Hb-5), 1.99–1.86 (3H, m, H_2-4, Hb-5). ^{13}C NMR (100 MHz, $CDCl_3$) δ: 169.6 (C-1), 165.2 (C-7), 155.4 (C-4′), 130.5 (2C, C-2′,6′), 127.7 (C-1′), 116.3 (2C, C-3′,5′), 59.6 (C-6), 56.3 (C-9), 45.6 (C-3), 36.0 (C-10), 28.5 (C-5), 22.8 (C-4).

Cyclo(L-Ile-L-Pro) (**4**): Crystalline powder (MeOH), m.p. 107–110 °C. $[\alpha]_D^{25}$ +59.1 (*c* 0.46, MeOH). Positive ion ESIMS *m*/*z*: 211 $[M + H]^+$, 233 $[M + Na]^+$; negative ion ESIMS *m*/*z*: 209 $[M - H]^-$, 245 $[M + HCOO]^-$. ^{1}H NMR (400 MHz, $CDCl_3$) δ: 5.97 (1H, br s, 8-NH), 4.07 (1H, br t, *J* = 8.1 Hz, H-6), 3.96 (1H, br s, H-9), 3.67–3.50 (2H, H_2-3), 2.42–2.35 (1H, m, H-10), 2.35–2.26 (1H, m, Ha-5), 2.10–1.97 (2H, m, Ha-4, Hb-5), 1.96-1.82 (1H, m, Hb-4), 1.48–1.36 (1H, m, Ha-11), 1.26–1.10 (1H, m, Hb-11), 1.05 (3H, d, *J* = 7.2 Hz, H_3-13), 0.92 (3H, t, *J* = 7.4 Hz, H_3-12).^{13}C NMR (100 MHz, $CDCl_3$) δ: 170.0 (C-1), 165.1 (C-7), 60.6 (C-9), 58.9 (C-6), 45.3 (C-3), 35.4 (C-10), 28.7 (C-5), 24.1 (C-11), 22.5 (C-4), 16.1 (C-13), 12.2 (C-12).

Cyclo(L-Leu-L-Pro) (**5**): Crystalline powder (MeOH), m.p. 148–149 °C, $[\alpha]_D^{25}$ +105.8 (*c* 0.77, MeOH). Positive ion ESIMS *m*/*z*: 211 $[M + H]^+$, 233 $[M + Na]^+$; negative ion ESIMS *m*/*z*: 209 $[M - H]^-$, 255 $[M + HCOO]^-$. ^{1}H NMR (400 MHz, $CDCl_3$) δ: 6.18 (1H, s, 8-NH), 4.11 (1H, t, *J* = 7.5 Hz, H-6), 4.01 (1H, dd, *J* = 9.6, 3.4 Hz, H-9), 3.63–3.49 (2H, m, H_2-3), 2.34 (1H, dtd, *J* = 9.7, 6.8, 2.9 Hz, Hb-5), 2.18–1.97 (3H, m, Hb-4, Ha-5, Ha-10), 1.96–1.84 (1H, m, Ha-4), 1.82–1.69 (1H, m, H-11), 1.52 (1H, ddd, *J* = 14.5, 9.6, 4.9 Hz, Hb-10), 0.99 (3H, d, *J* = 6.6 Hz, H_3-13), 0.94 (3H, d, *J* = 6.6 Hz, H_3-12). ^{13}C NMR (100 MHz, $CDCl_3$) δ: 170.4 (C-1), 166.3 (C-7), 59.1 (C-6), 53.5 (C-9), 45.6 (C-3), 38.7 (C-10), 28.2 (C-5), 24.8 (C-11), 23.4 (C-4), 22.9 (C-12), 21.3 (C-13).

3β,5α,9α-Trihydroxy-(22*E*,24*R*)-ergosta-7,22-dien-6-one (**6**): Crystalline powder (CH_2Cl_2), m.p. 218–219 °C. $[\alpha]_D^{25}$ −37 (*c* 0.22, $CHCl_3$). Positive ion ESIMS *m*/*z*: 467 $[M + Na]^+$, 483 $[M + K]^+$; negative ion ESIMS *m*/*z*: 443 $[M - H]^-$, 479 $[M + Cl]^-$. ^{1}H NMR (400 MHz, CD_3OD) δ: 5.59 (1H, d,

J = 2.0 Hz, H-7), 5.27 (1H, dd, *J* = 15.2, 7.1 Hz, H-23), 5.21 (1H, dd, *J* = 15.2, 7.7 Hz, H-22), 3.97–3.89 (1H, m, H-3), 1.06 (3H, d, *J* = 6.6 Hz, H_3-21), 1.00 (3H, s, H_3-19), 0.95 (3H, d, *J* = 6.8 Hz, H_3-28), 0.87 (3H, d, *J* = 7.0 Hz, H_3-27), 0.85 (3H, d, *J* = 7.0 Hz, H_3-26), 0.67 (3H, s, H_3-18). ^{13}C NMR (100 MHz, CD_3OD) δ: 200.1 (C-6), 165.0 (C-8), 136.7 (C-22), 133.6 (C-23), 120.9 (C-7), 80.2 (C-5), 76.1 (C-9), 67.8 (C-3), 57.4 (C-17), 52.8 (C-14), 46.2 (C-13), 44.4 (C-24), 42.7 (C-10), 41.7 (C-20), 37.1 (C-4), 36.2 (C-12), 34.4 (C-25), 31.0 (C-2), 29.3 (C-11), 29.1 (C-16), 26.6 (C-1), 23.4 (C-15), 21.6 (C-21), 20.54 (C-19), 20.47 (C-26), 20.1 (C-27), 18.2 (C-28), 12.6 (C-18).

Figure S1. HPLC-PDAD-UV analysis of the EtOAc extracts of the control ZBY-3 strain and selected mutants. (**A**) HPLC profiles detected at 210 nm; (**B**) Enlarged HPLC profiles detected at 210 nm (from 35 min to 60 min); (**C**) HPLC profiles detected at 254 nm; (**D**) Enlarged HPLC profiles detected at 254 nm (from 35 min to 60 min); (**E**) HPLC profiles detected at 290 nm; (**F**) Enlarged HPLC profiles detected at 290 nm (from 35 min to 60 min); (**G1**) UV spectra of selected new peaks in the mutant extracts, and UV absorptions of the control G59 extract at the corresponding retention times (t_R); (**G2**) UV spectra of selected new peaks in the mutant extracts, and UV absorptions of the control G59 extract at the corresponding retention times (t_R).

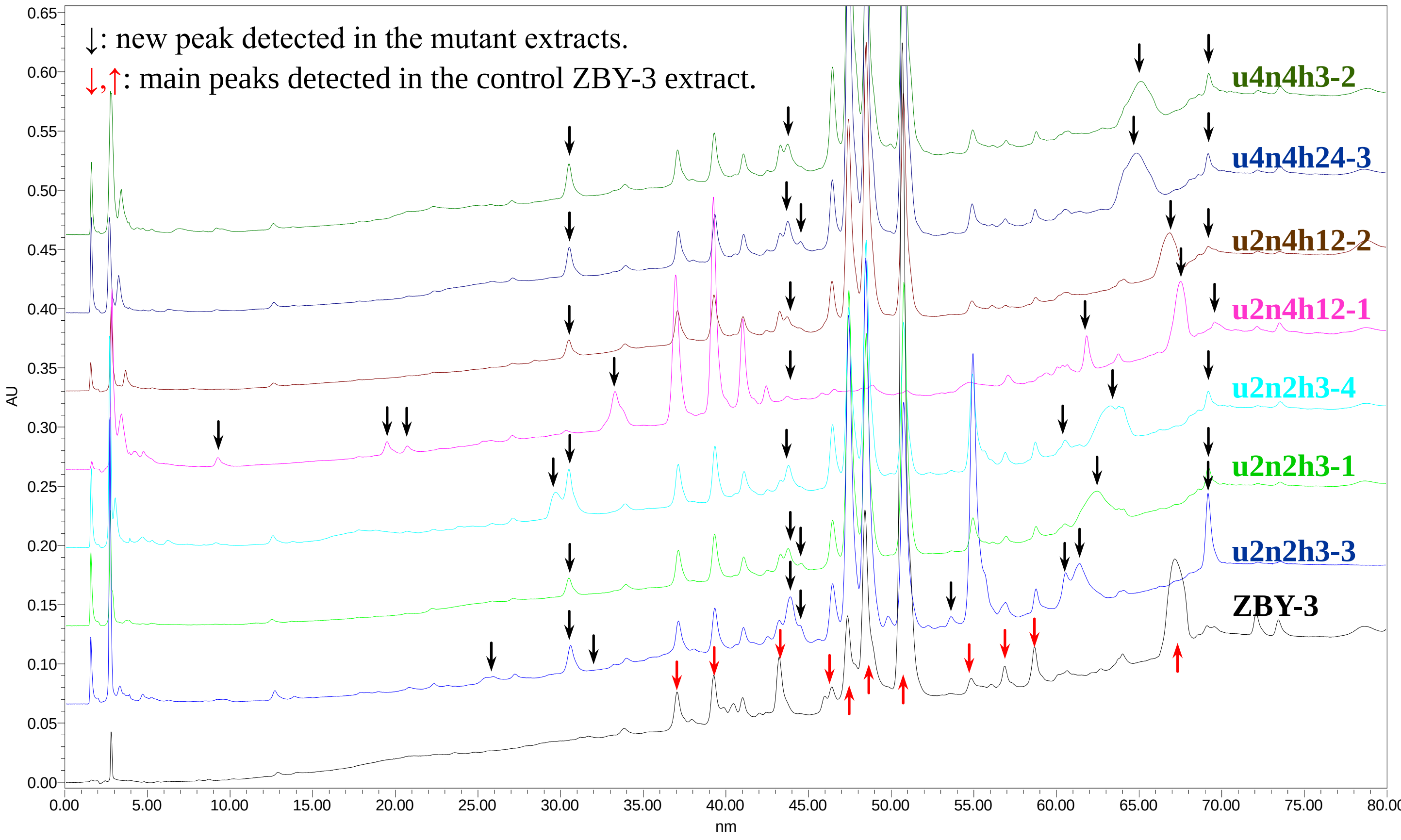

(**A**)

Figure S1. *Cont.*

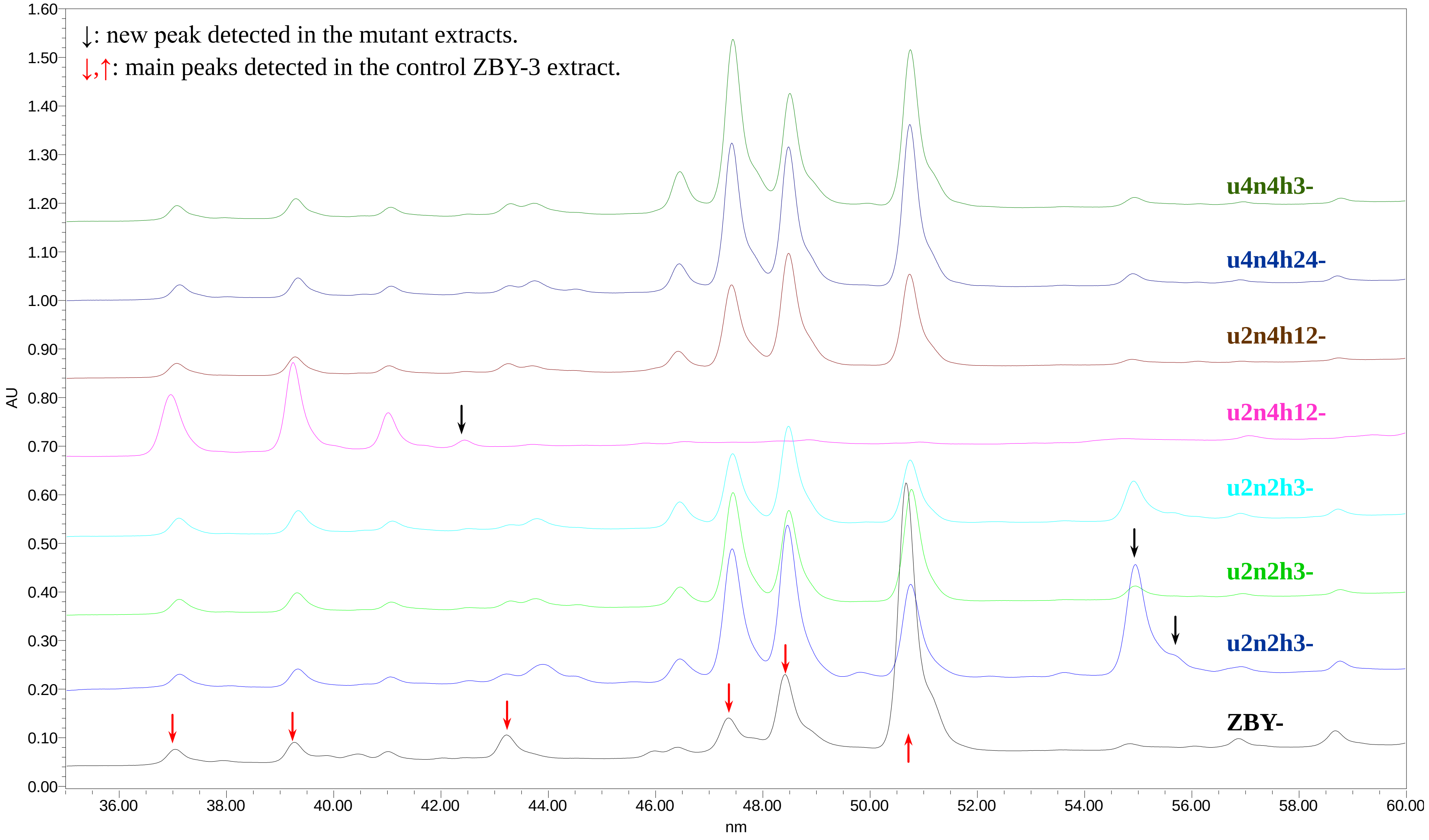

(**B**)

Figure S1. *Cont.*

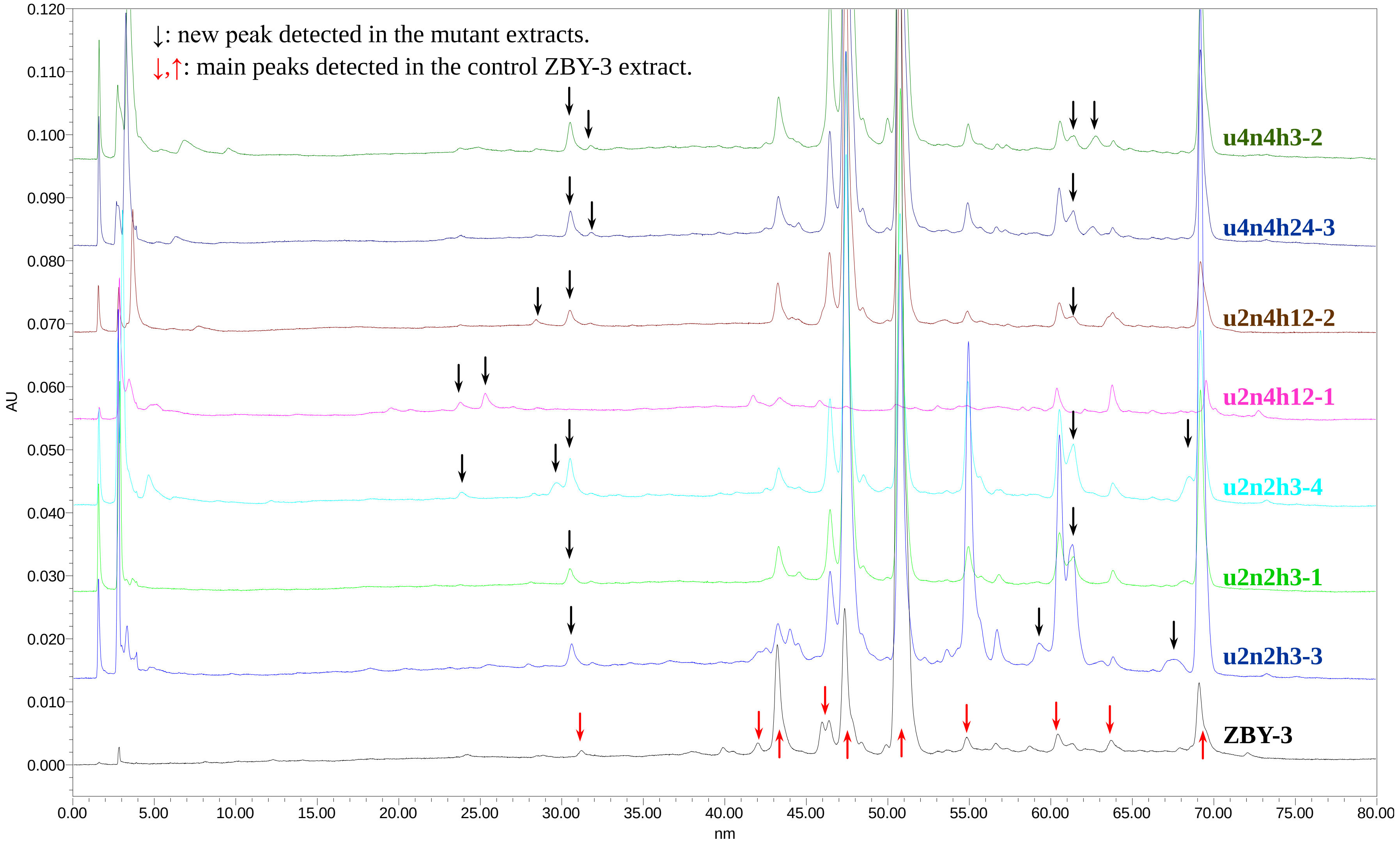

(C)

Figure S1. *Cont.*

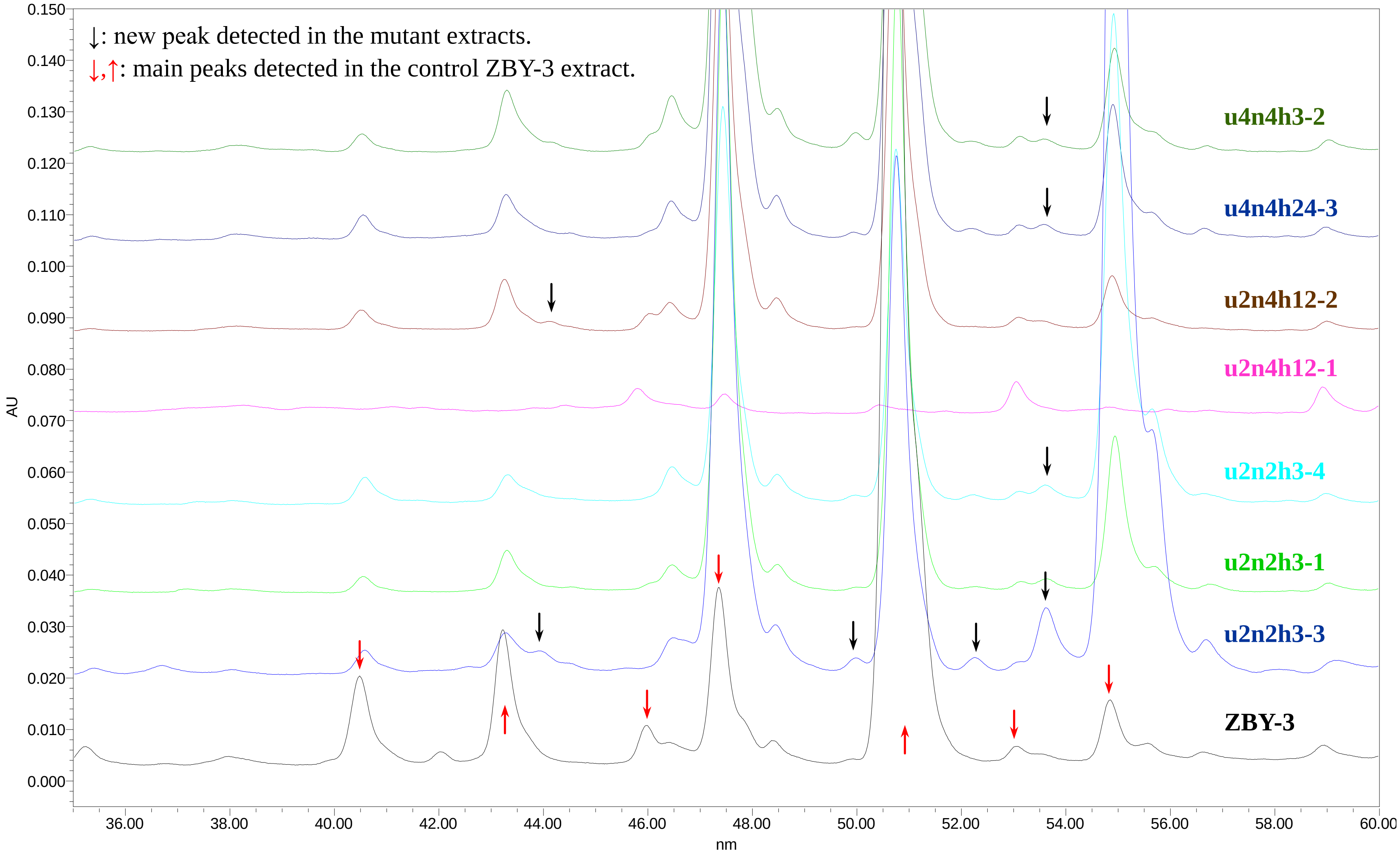

(D)

Figure S1. *Cont.*

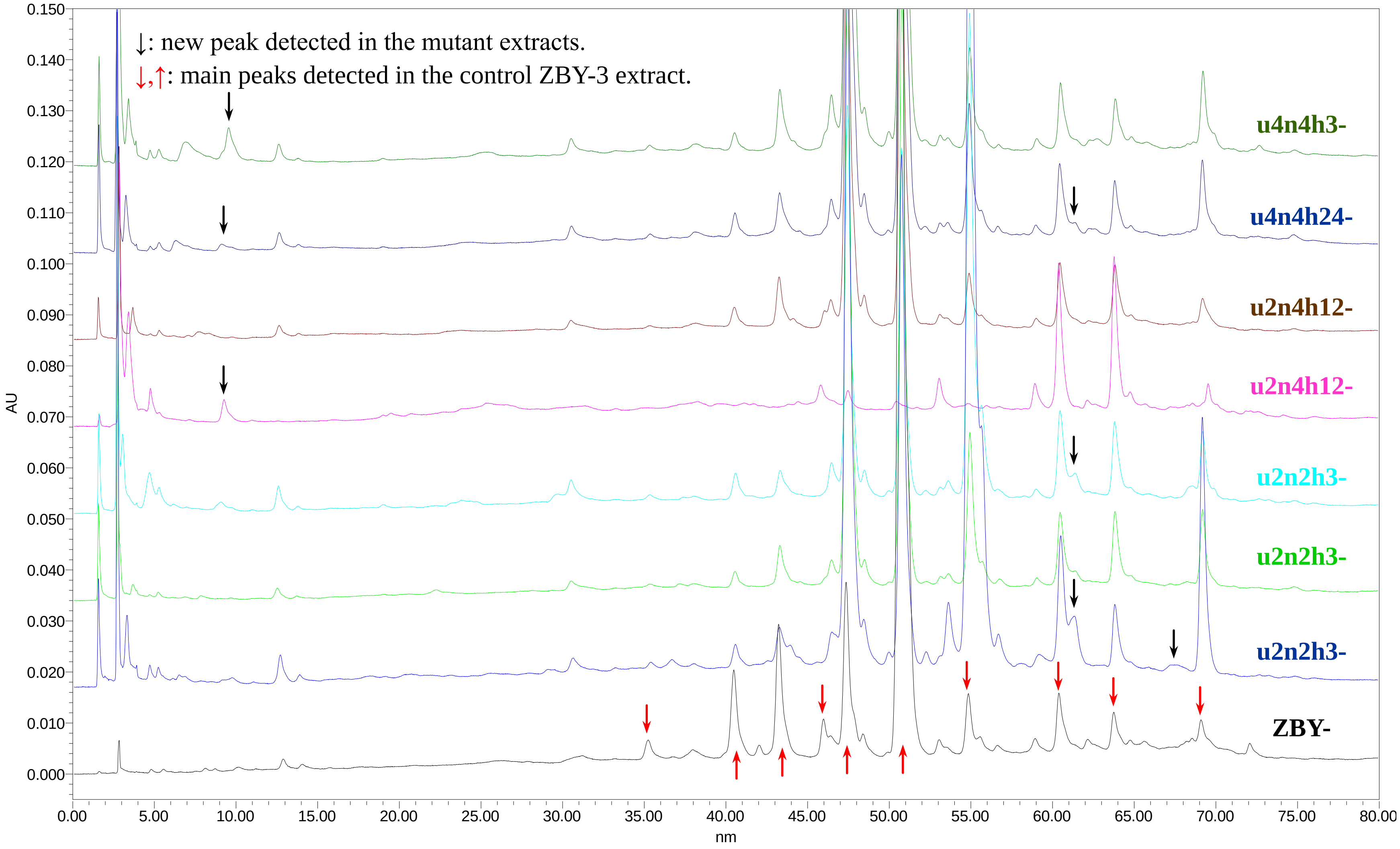

(E)

Figure S1. *Cont.*

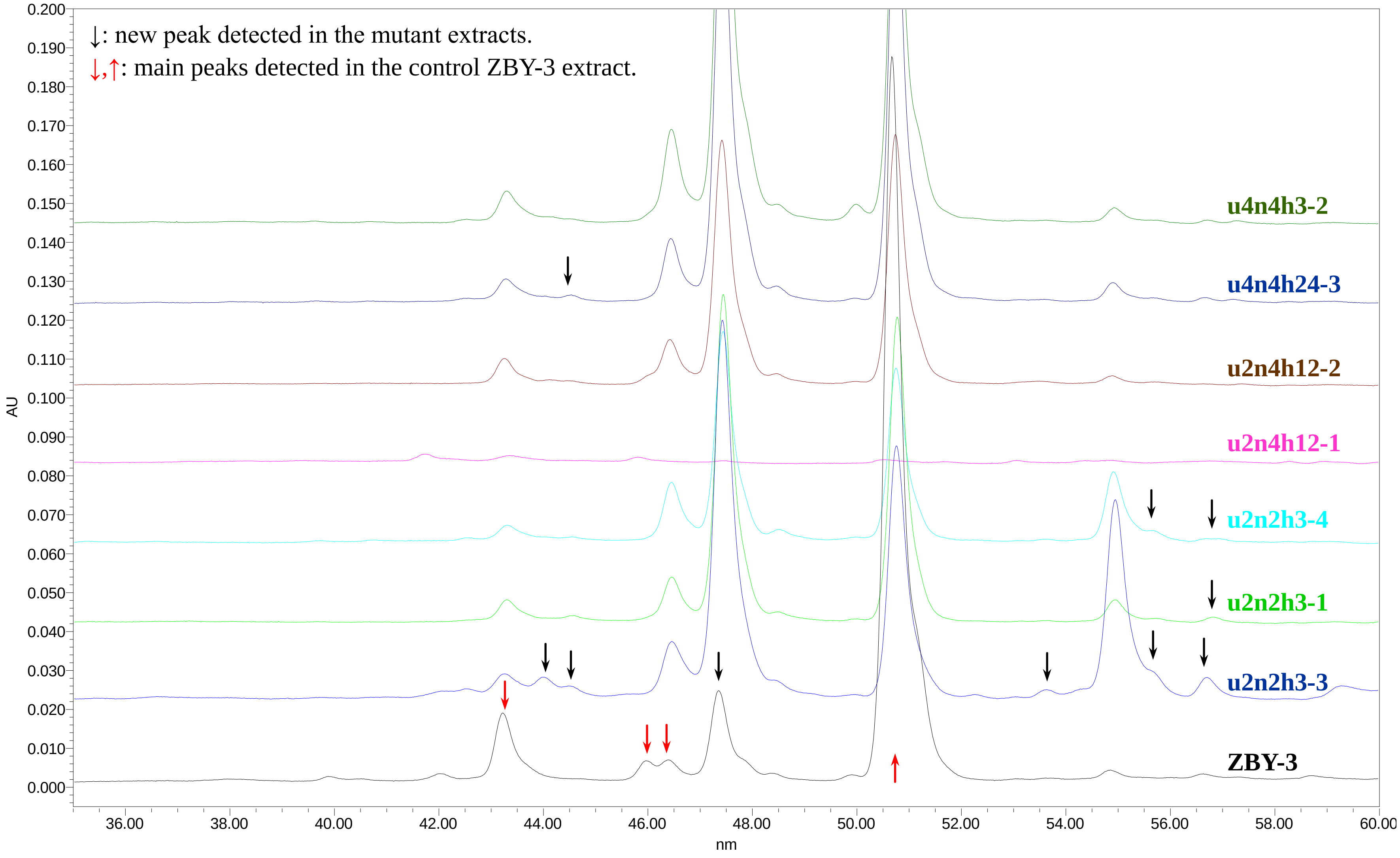

(F)

Figure S1. *Cont.*

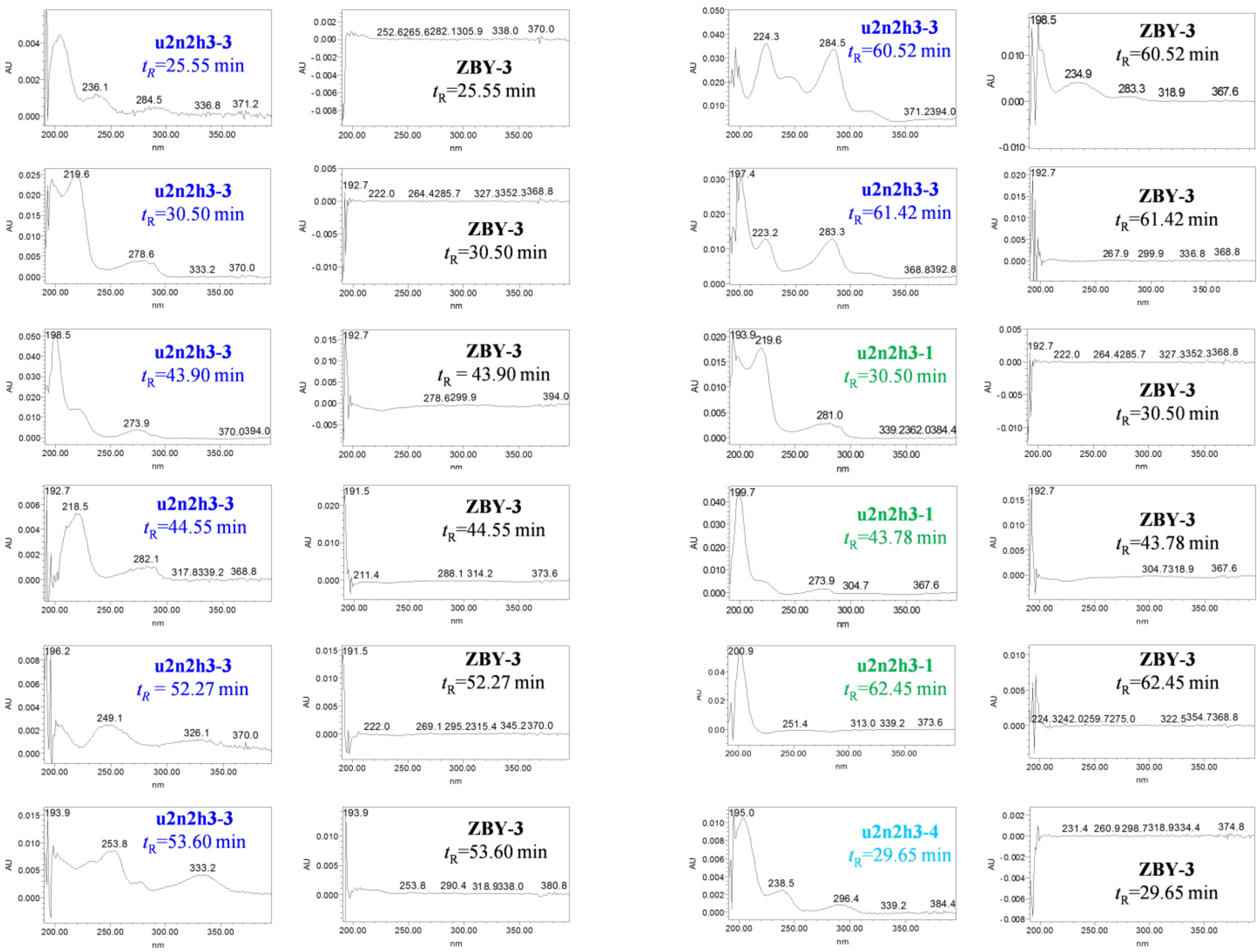

(G1)

Figure S1. *Cont.*

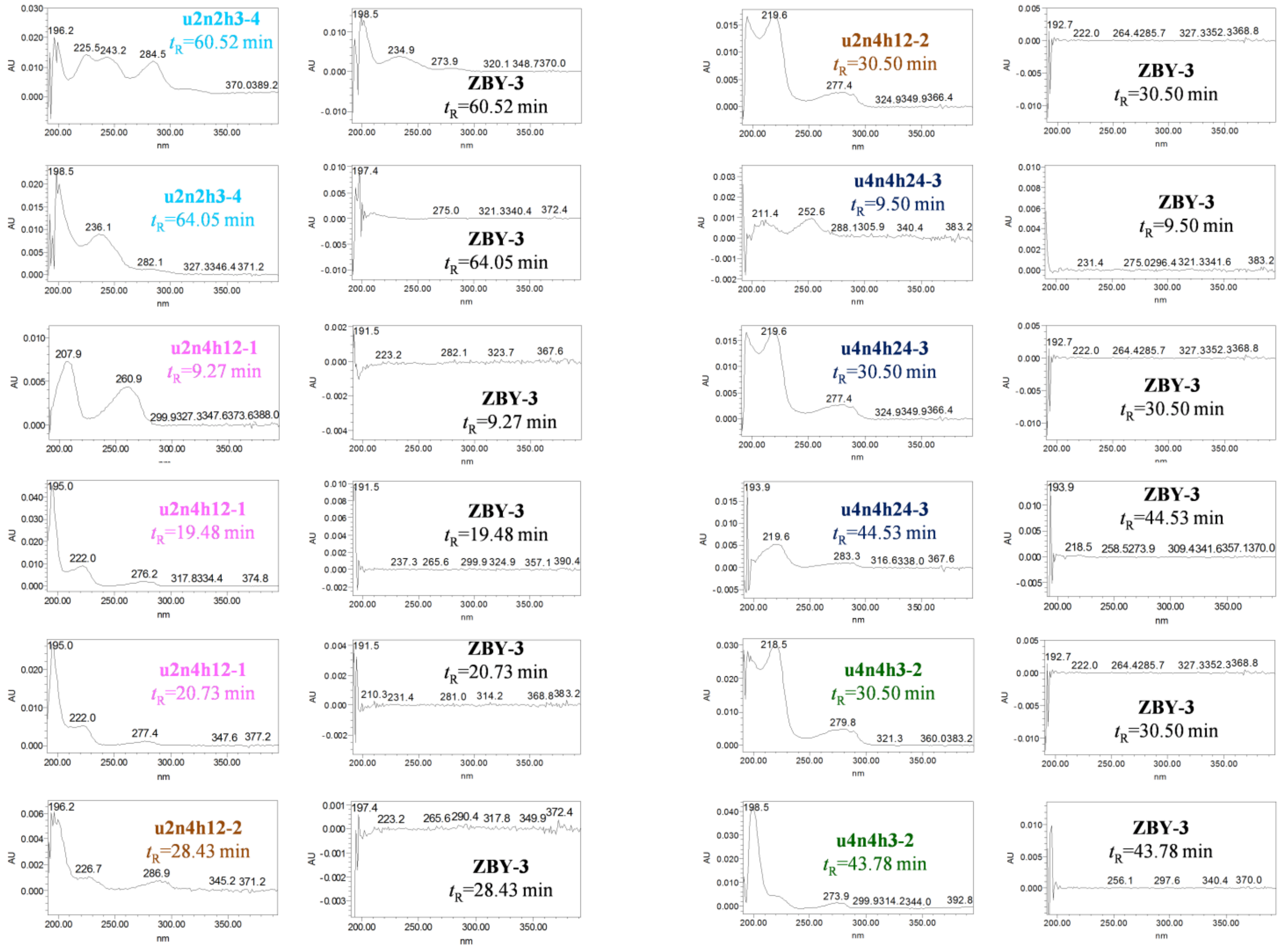

(G2)

Figure S2. HPLC-ESI-MS analysis of the EtOAc extracts of the control ZBY-3 strain and selected mutants. (**A**) Total ion chromatograms detected by positive ESI-MS; (**B1**) Total ion chromatograms detected by negative ESI-MS; (**B2**) Total ion chromatograms (TIC) for the ZBY-3 strain and a mutant u2n2h3-3; (**C1**) Extracted positive ion (*m*/*z* 200.0–300.0) chromatograms; (**C2**) Extracted positive ion (*m*/*z* 300.0–400.0) chromatograms; (**C3**) Extracted positive ion (*m*/*z* 400.0–500.0) chromatograms; (**C4**) Extracted positive ion (*m*/*z* 500.0–600.0) chromatograms; (**C5**) Extracted positive ion (*m*/*z* 600.0–700.0) Chromatograms; (**C6**) Extracted positive ion (*m*/*z* 700.0–800.0) chromatograms; (**C7**) Extracted positive ion (*m*/*z* 800.0–900.0) chromatograms; (**C8**) Extracted positive ion (*m*/*z* 900.0–1000.0) chromatograms; (**D1**) Extracted negative ion (*m*/*z* 200.0–300.0) chromatograms; (**D2**) Extracted negative ion (*m*/*z* 300.0–400.0) chromatograms; (**D3**) Extracted negative ion (*m*/*z* 400.0–500.0) chromatograms; (**D4**) Extracted negative ion (*m*/*z* 500.0–600.0) chromatograms; (**D5**) Extracted negative ion (*m*/*z* 600.0–700.0) chromatograms; (**D6**) Extracted negative ion (*m*/*z* 700.0–800.0) chromatograms; (**D7**) Extracted negative ion (*m*/*z* 800.0–900.0) chromatograms; (**D8**) Extracted negative ion (*m*/*z* 900.0–1000.0) chromatograms; (**E1**) Extracted ion chromatograms (EIC, *m*/*z* 200.0–300.0) for the ZBY-3 strain and a mutant u2n2h3-3; (**E2**) Extracted ion chromatograms (EIC, *m*/*z* 300.0–400.0) for the ZBY-3 strain and a mutant u2n2h3-3; (**E3**) Extracted ion chromatograms (EIC, *m*/*z* 400.0–500.0) for the ZBY-3 strain and a mutant u2n2h3-3; (**E4**) Extracted ion chromatograms (EIC, *m*/*z* 500.0–600.0) for the ZBY-3 strain and a mutant u2n2h3-3; (**E5**) Extracted ion chromatograms (EIC, *m*/*z* 600.0–700.0) for the ZBY-3 strain and a mutant u2n2h3-3; (**E6**) Extracted ion chromatograms (EIC, *m*/*z* 700.0–800.0) for the ZBY-3 strain and a mutant u2n2h3-3; (**E7**) Extracted ion chromatograms (EIC, *m*/*z* 800.0–900.0) for the ZBY-3 strain and a mutant u2n2h3-3; (**E8**) Extracted ion chromatograms (EIC, *m*/*z* 900.0–1000.0) for the ZBY-3 strain and a mutant u2n2h3-3; (**F1**) HPLC-positive ion ESI-MS analysis (*m/z* 300.0−400.0; t_R = 25.71−26.41 min); (**F2**) HPLC-positive ion ESI-MS analysis (*m/z* 500.0−600.0; t_R = 69.11 min); (**F3**) HPLC-positive ion ESI-MS analysis (*m/z* 700.0−800.0; t_R = 70.19 min); (**F4**) HPLC-positive ion ESI-MS analysis (*m/z* 700.0−800.0; t_R = 76.98 min); (**F5**) HPLC-positive ion ESI-MS analysis (*m/z* 300.0−400.0; t_R = 38.68 min); (**F6**) HPLC-positive ion ESI-MS analysis (*m/z* 500.0−600.0; t_R = 69.33 min); (**F7**) HPLC-positive ion ESI-MS analysis (*m/z* 200.0−300.0; t_R=14.95 and 19.19 min); (**F8**) HPLC-positive ion ESI-MS analysis (*m/z* 300.0−400.0; t_R = 40.49, 46.68 and 51.12 min); (**F9**) HPLC-positive ion ESI-MS analysis (*m/z* 900.0−1000.0; t_R = 51.15 and 55.79 min); (**F10**) HPLC-positive ion ESI-MS analysis (*m/z* 200.0−300.0; t_R = 21.85 and 23.90 min); (**F11**) HPLC-positive ion ESI-MS analysis (*m/z* 400.0−500.0; t_R = 42.52 and 61.51 min); (**F12**) HPLC-positive ion ESI-MS analysis (*m/z* 300.0−400.0; t_R = 4.41 and 20.04 min); (**F13**) HPLC-positive ion ESI-MS analysis (*m/z* 300.0−400.0; t_R = 21.97 and 24.83 min); (**F14**) HPLC-positive ion ESI-MS analysis (*m/z* 400.0−500.0; t_R = 39.28 and 74.98 min); (**F15**) HPLC-negative ion ESI-MS analysis (*m/z* 300.0−400.0; t_R = 51.45 and 56.09 min); (**F16**) HPLC-negative ion ESI-MS analysis (*m/z* 300.0−500.0; t_R = 15.25 and 23.03 min); (**F17**) HPLC-negative ion ESI-MS analysis (*m/z* 500.0−700.0; t_R = 71.67 and 78.44 min).

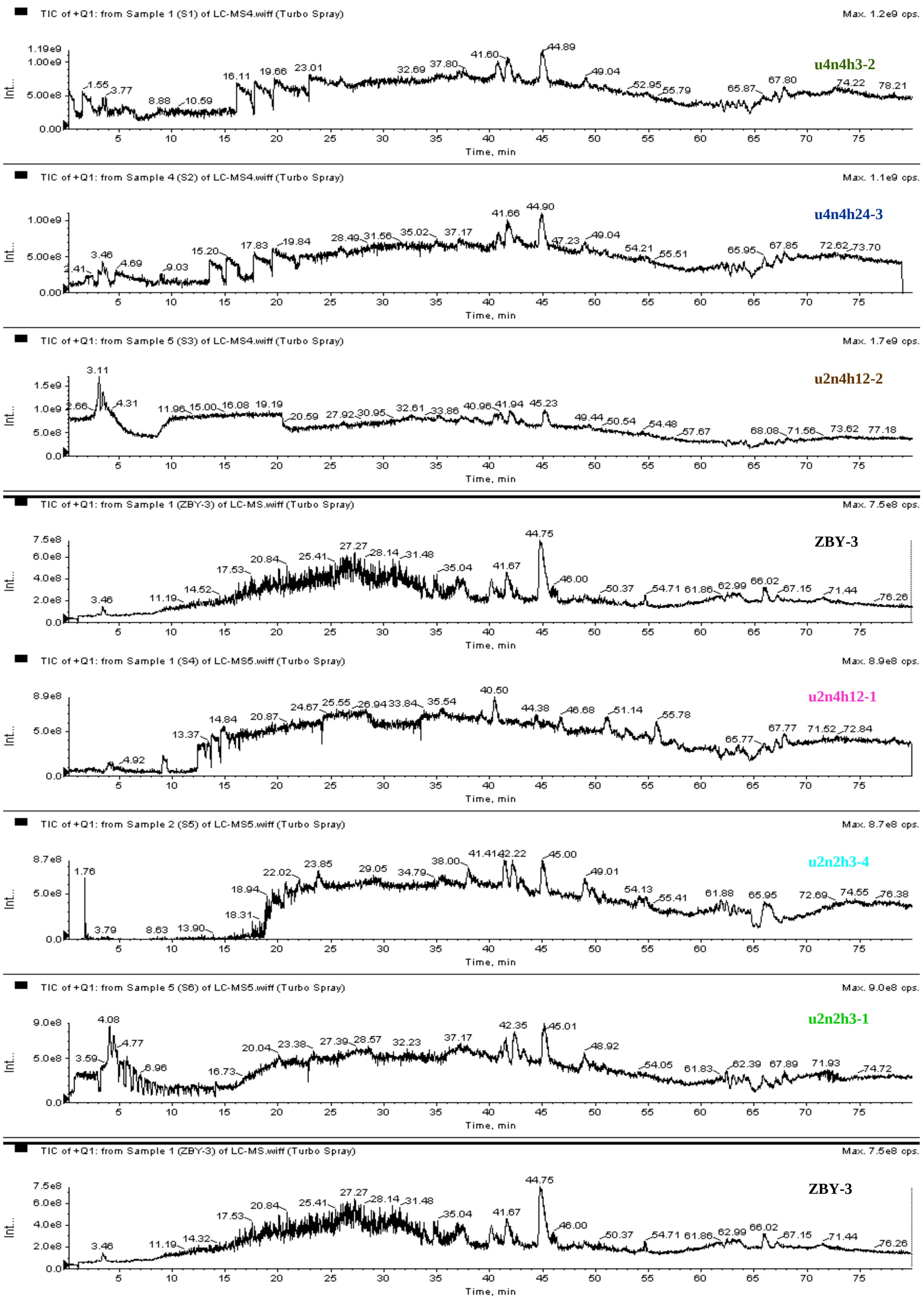
TIC of +Q1: from Sample 1 (S1) of LC-MS4.wiff (Turbo Spray)
Max. 1.2e9 cps.
u4n4h3-2
TIC of +Q1: from Sample 4 (S2) of LC-MS4.wiff (Turbo Spray)
Max. 1.1e9 cps.
u4n4h24-3
TIC of +Q1: from Sample 5 (S3) of LC-MS4.wiff (Turbo Spray)
Max. 1.7e9 cps.
u2n4h12-2
TIC of +Q1: from Sample 1 (ZBY-3) of LC-MS.wiff (Turbo Spray)
Max. 7.5e8 cps.
ZBY-3
TIC of +Q1: from Sample 1 (S4) of LC-MS5.wiff (Turbo Spray)
Max. 8.9e8 cps.
u2n4h12-1
TIC of +Q1: from Sample 2 (S5) of LC-MS5.wiff (Turbo Spray)
Max. 8.7e8 cps.
u2n2h3-4
TIC of +Q1: from Sample 5 (S6) of LC-MS5.wiff (Turbo Spray)
Max. 9.0e8 cps.
u2n2h3-1
TIC of +Q1: from Sample 1 (ZBY-3) of LC-MS.wiff (Turbo Spray)
Max. 7.5e8 cps.
ZBY-3
Int...
Time, min

(A)

Figure S2. *Cont.*

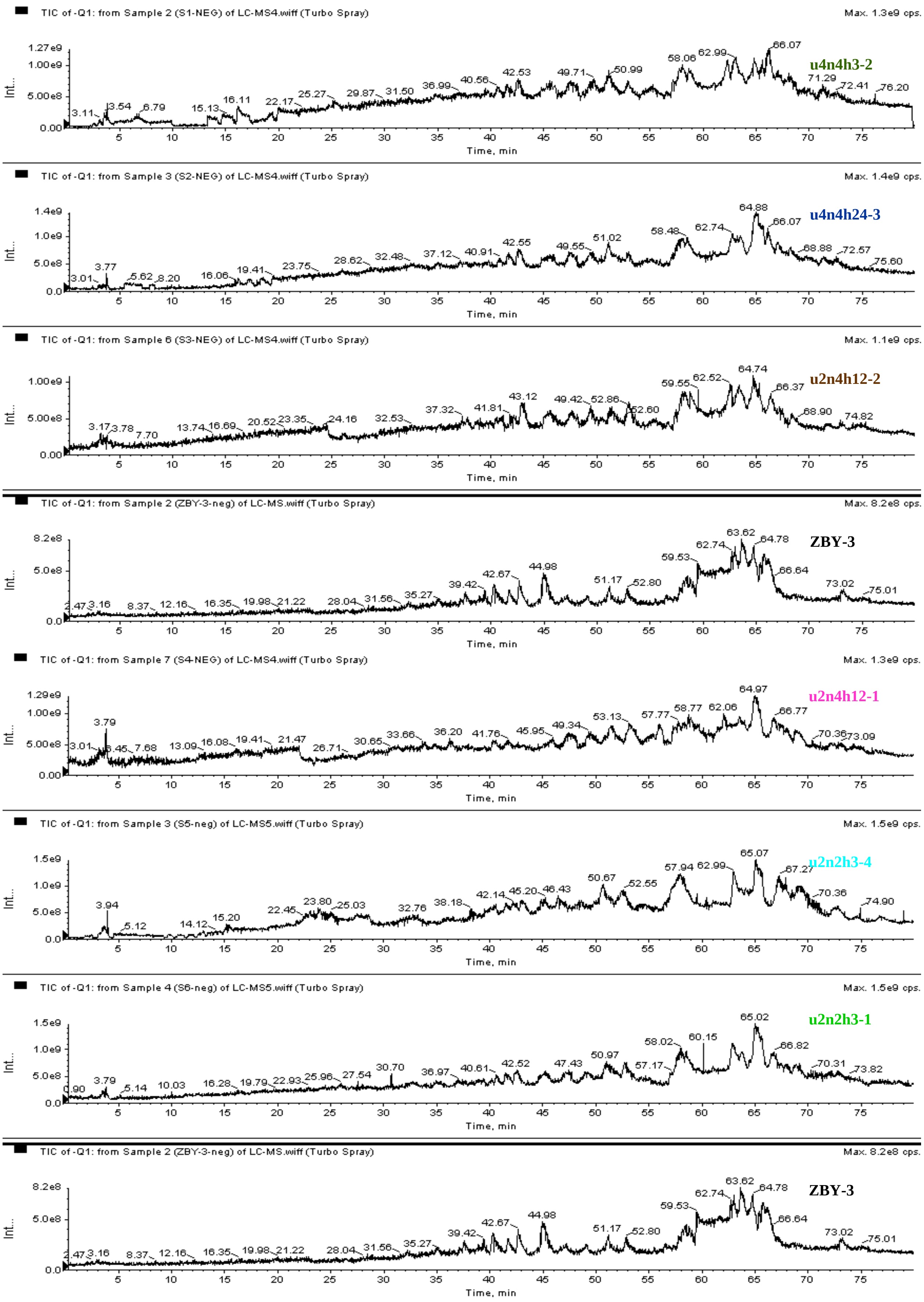

(B1)

Figure S2. *Cont.*

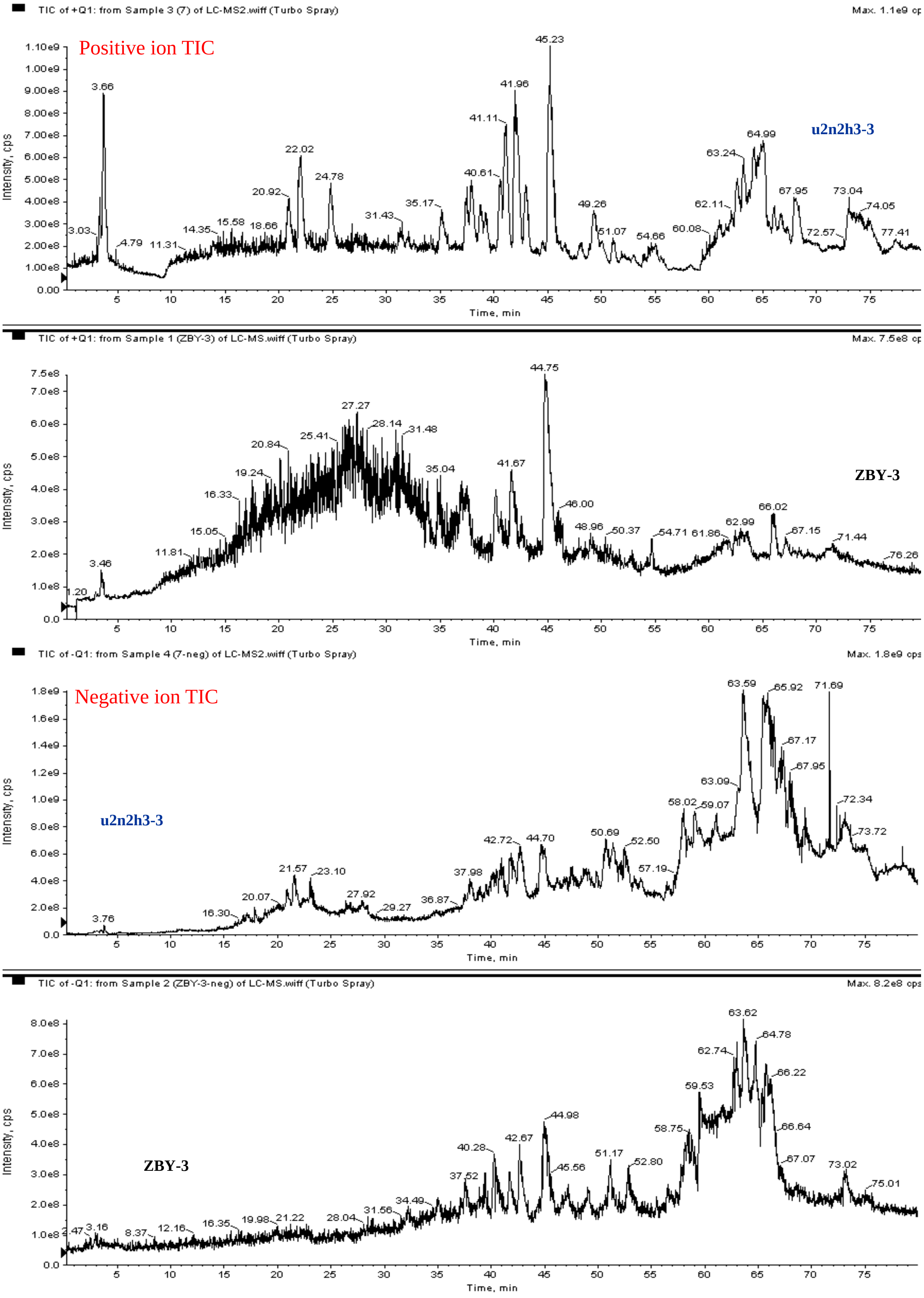

(B2)

Figure S2. *Cont.*

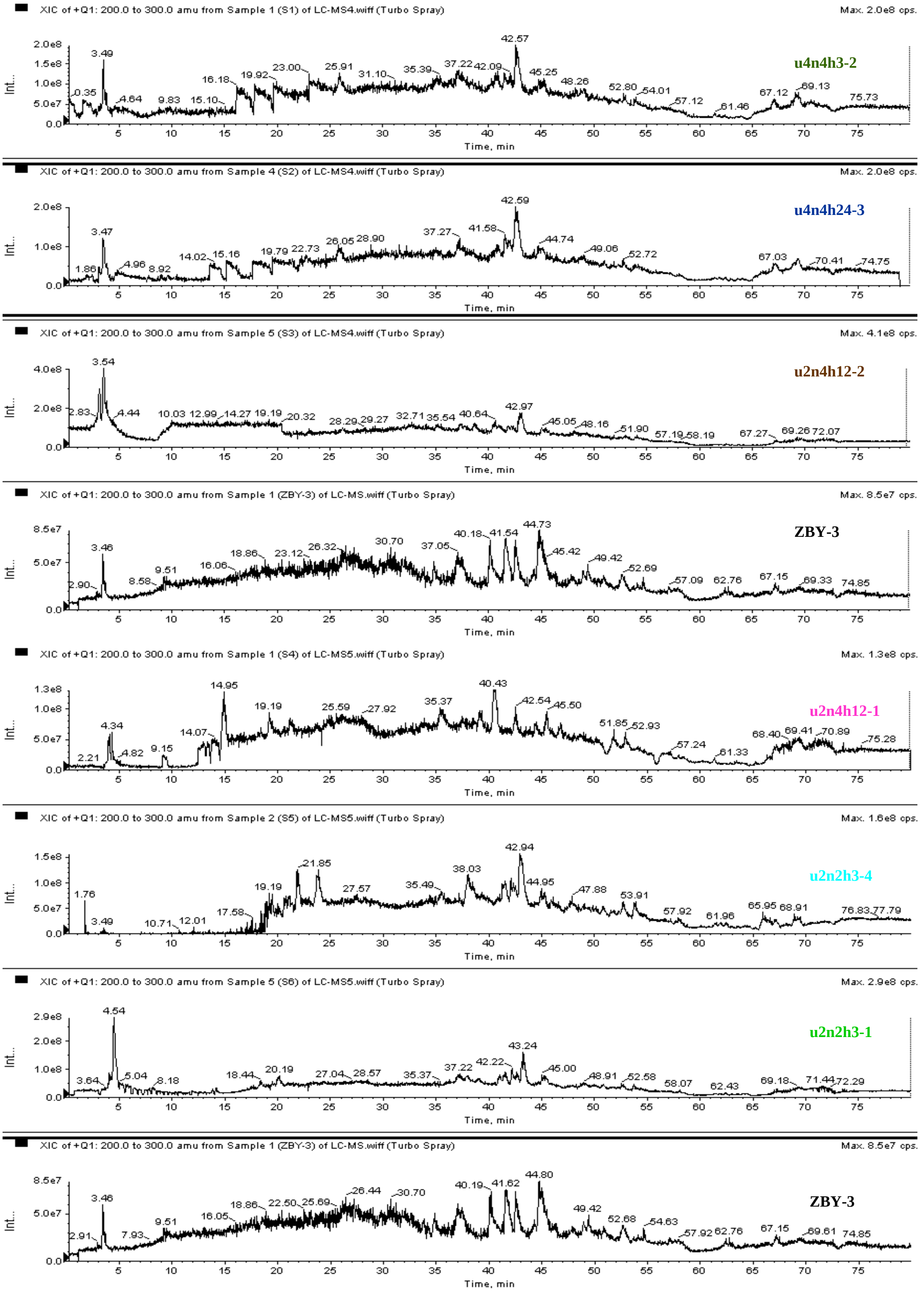

(C1)

Figure S2. *Cont.*

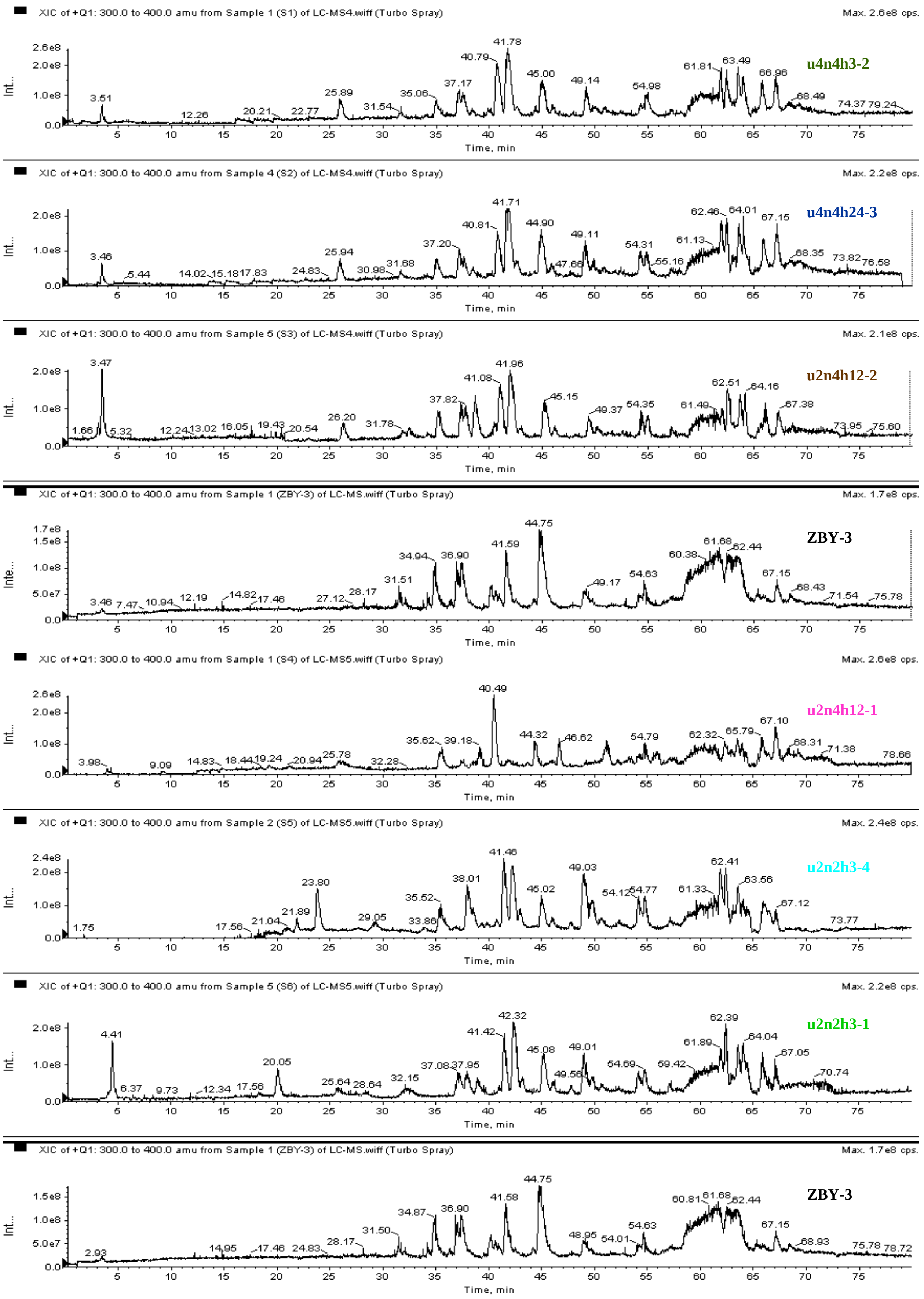

(C2)

Figure S2. *Cont.*

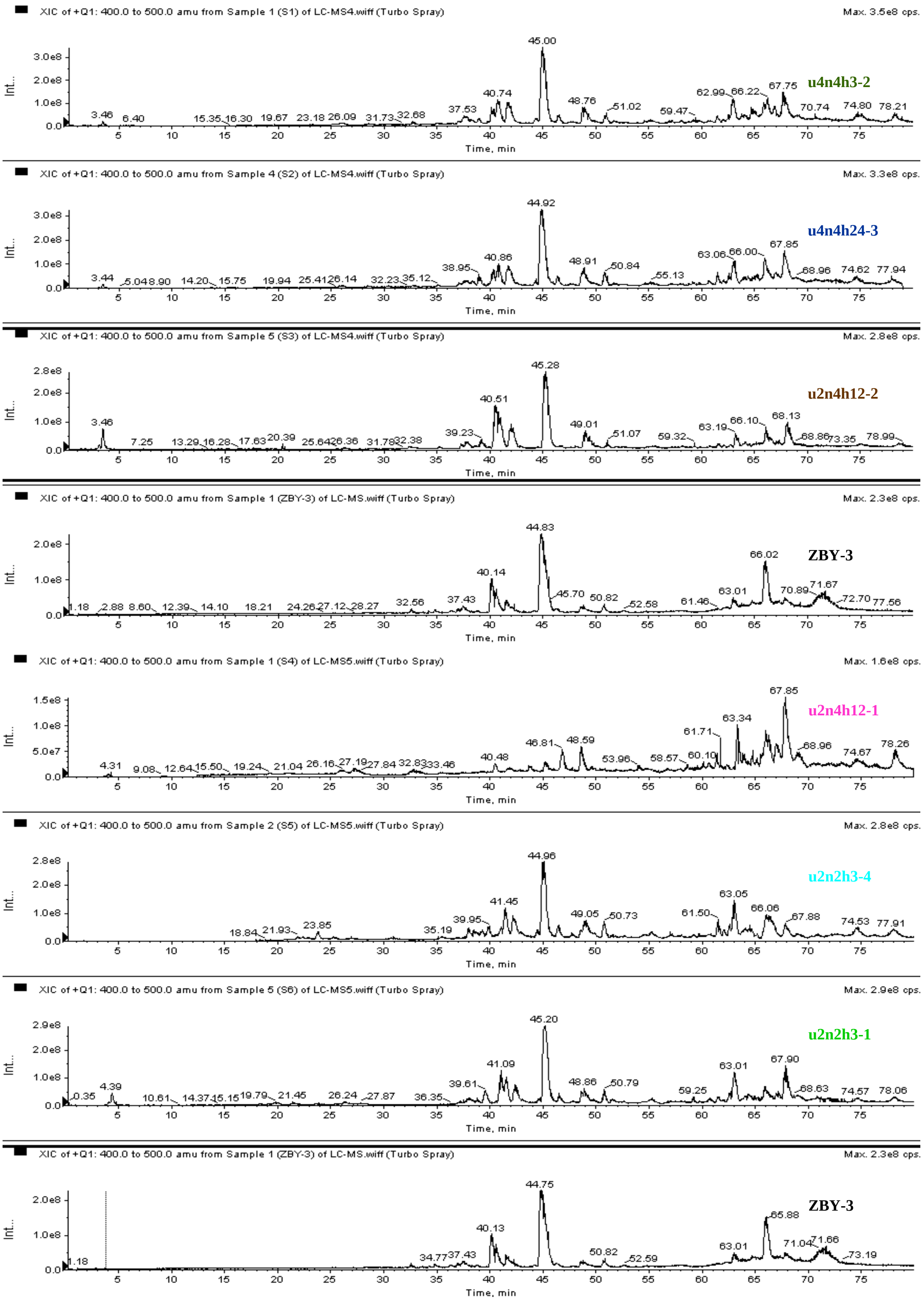

(C3)

Figure S2. *Cont.*

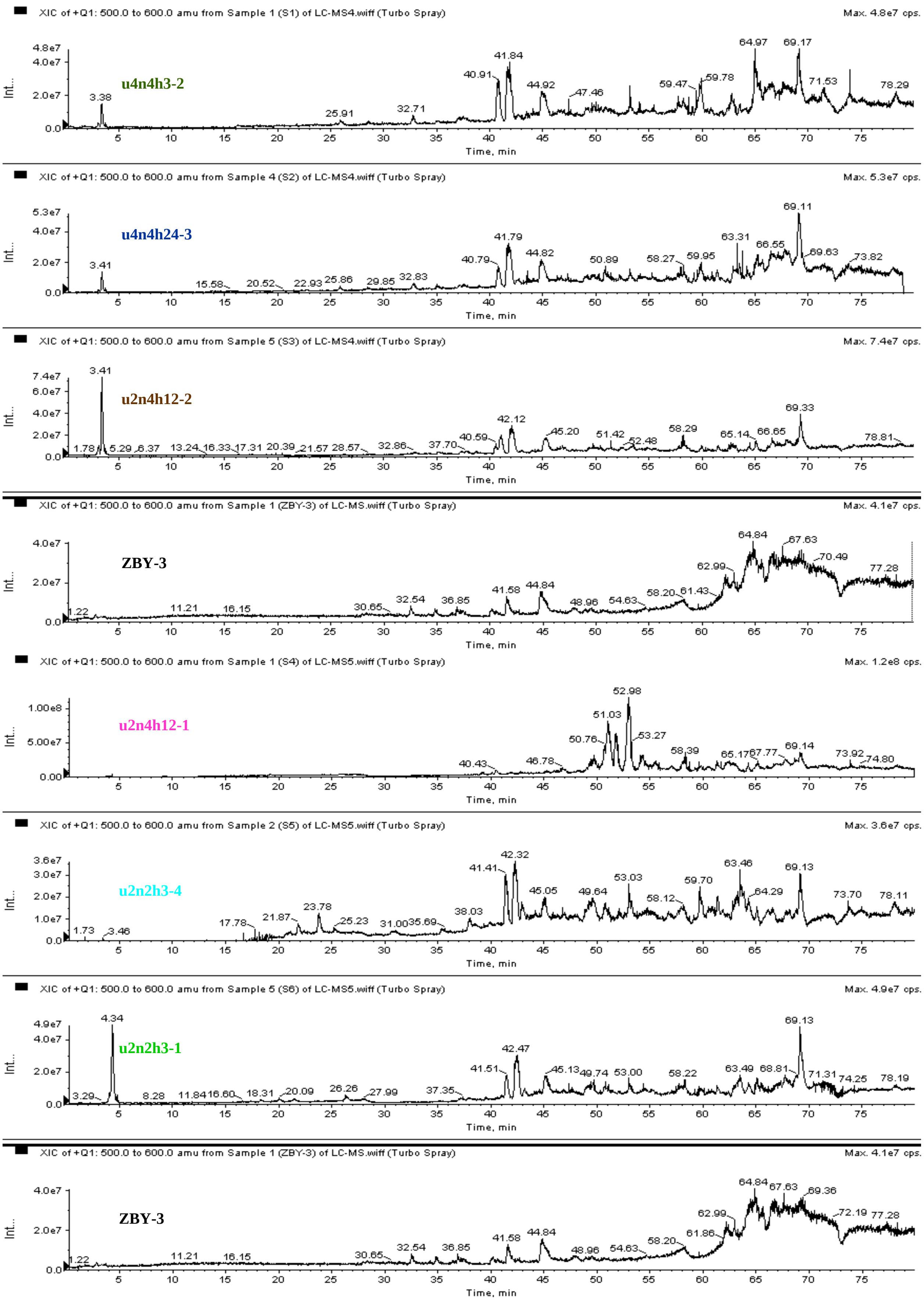

(C4)

Figure S2. *Cont.*

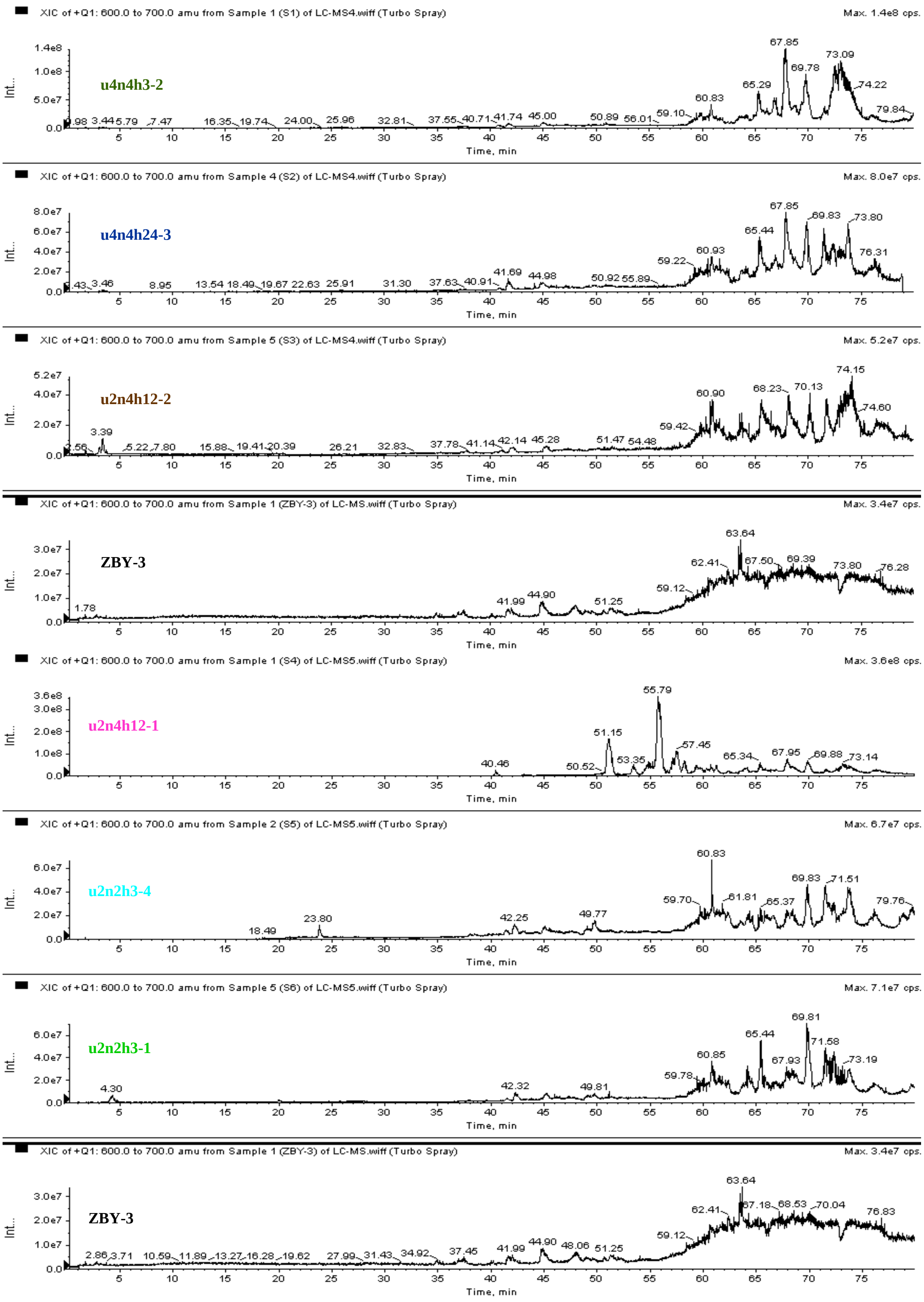

(C5)

Figure S2. *Cont.*

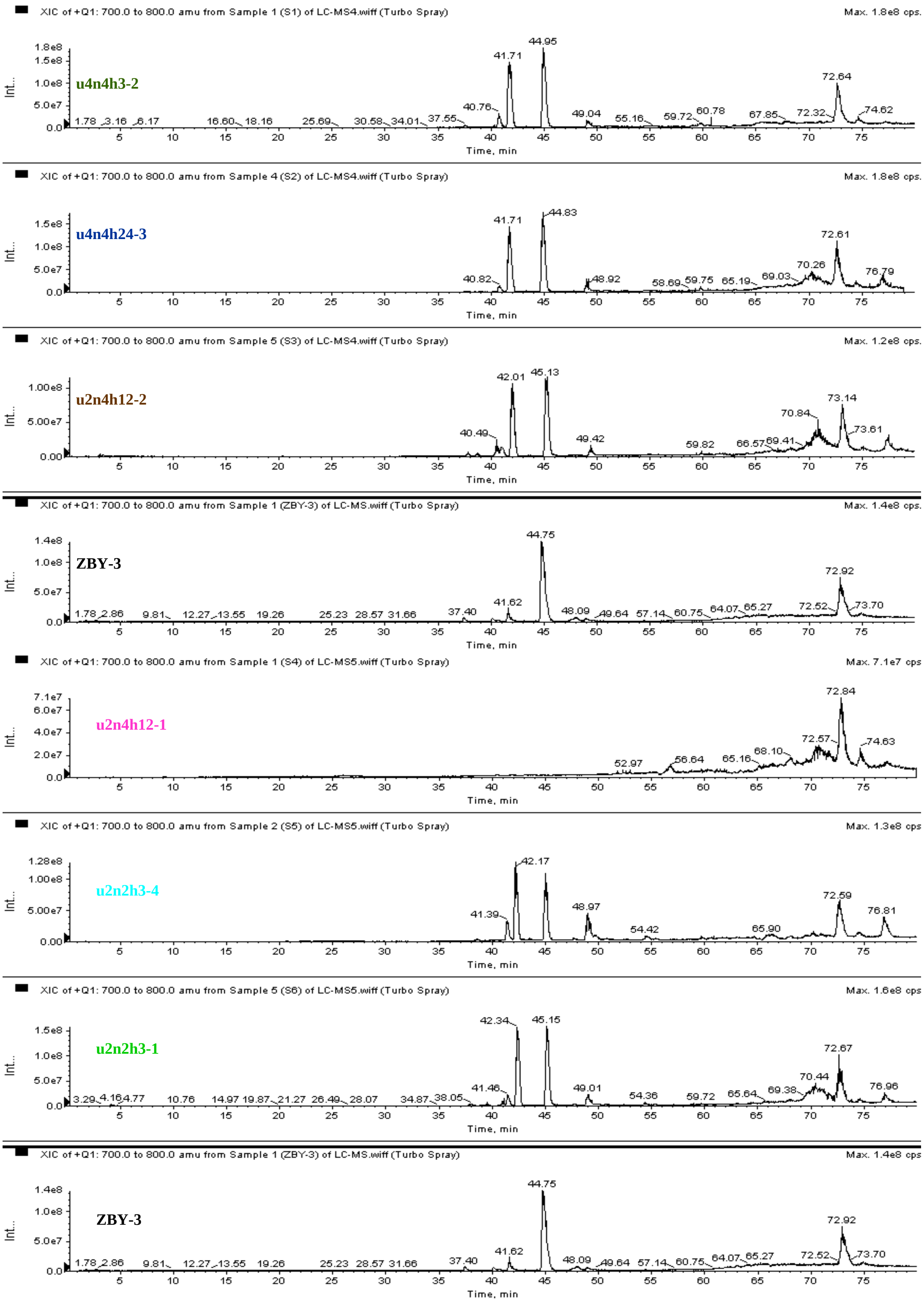

(C6)

Figure S2. *Cont.*

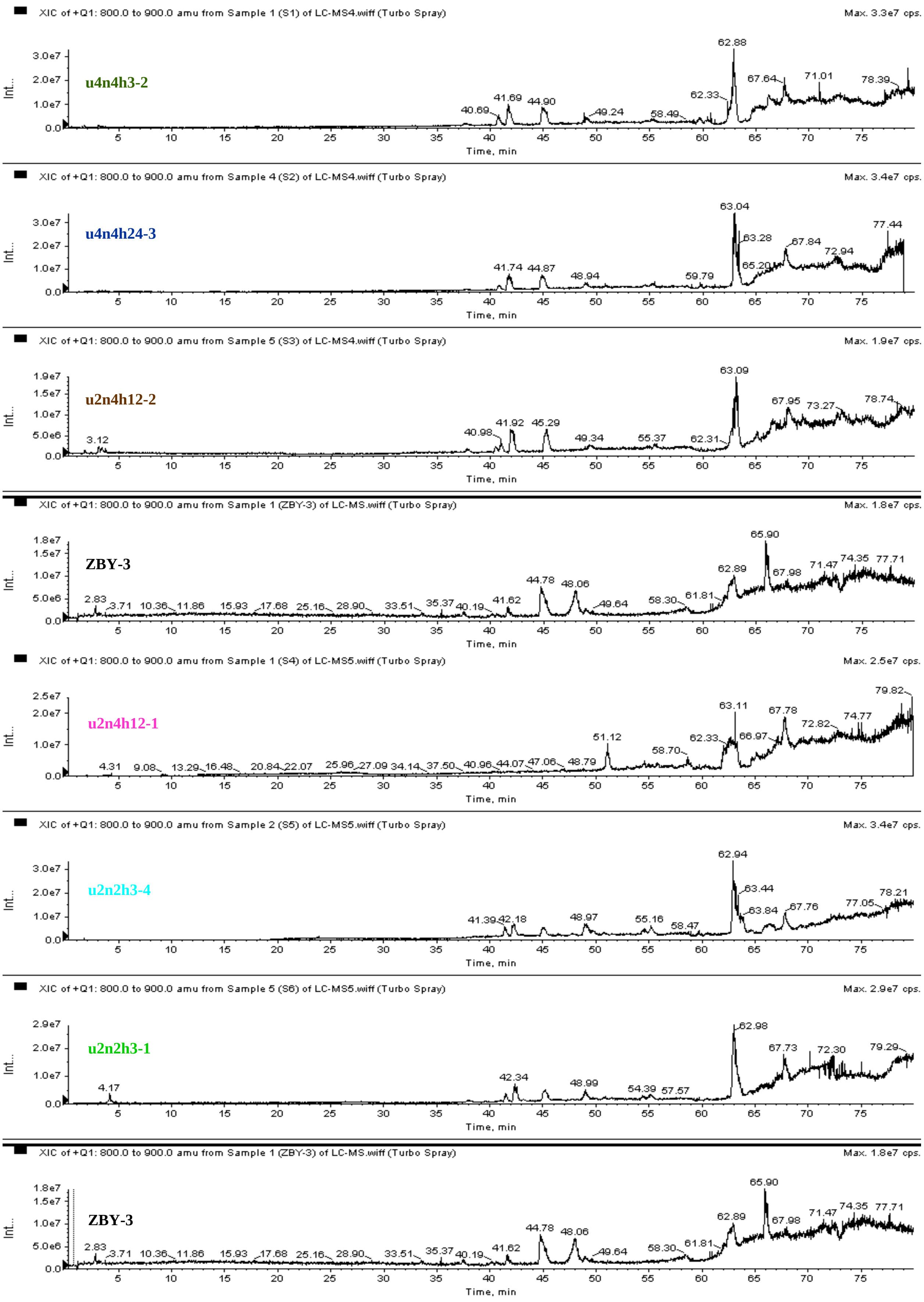

(C7)

Figure S2. *Cont.*

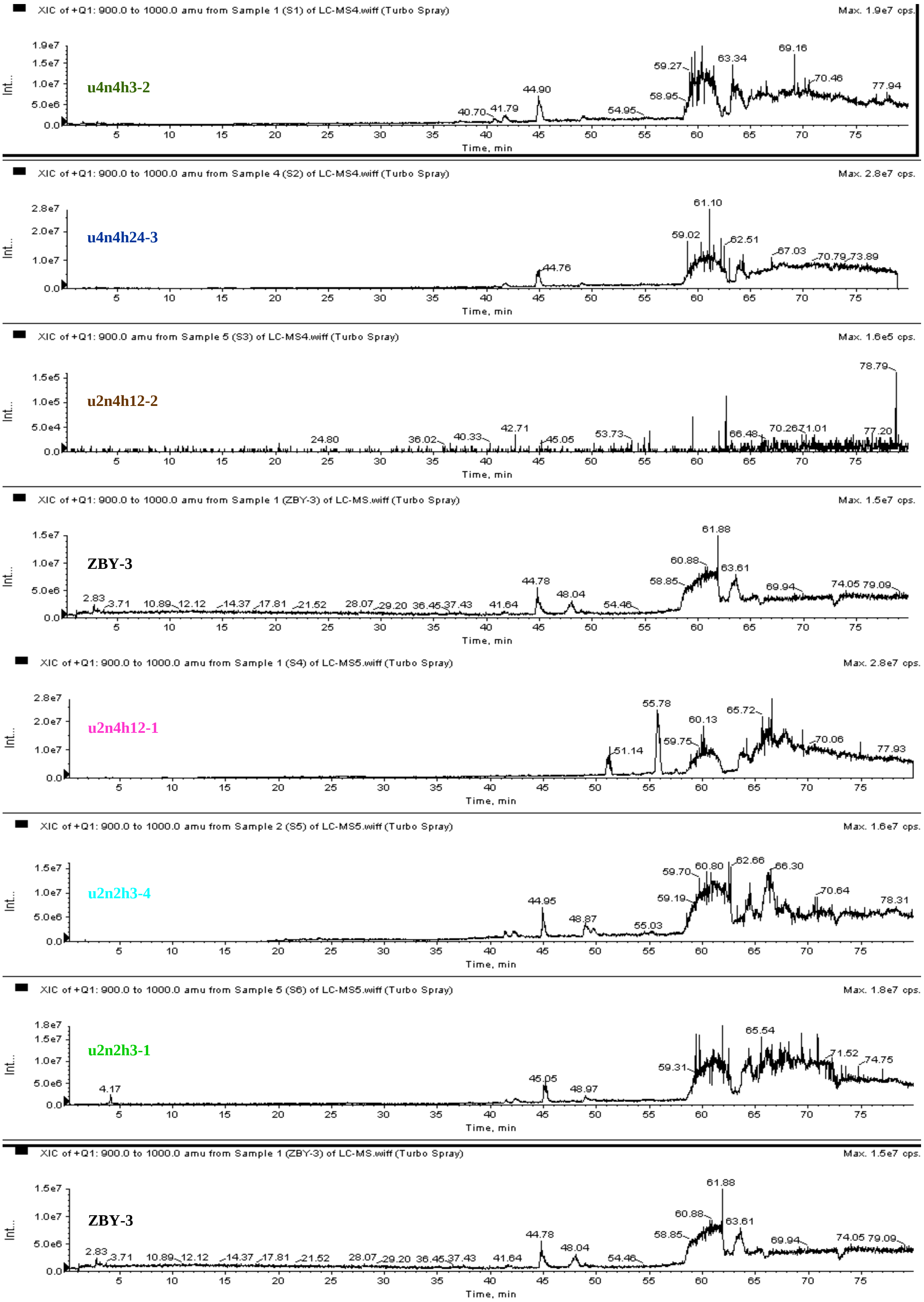

(C8)

Figure S2. *Cont.*

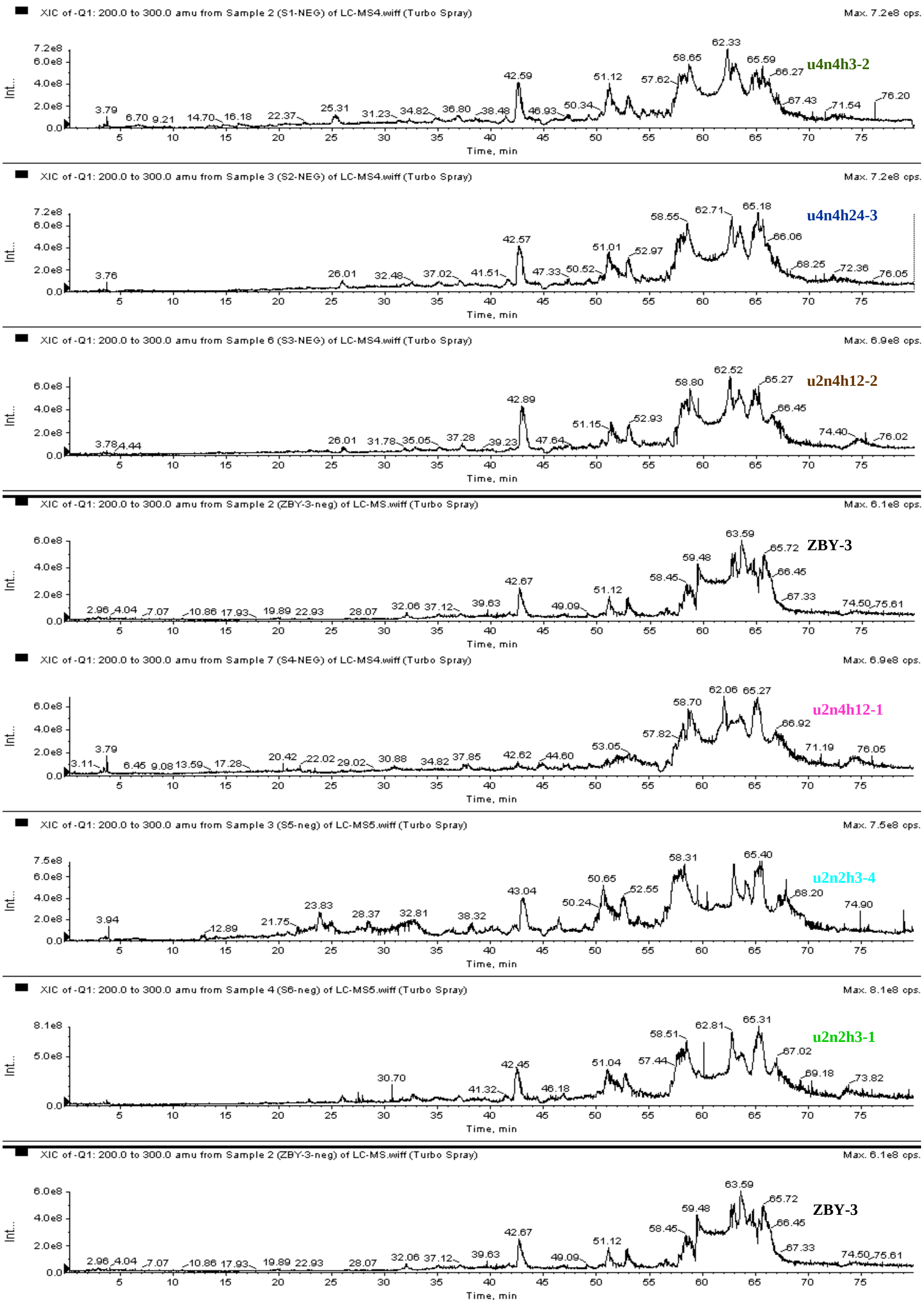

(D1)

Figure S2. *Cont.*

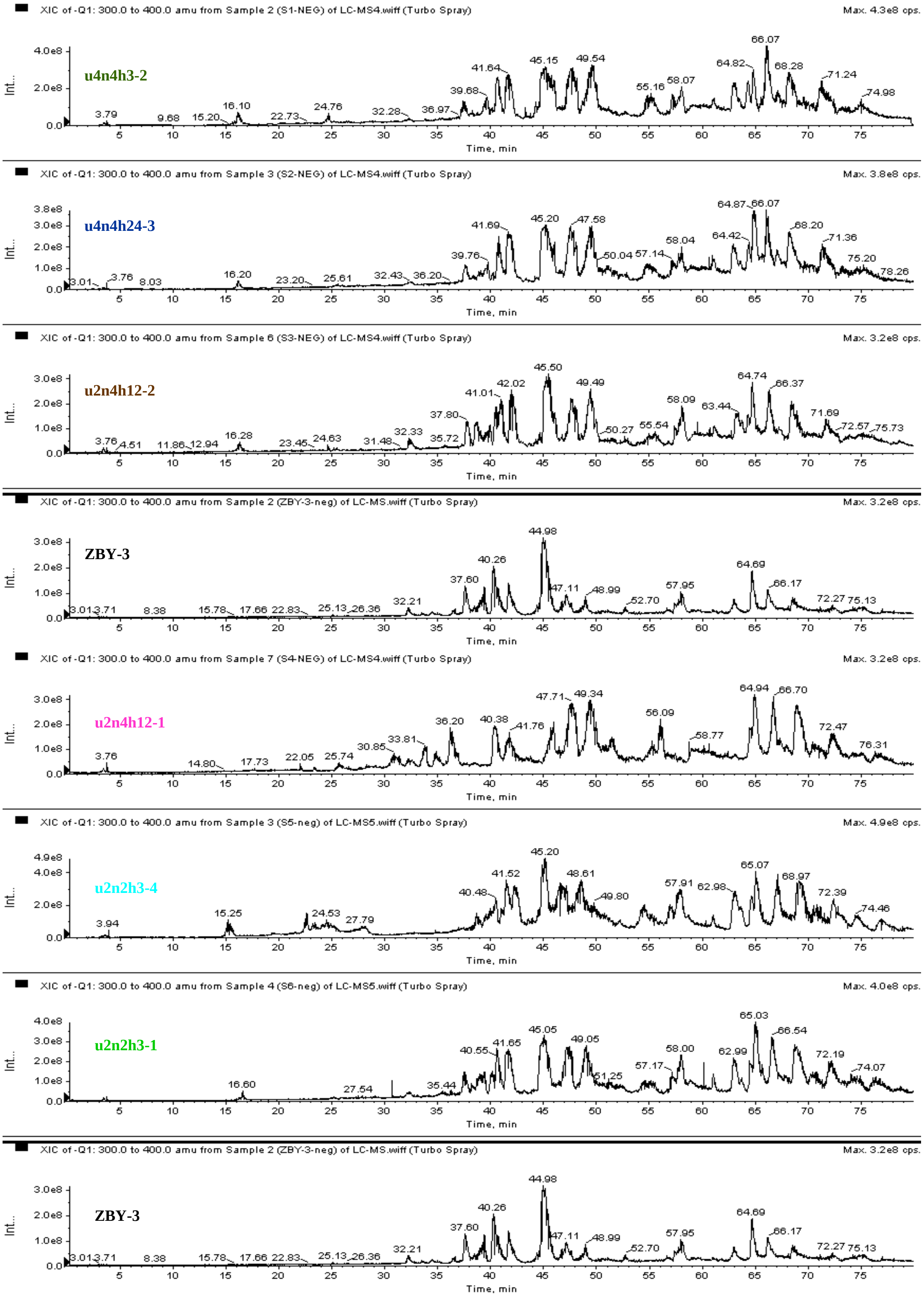

(D2)

Figure S2. *Cont.*

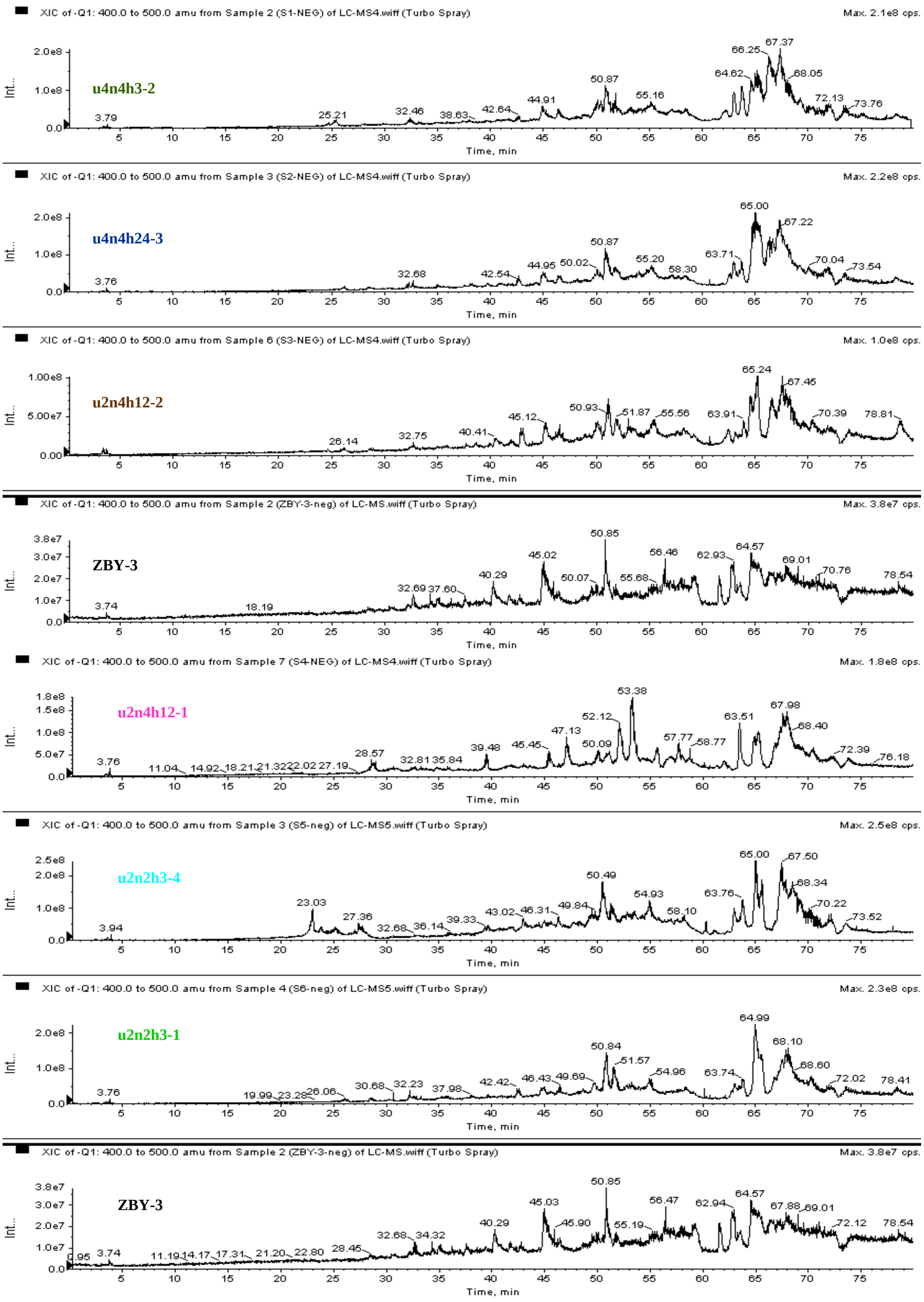

(D3)

Figure S2. *Cont.*

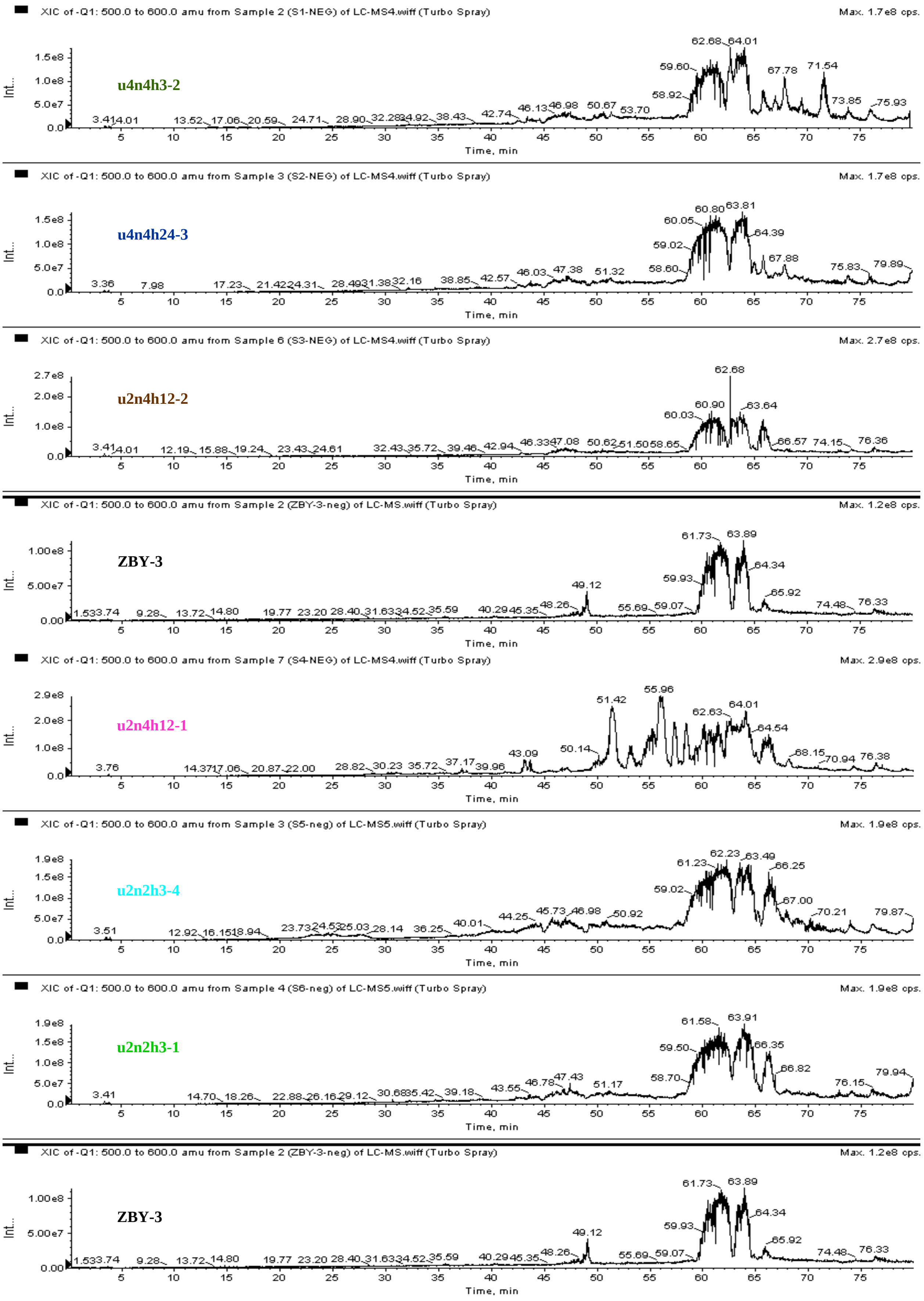

(D4)

Figure S2. *Cont.*

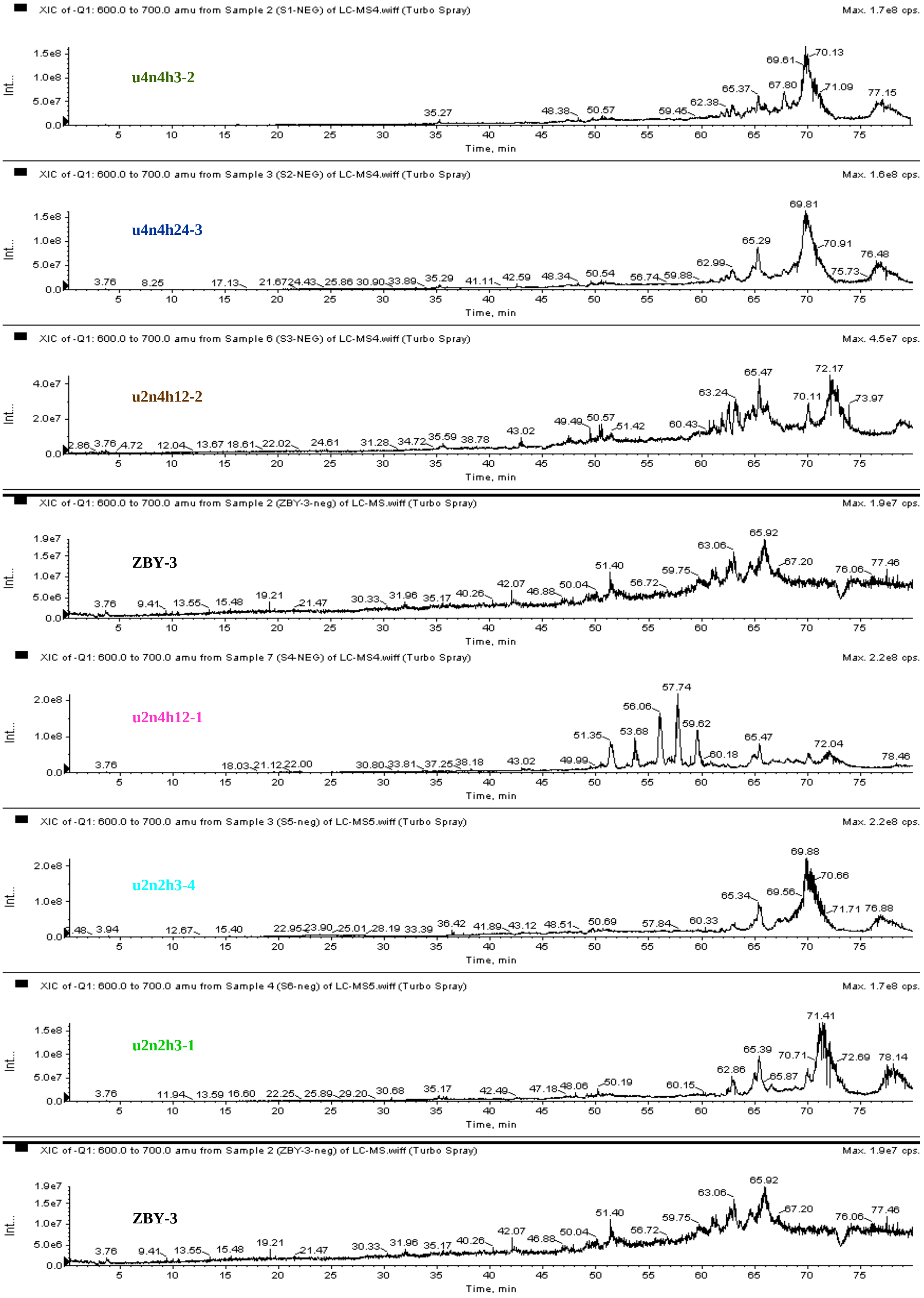

(D5)

Figure S2. *Cont.*

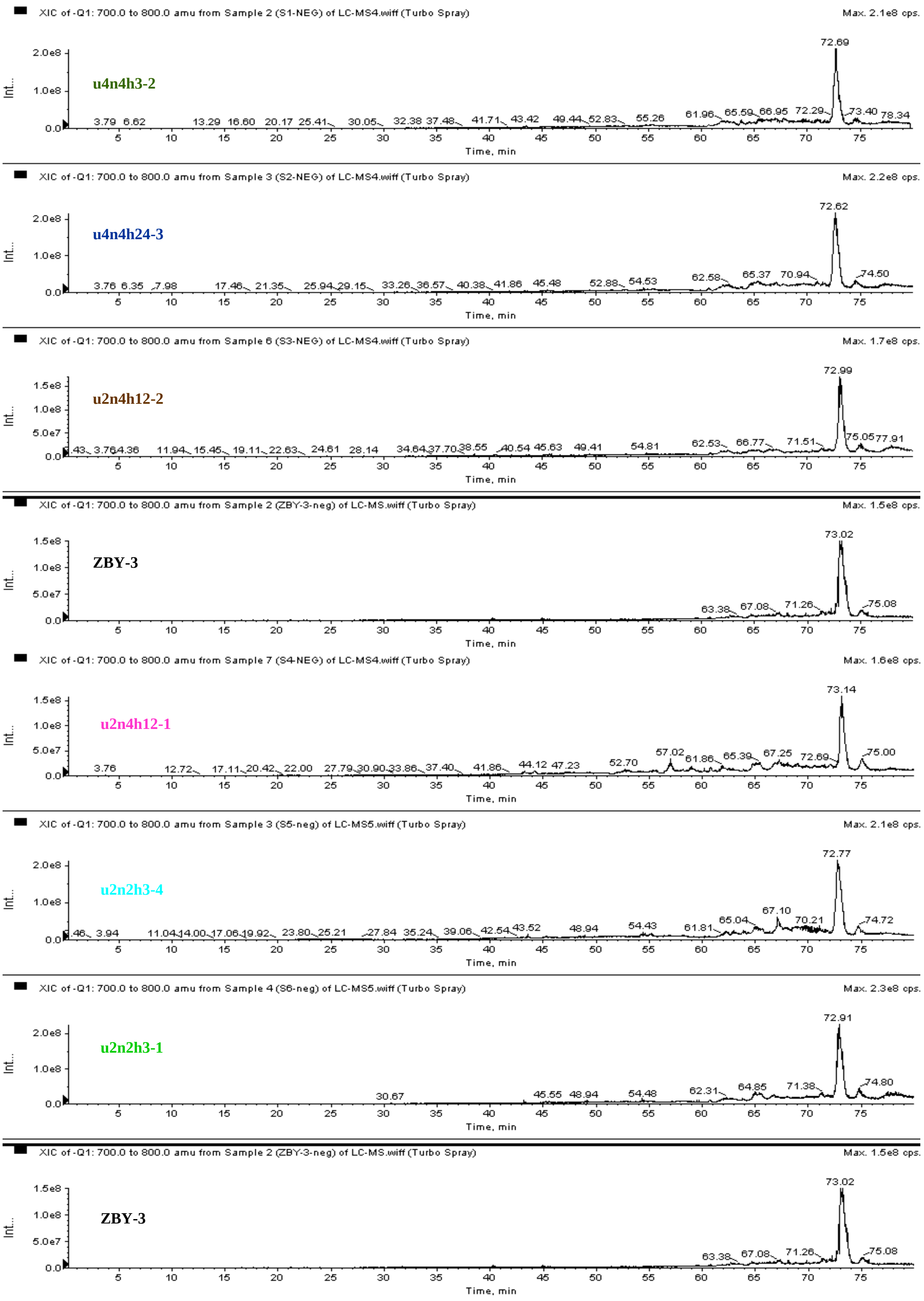

(D6)

Figure S2. *Cont.*

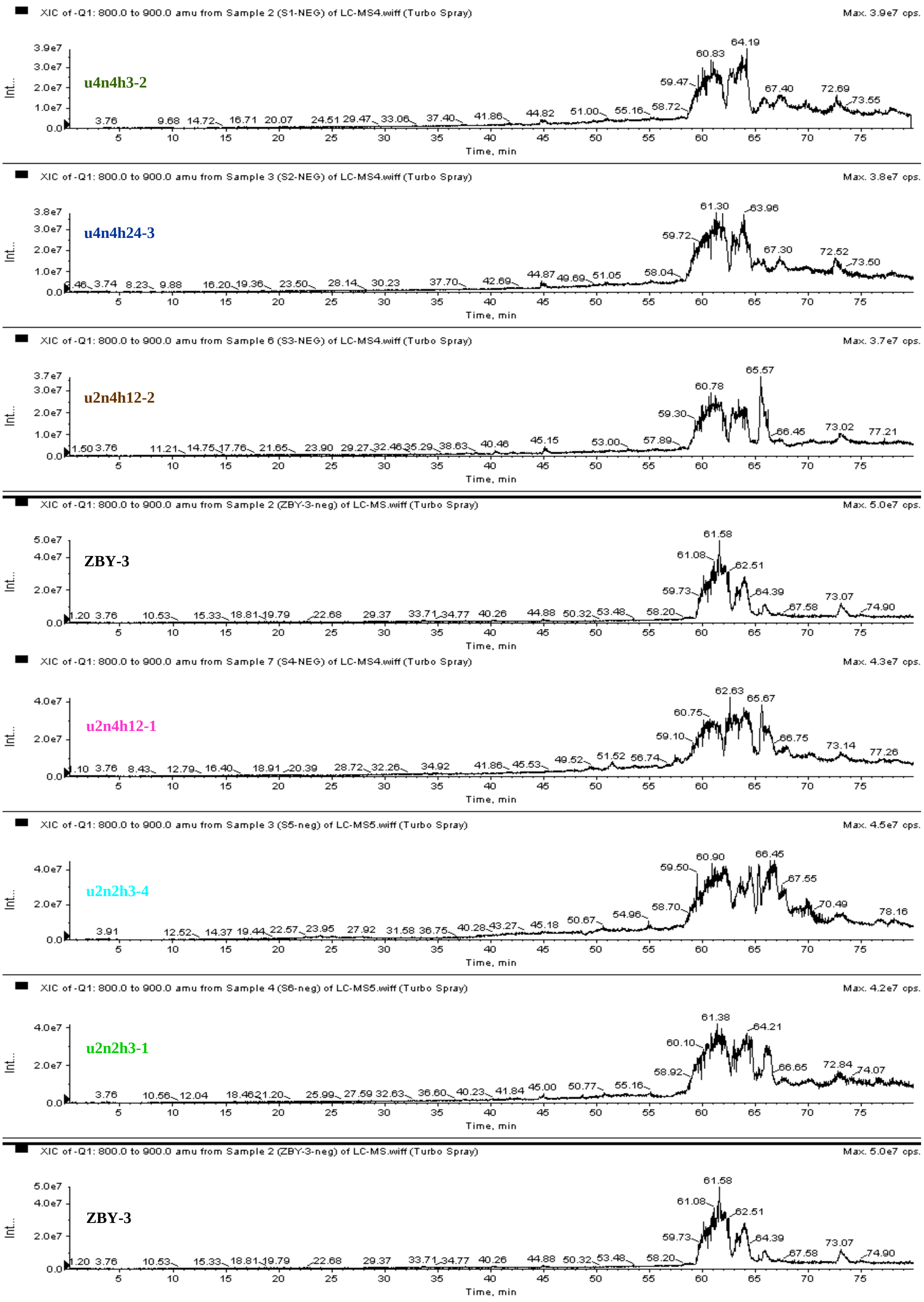

(D7)

Figure S2. *Cont.*

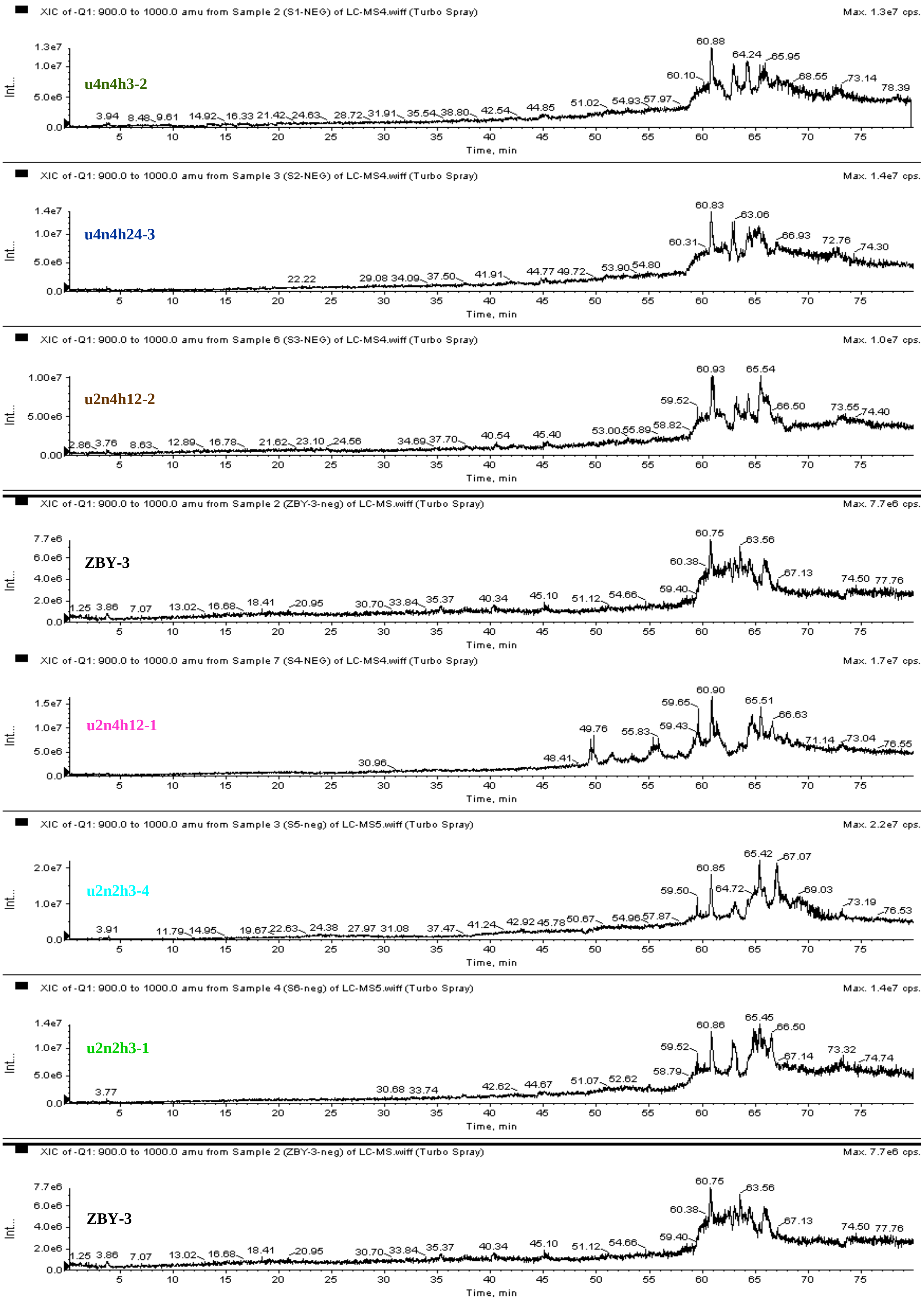

(D8)

Figure S2. *Cont.*

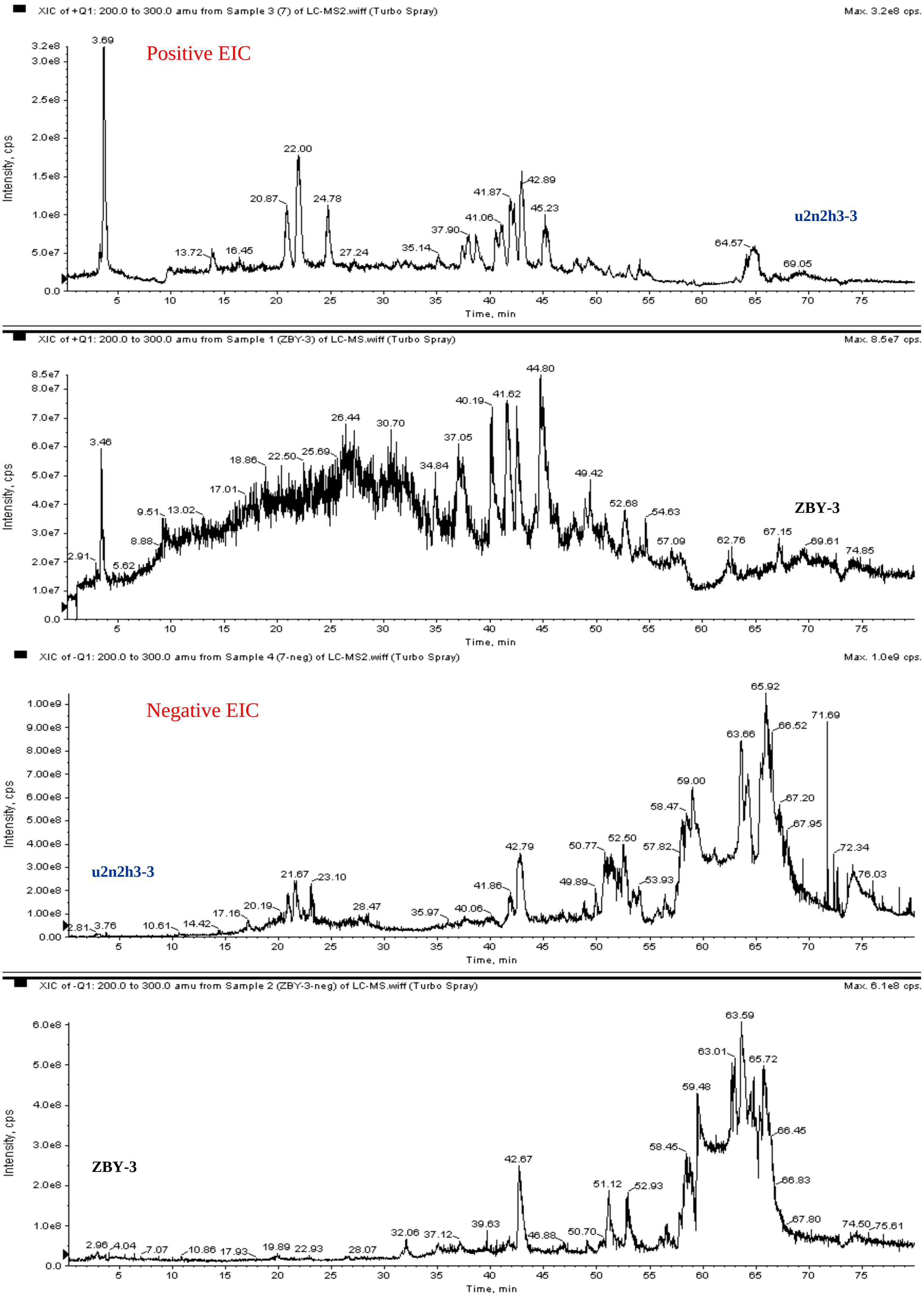

(E1)

Figure S2. *Cont.*

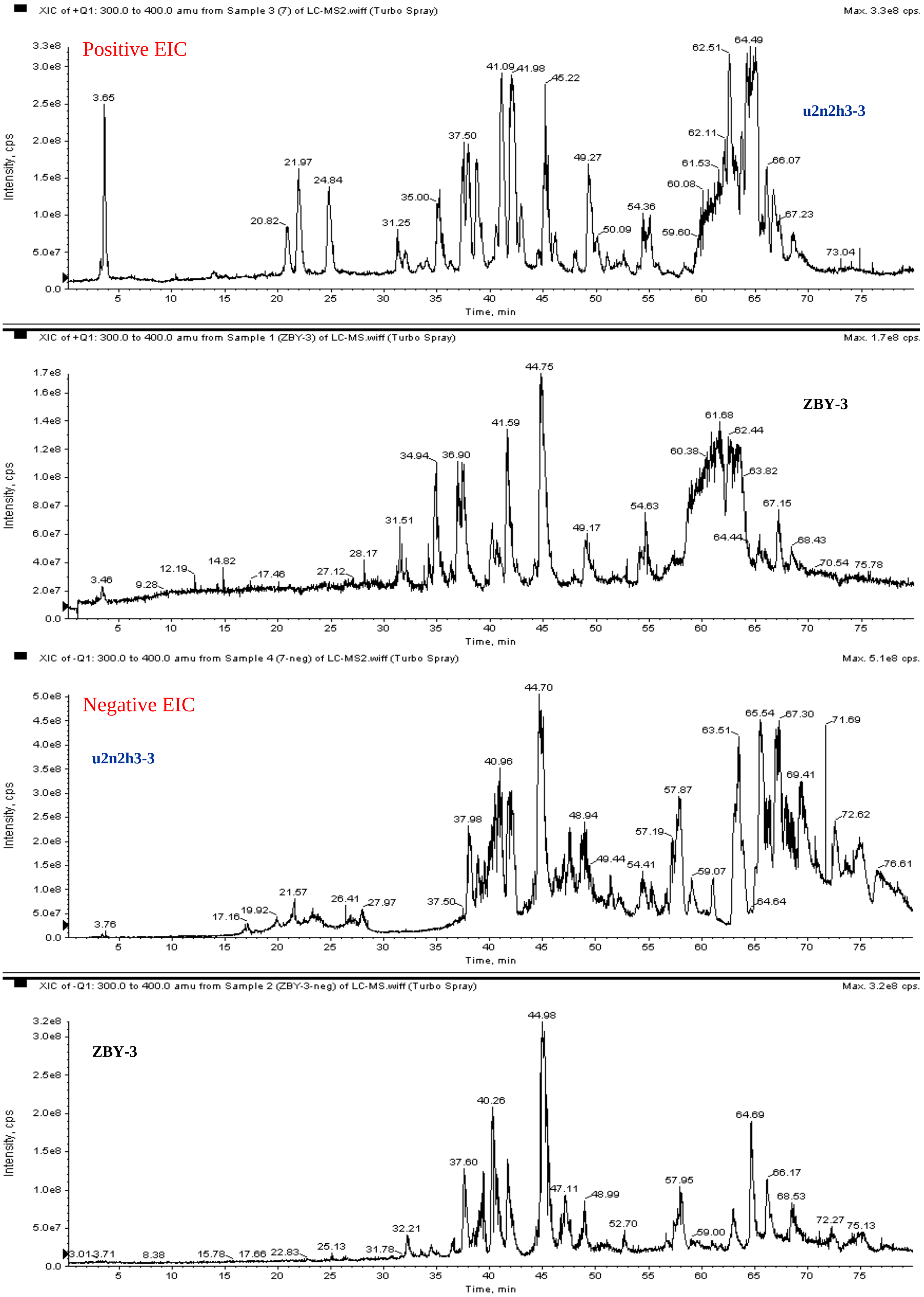

(E2)

Figure S2. *Cont.*

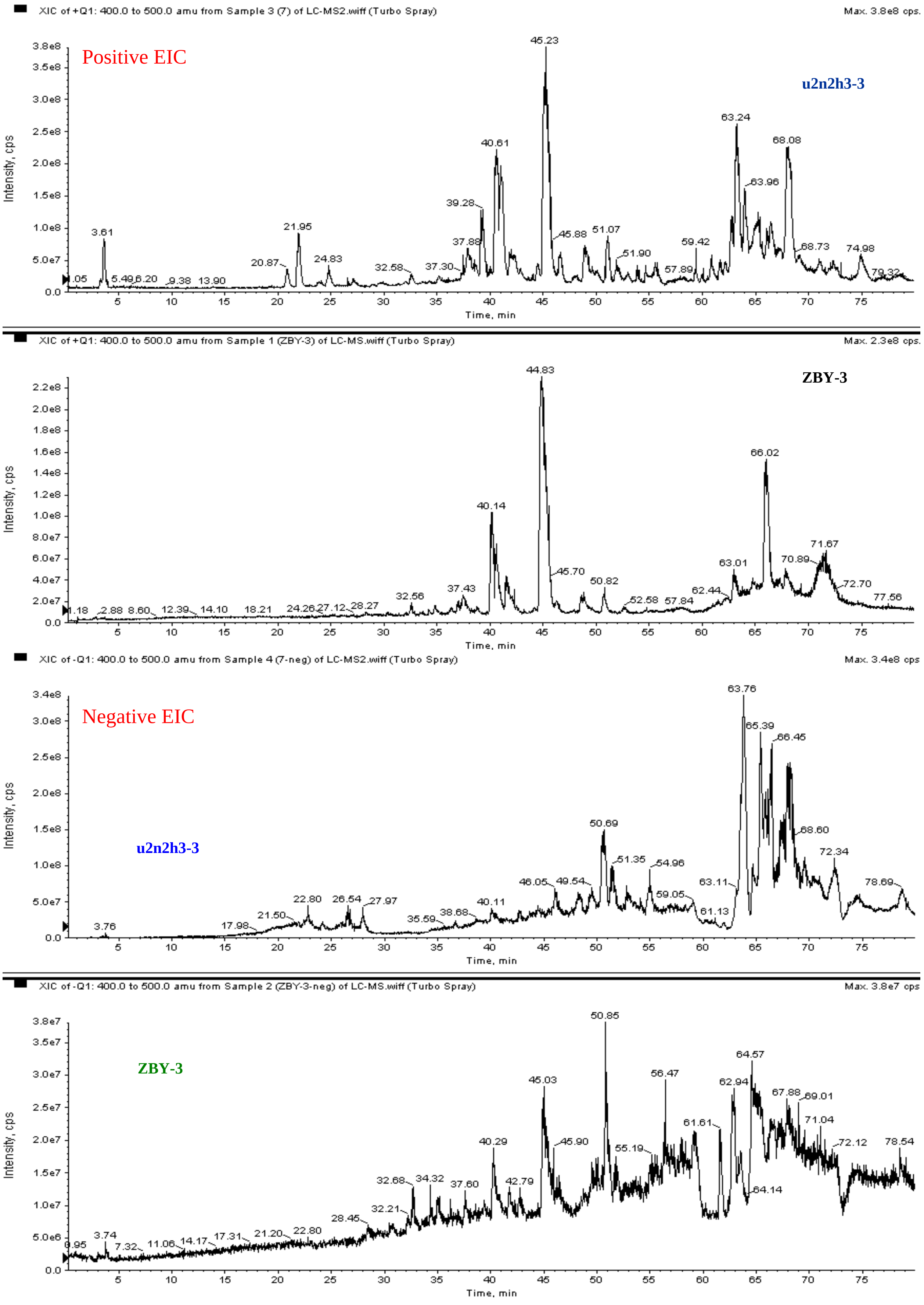

(E3)

Figure S2. *Cont.*

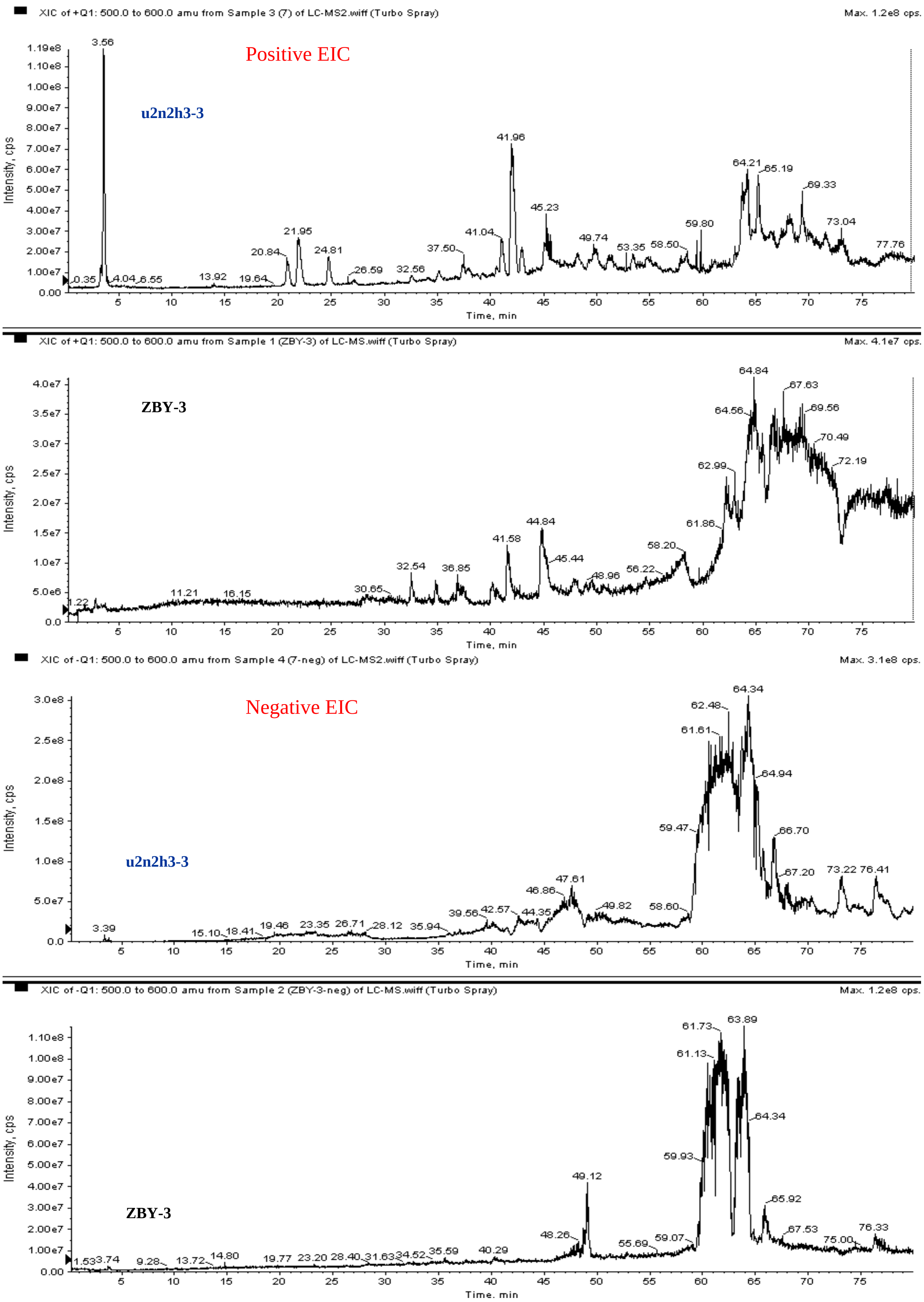

(E4)

Figure S2. *Cont.*

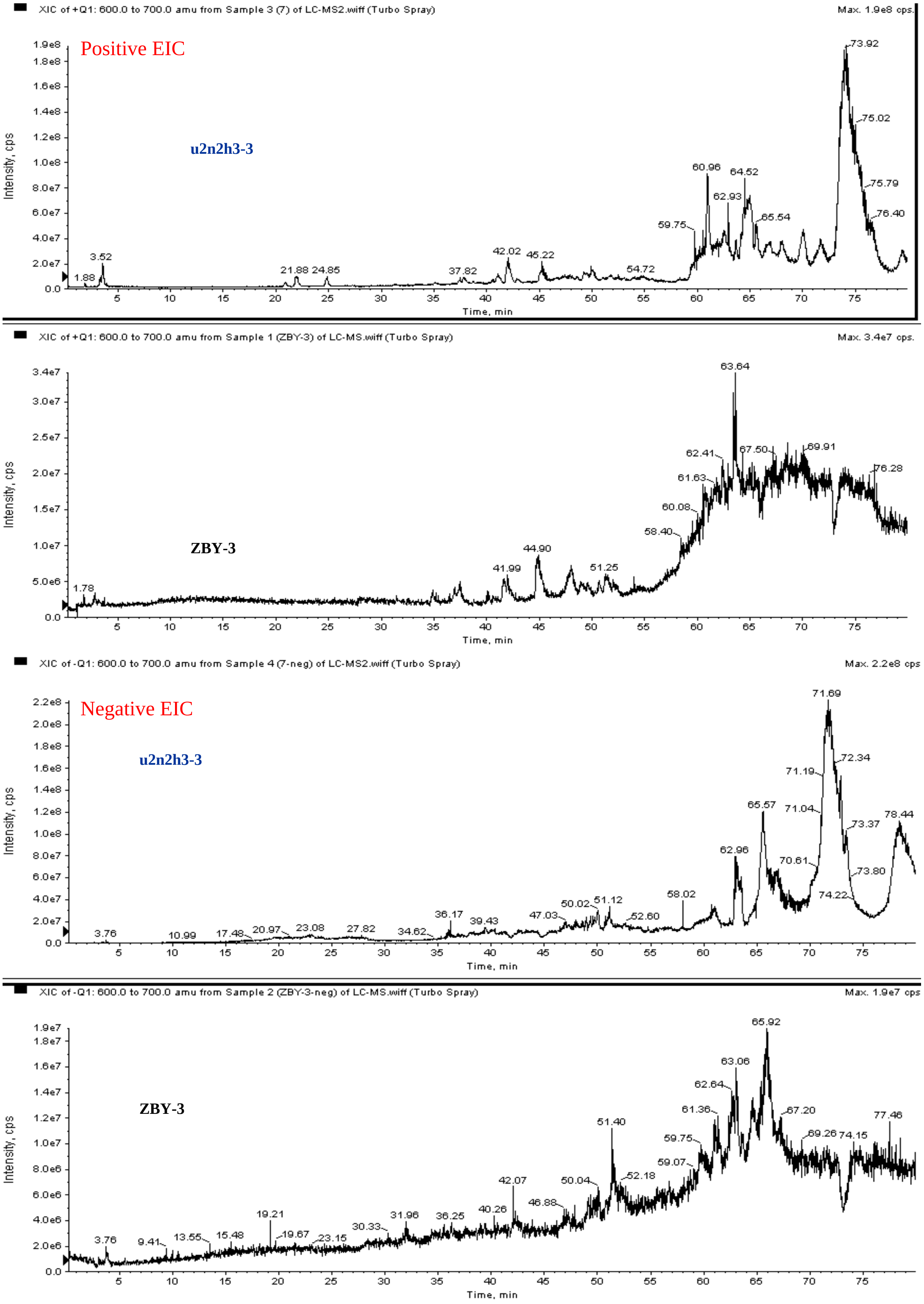

(E5)

Figure S2. *Cont.*

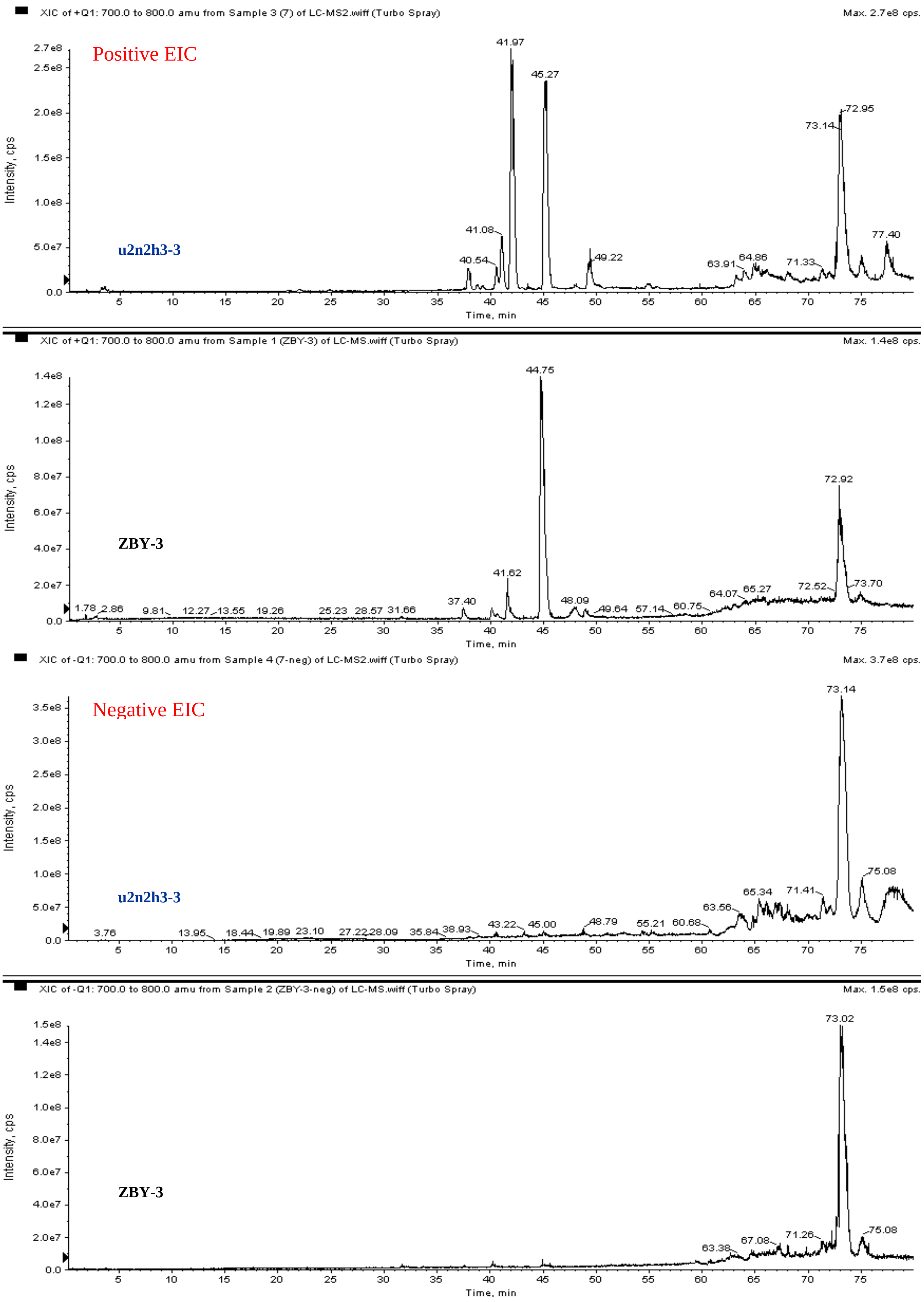

(E6)

Figure S2. *Cont.*

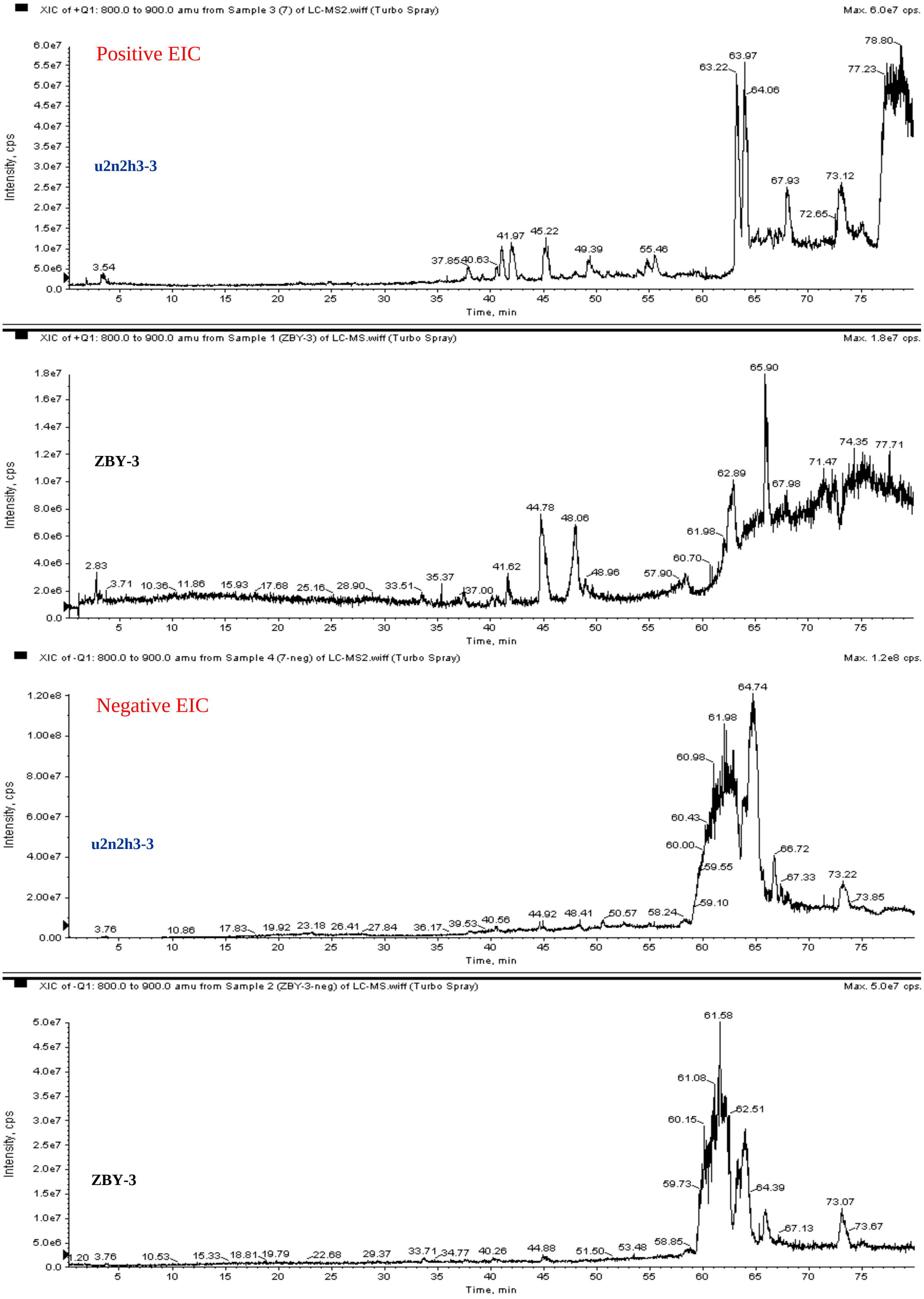

(E7)

Figure S2. *Cont.*

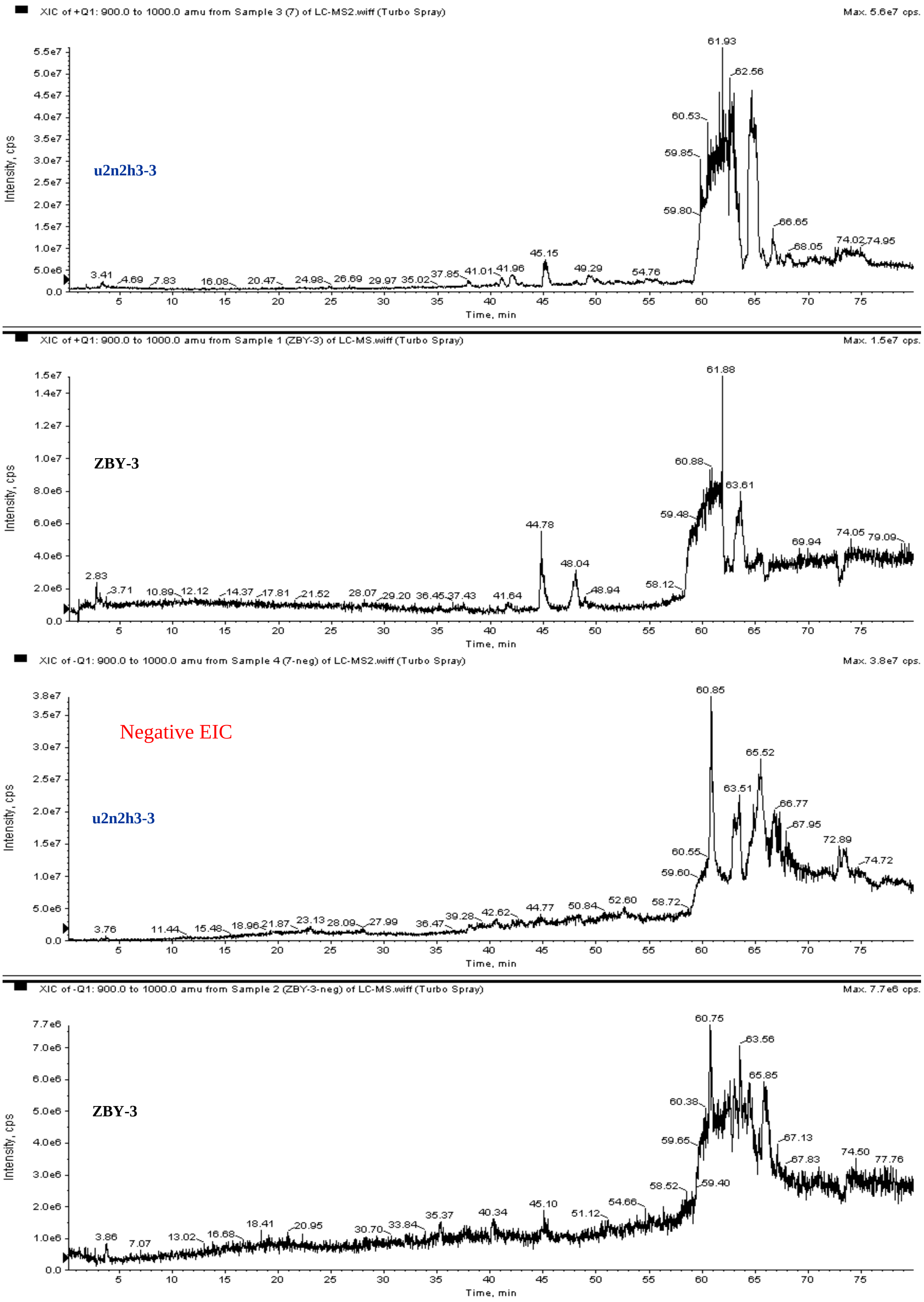

(E8)

Figure S2. *Cont.*

Extracted Positive Ion (*m/z* 300.0-400.0) Chromatograms ESIMS Spectra in the Range of 25.71-26.41 min Retention Times

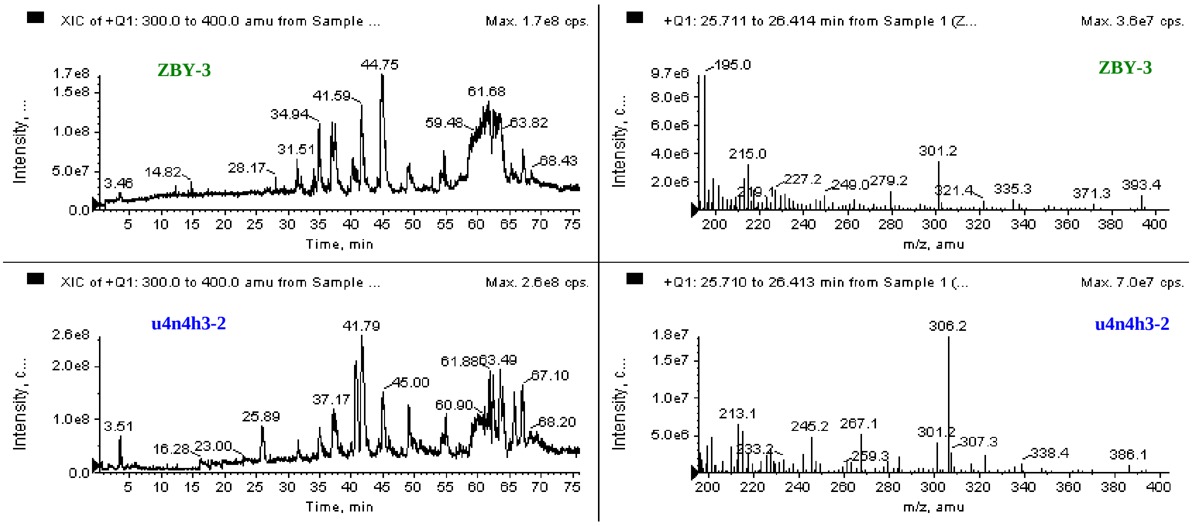

(F1)

Extracted Positive Ion (*m/z* 500.0-600.0) Chromatograms ESIMS Spectra taken at the 69.11 min Retention Time

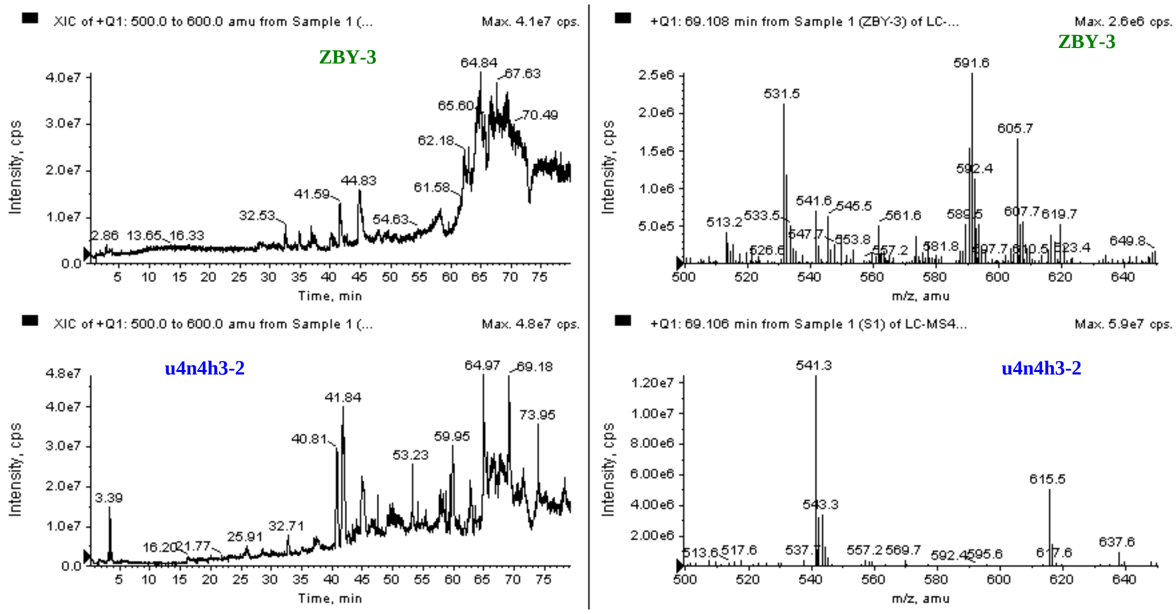

(F2)

Figure S2. *Cont.*

Extracted Positive Ion (*m/z* 700.0-800.0) Chromatograms ESIMS Spectra taken at the 70.19 min Retention Time

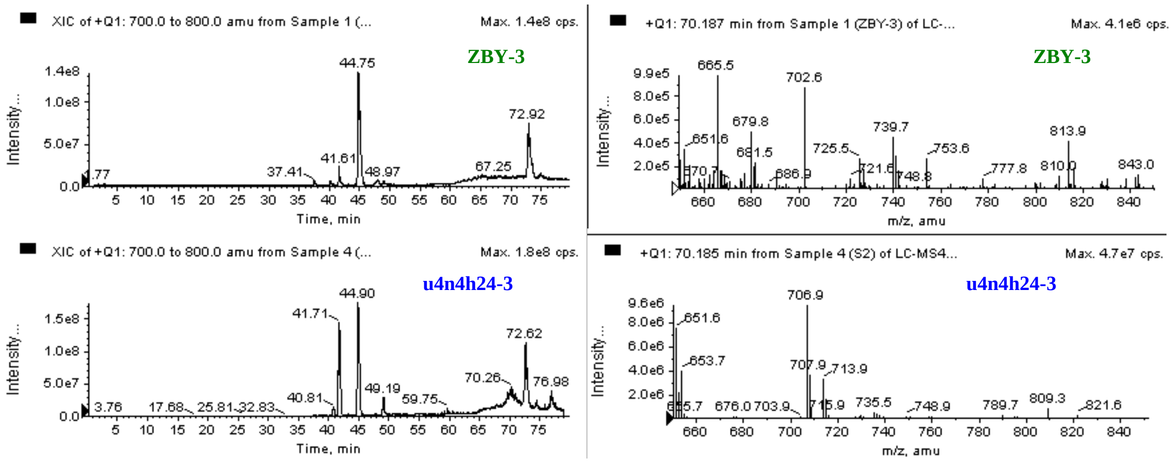

(F3)

Extracted Positive Ion (*m/z* 700.0-800.0) Chromatograms ESIMS Spectra taken at the 76.98 min Retention Time

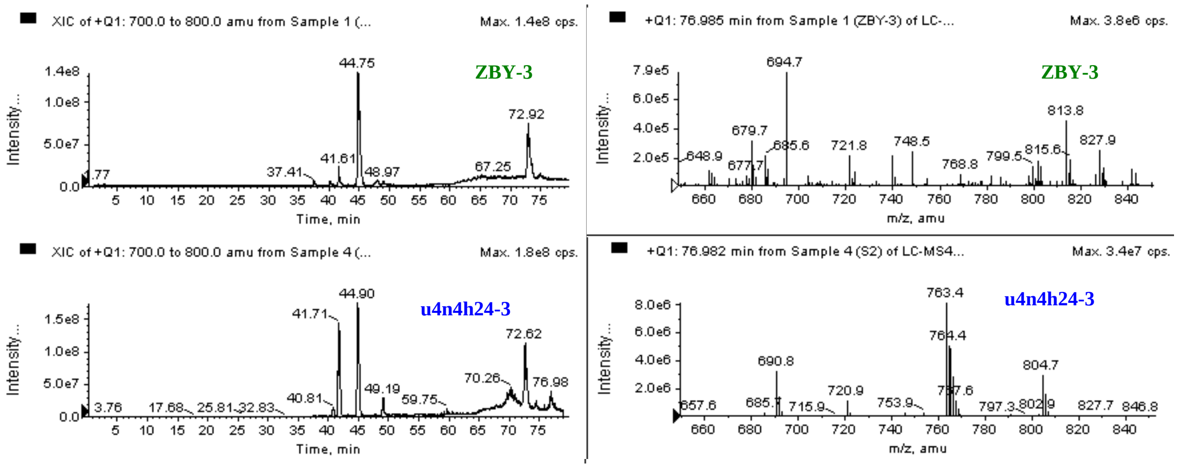

(F4)

Extracted Positive Ion (*m/z* 300.0-400.0) Chromatograms ESIMS Spectra taken at the 38.68 min Retention Time

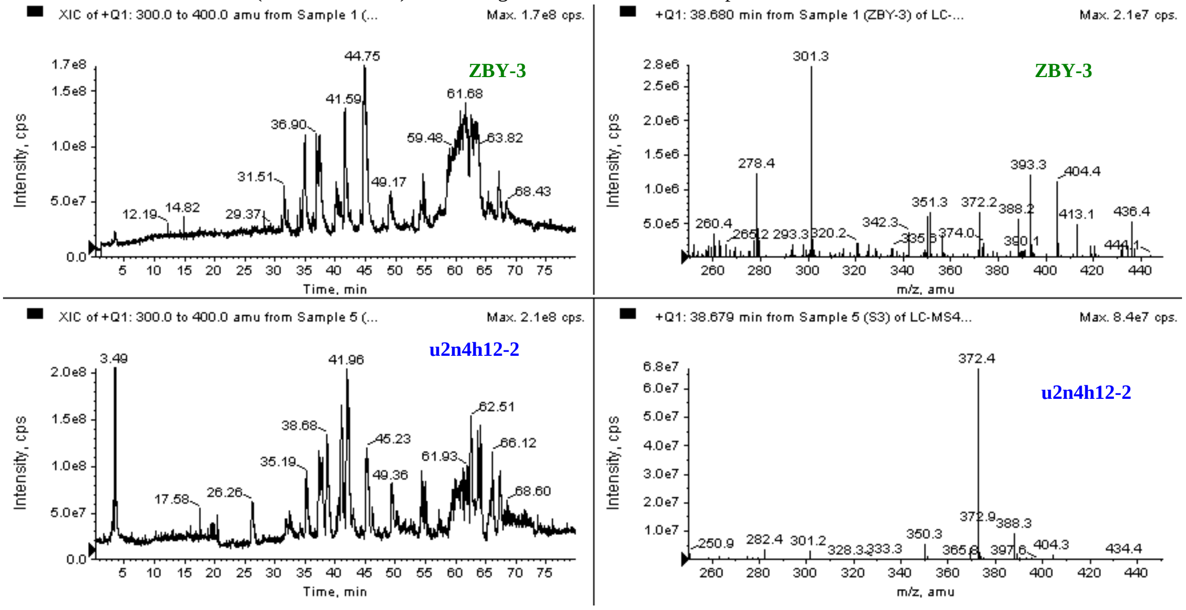

(F5)

Figure S2. *Cont.*

Extracted Positive Ion (*m/z* 500.0-600.0) Chromatograms ESIMS Spectra taken at the 69.33 min Retention Time

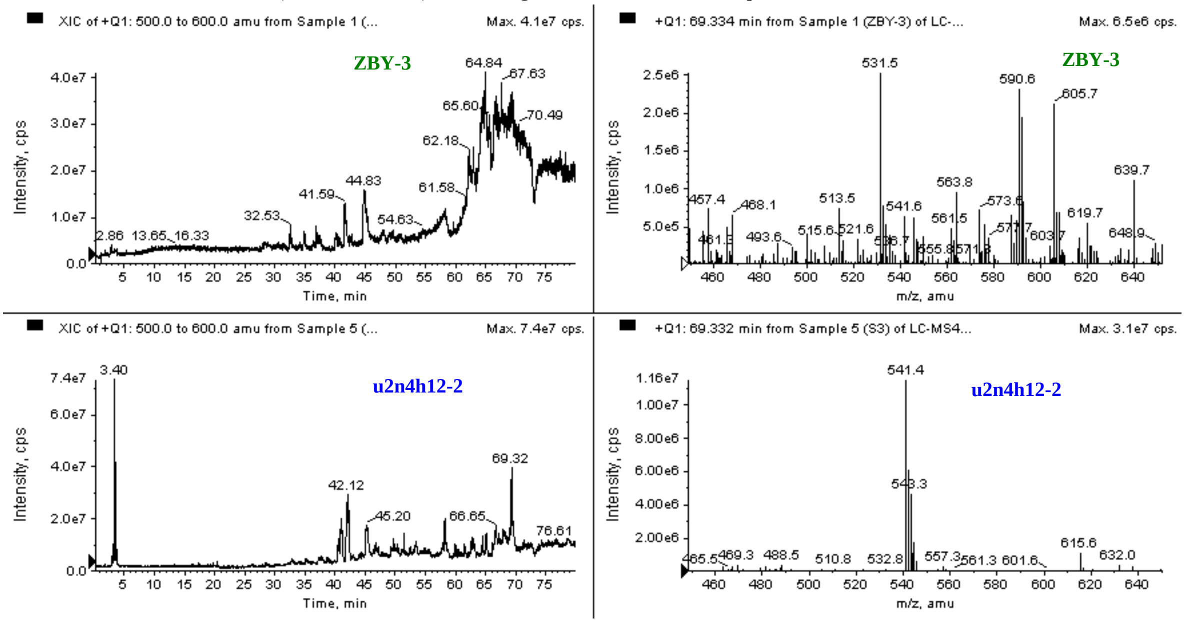

(F6)

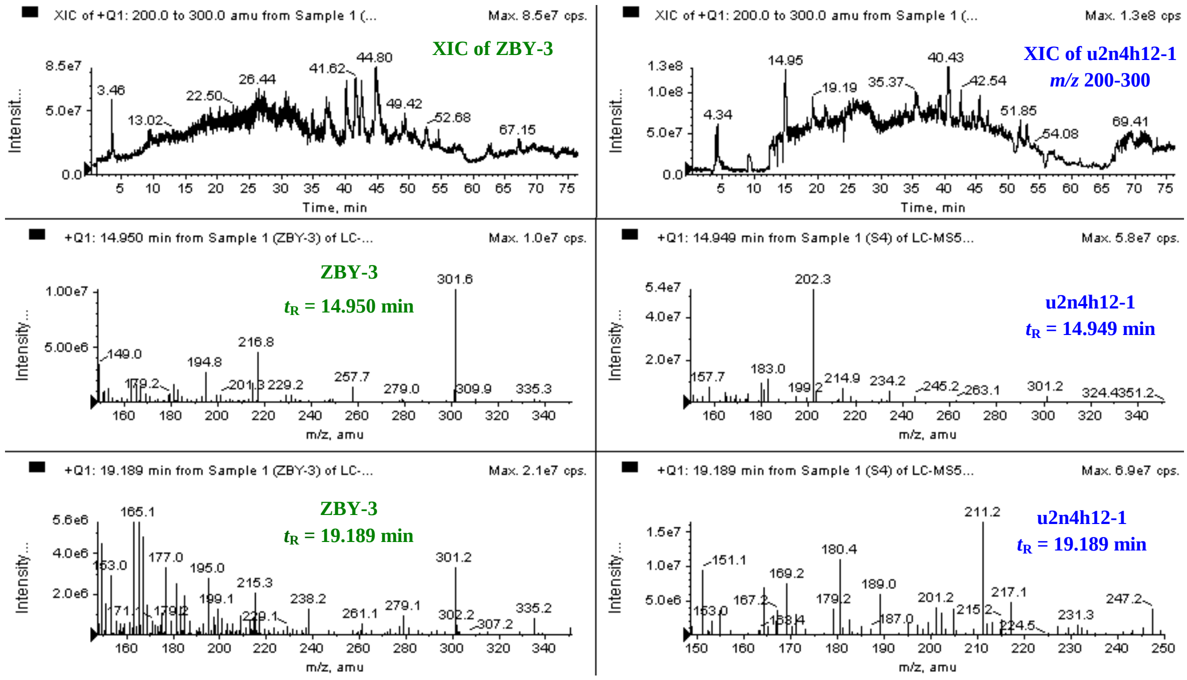

(F7)

Figure S2. *Cont.*

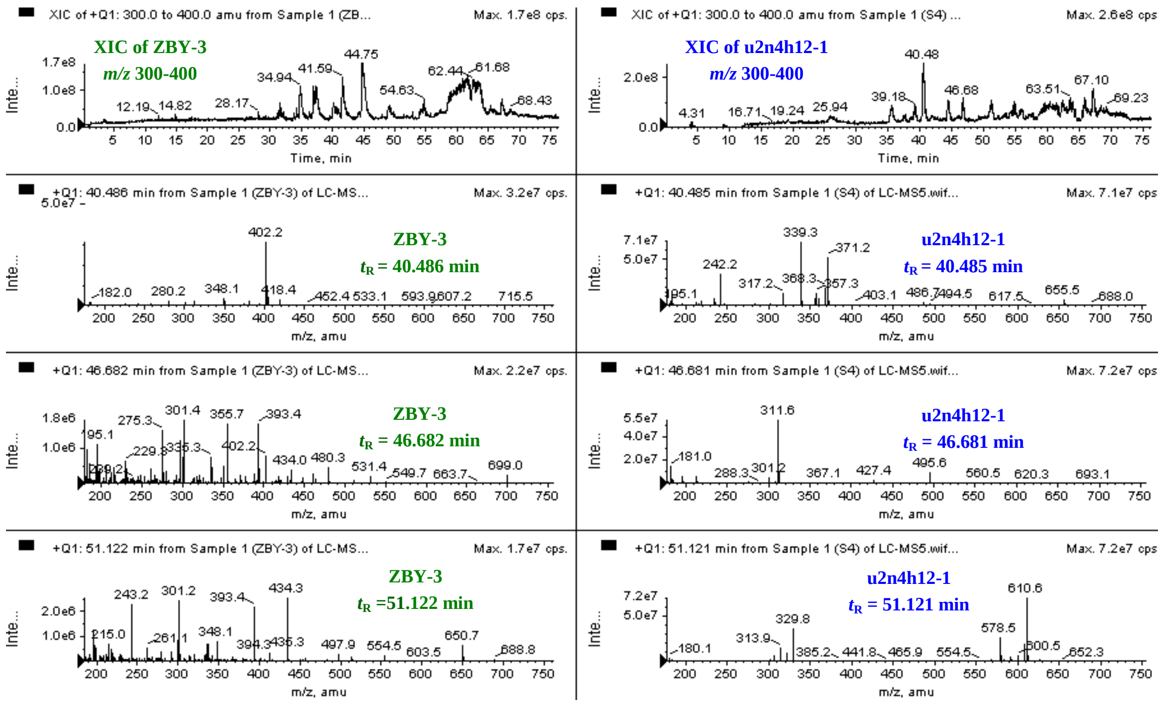

(F8)

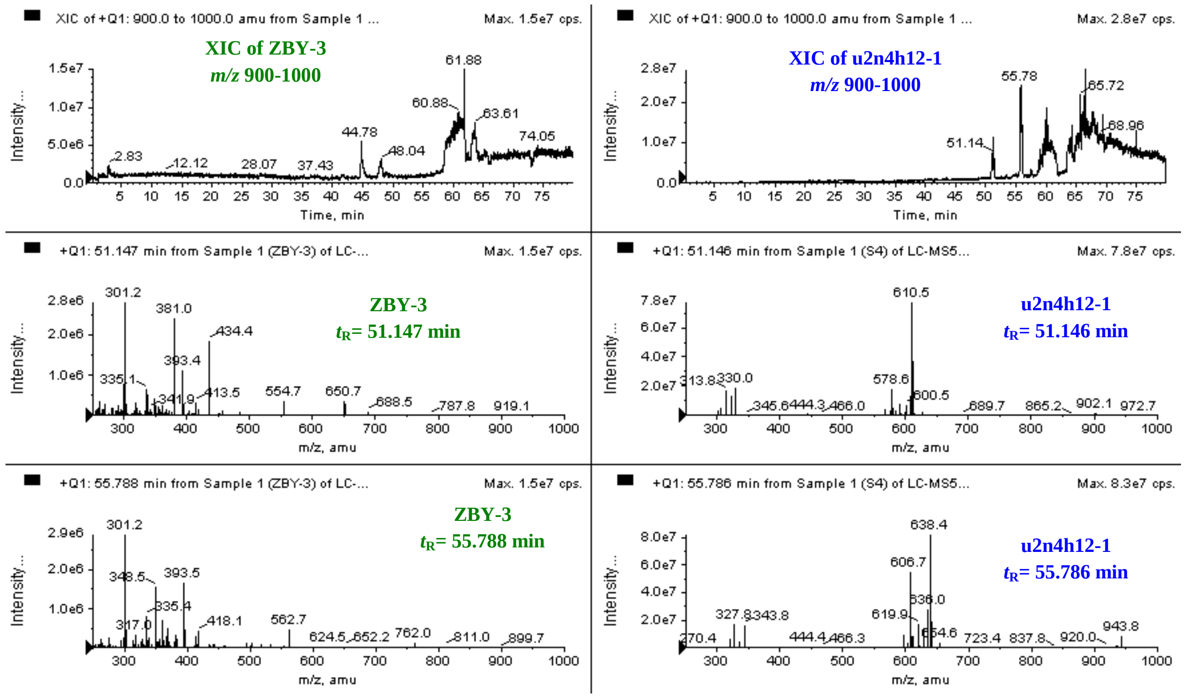

(F9)

Figure S2. *Cont.*

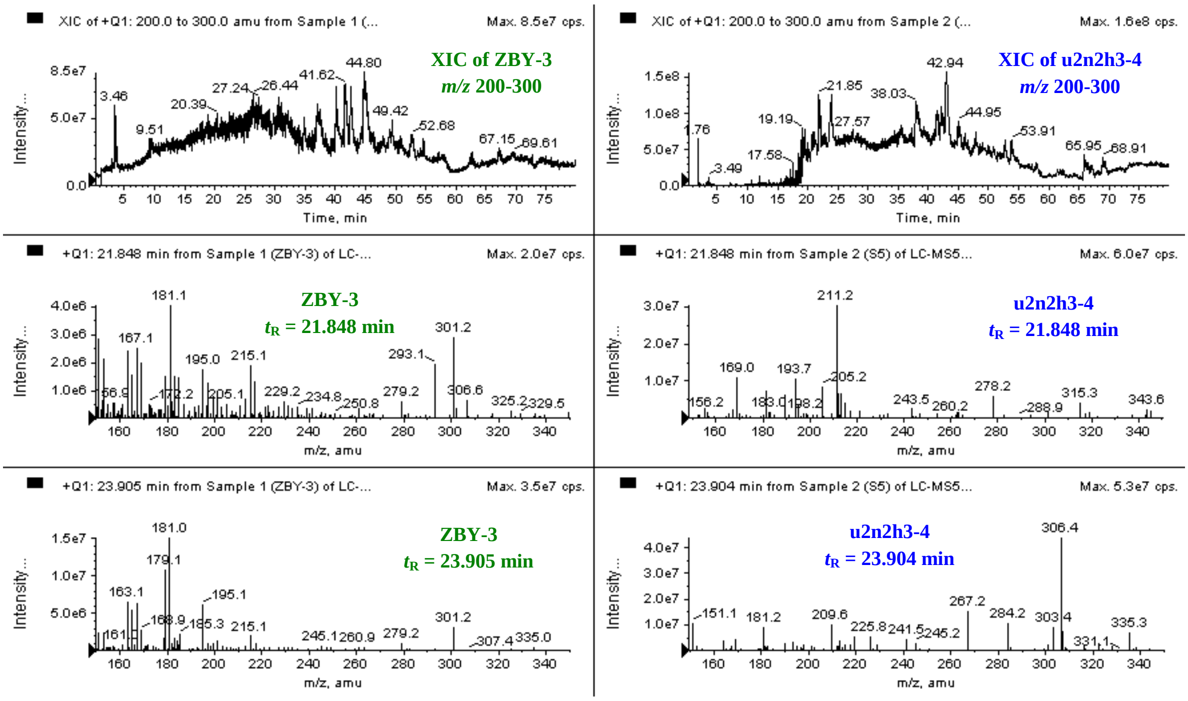

(F10)

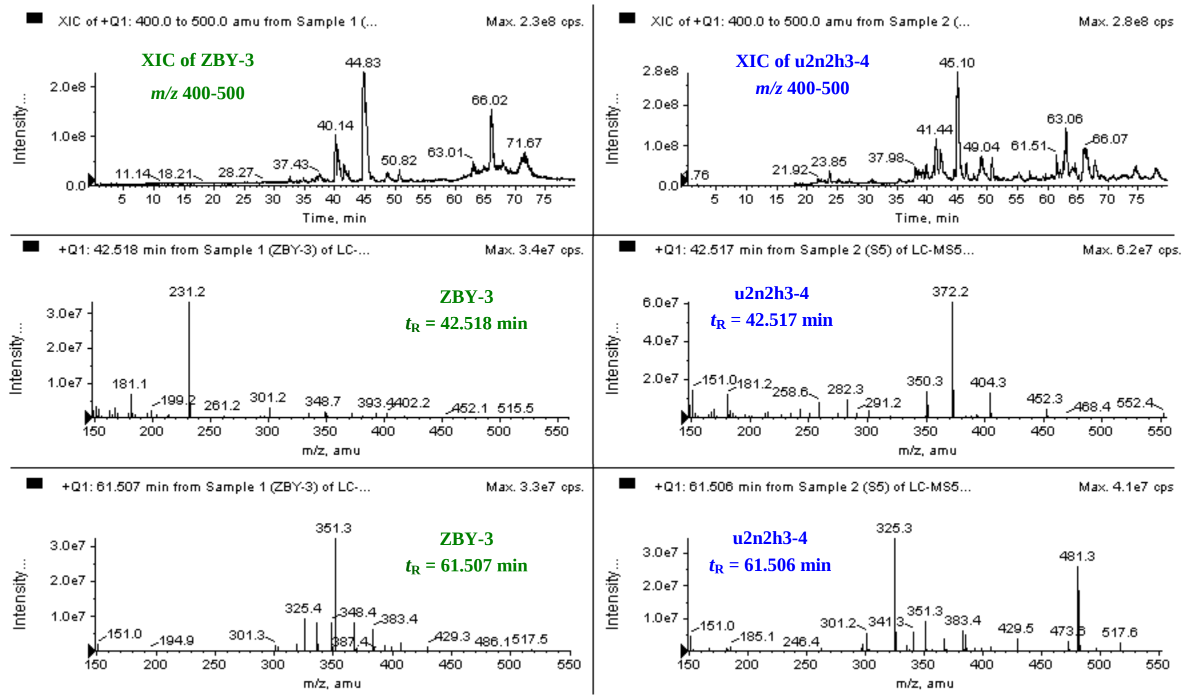

(F11)

Figure S2. *Cont.*

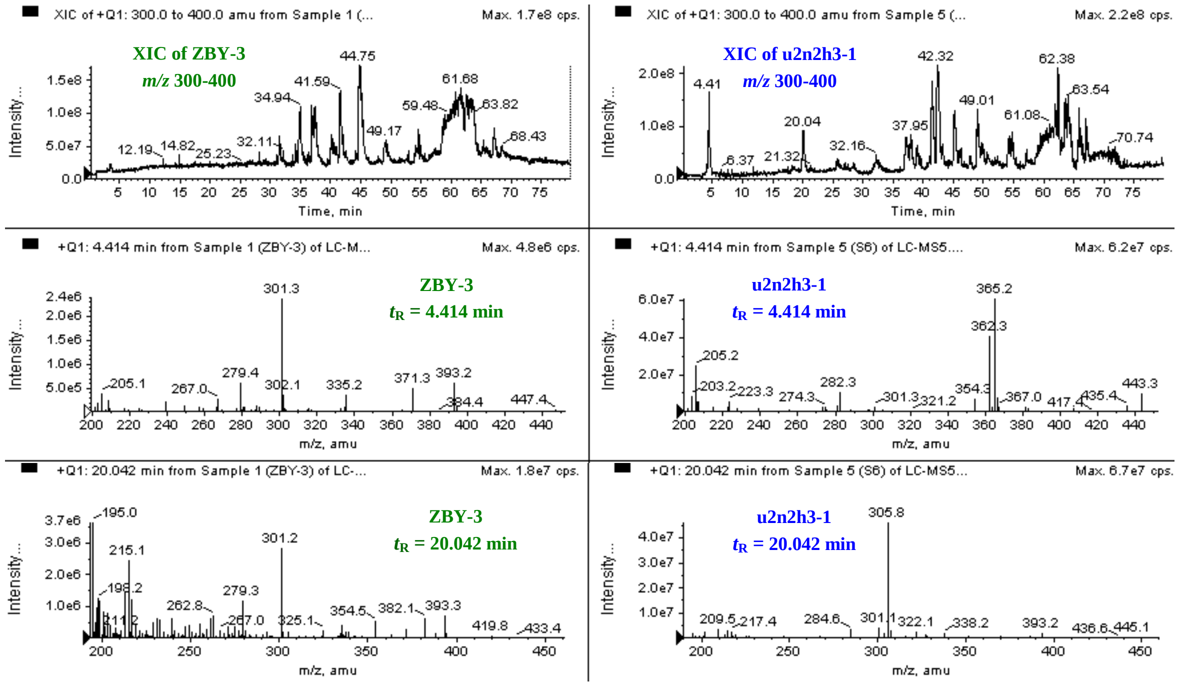

(F12)

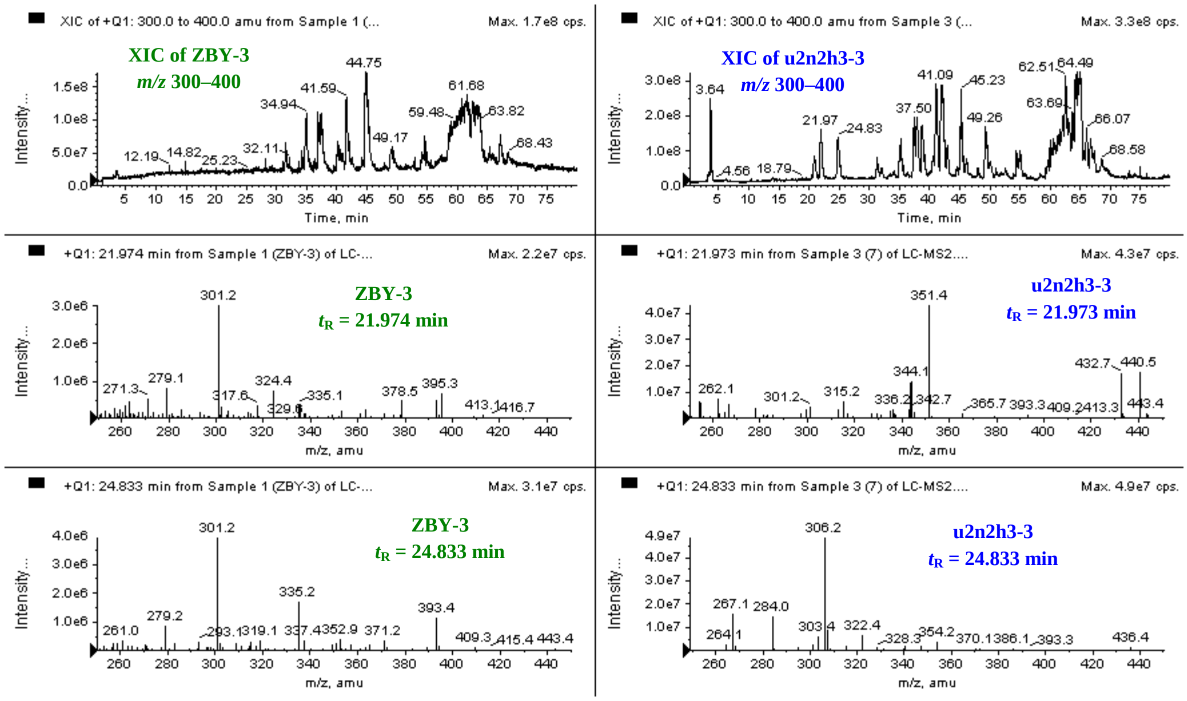

(F13)

Figure S2. *Cont.*

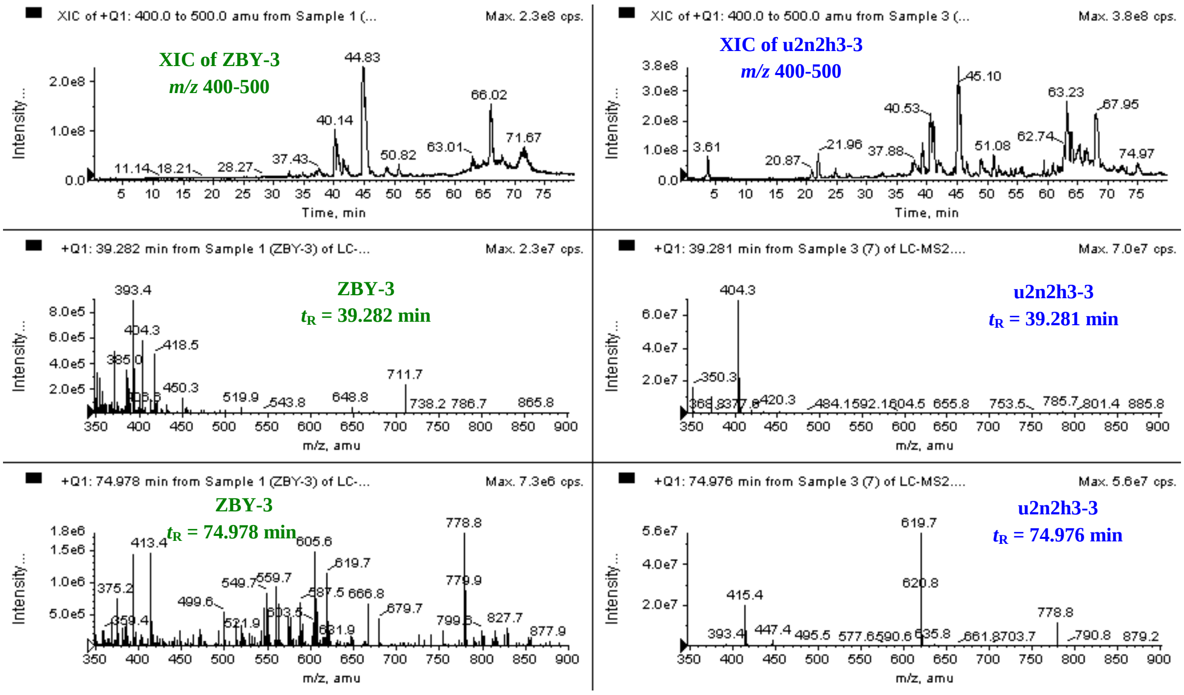

(F14)

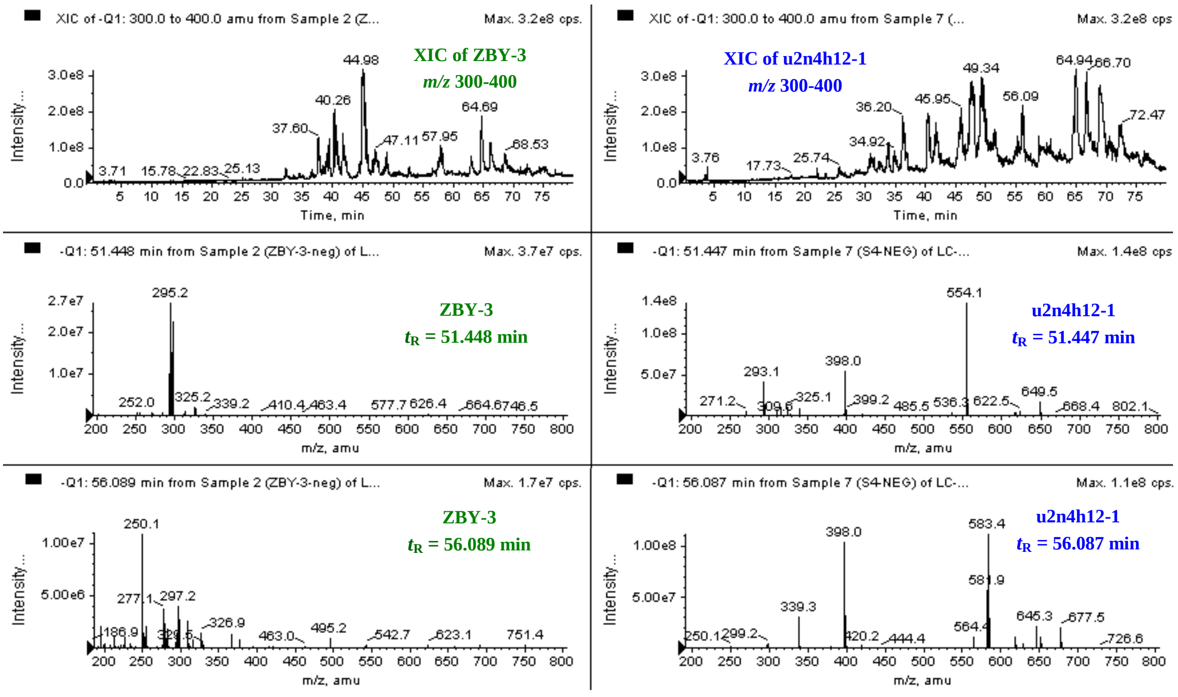

(F15)

Figure S2. *Cont.*

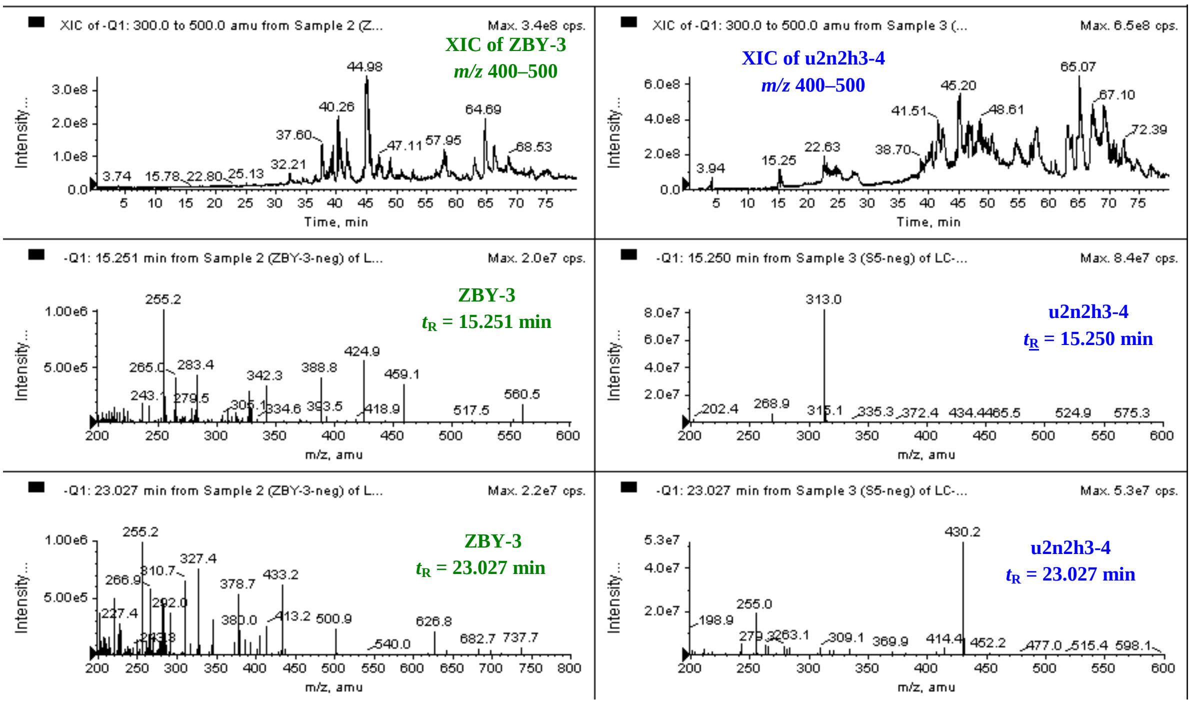

(**F16**)

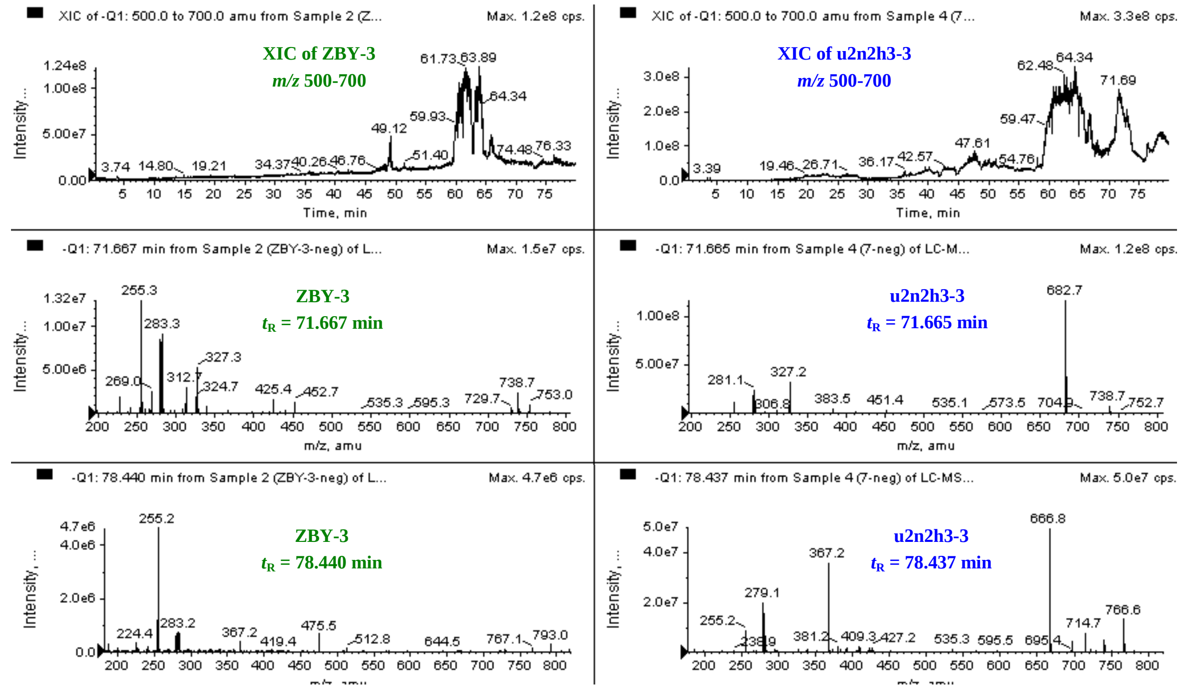

(**F17**)

Figure S3. HPLC-PDAD-UV analysis of **1**–**6** and the EtOAc extracts of the mutant u2n2h3-3 and the control ZBY-3 strain. (**A**) HPLC profiles detected at 210 nm; (**B**) HPLC profiles detected at 257 nm; (**C**) UV spectra for detecting **1**–**6** in the HPLC profiles of u2n2h3-3 and ZBY-3 extract.

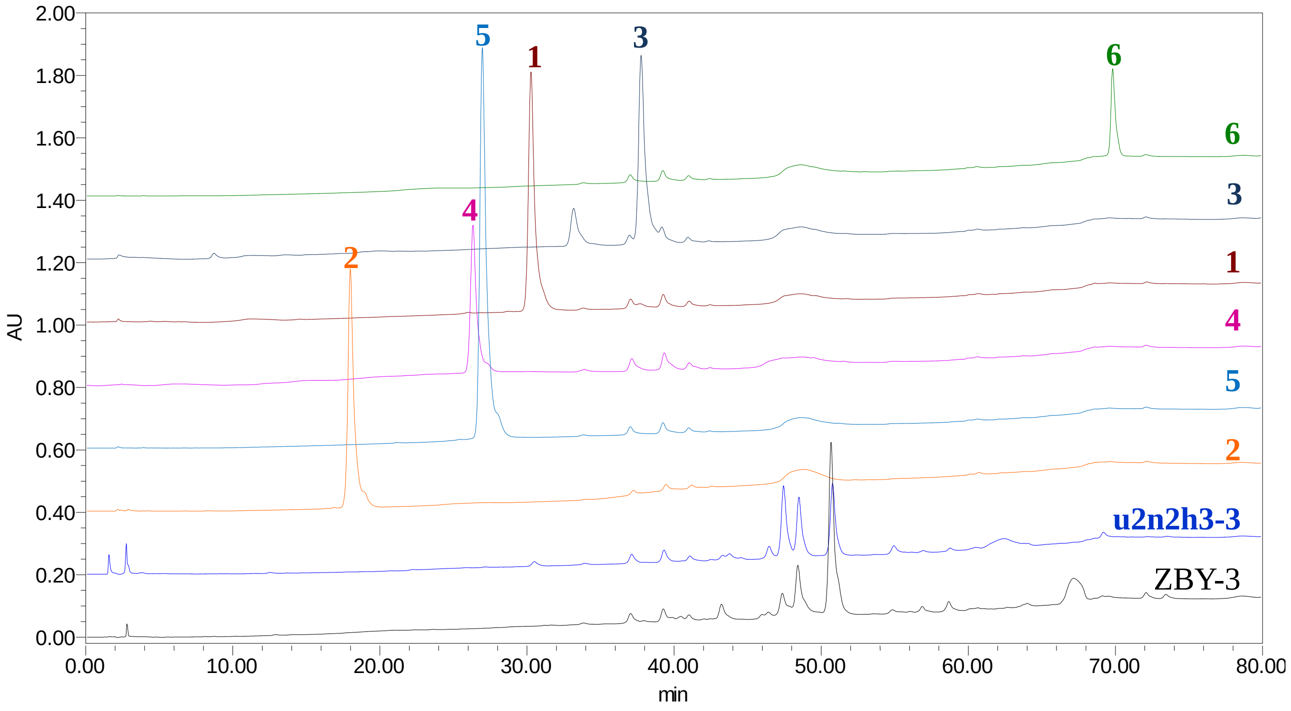

(**A**)

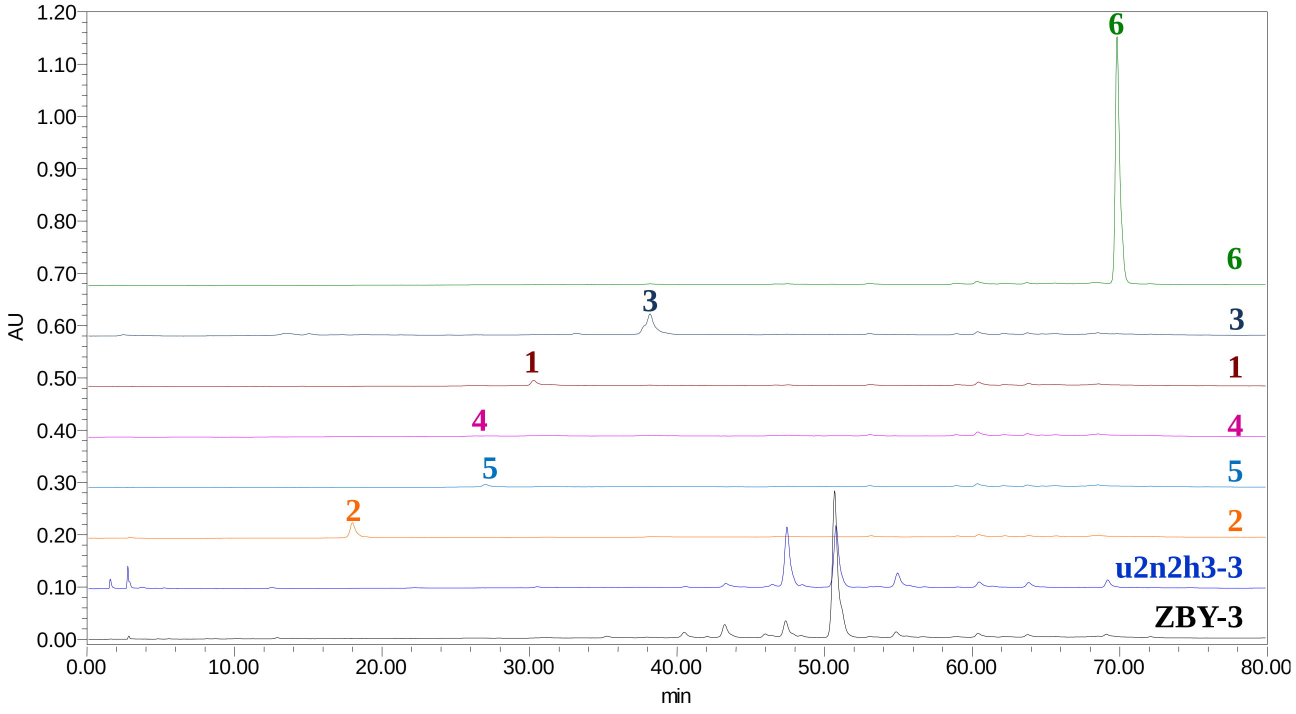

(**B**)

Figure S3. *Cont.*

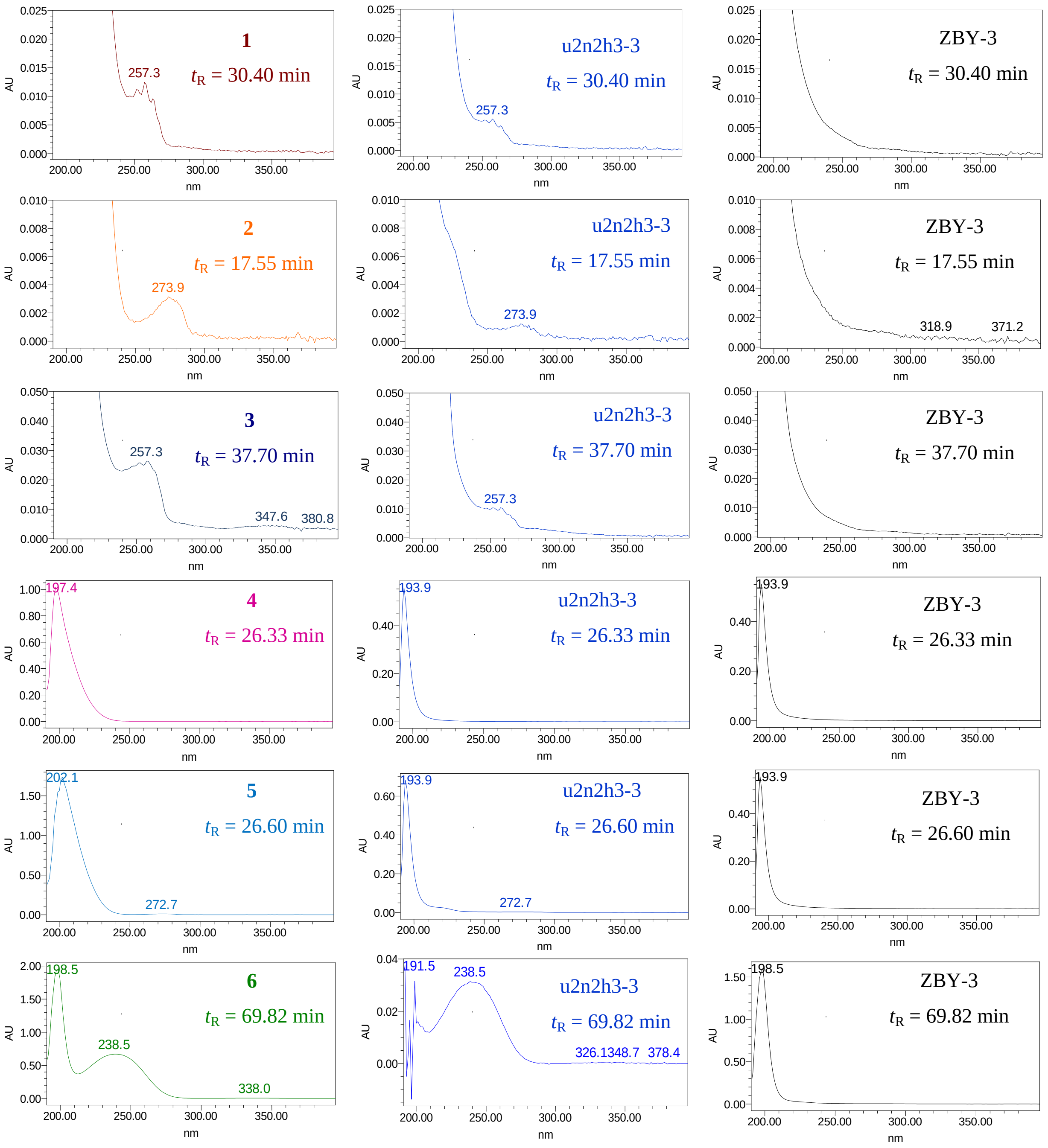

(C)

Figure S4. HPLC-ESI-MS analysis of **1**–**6** and the EtOAc extracts of the mutant u2n2h3-3 and the control ZBY-3 strain. (**A**) HPLC-positive ion ESI-MS analysis (ESIMS *m*/*z*: 267 $[M + Na]^+$ for **1**); (**B**) HPLC-negative ion ESI-MS analysis (ESIMS *m*/*z*: 243 $[M - H]^-$ for **1**); (**C**) HPLC-positive ion ESI-MS analysis (ESIMS *m*/*z*: 283 $[M + Na]^+$ for **2**); (**D**) HPLC-negative ion ESI-MS analysis (ESIMS *m*/*z*: 259 $[M - H]^-$ for **2**); (**E**) HPLC-positive ion ESI-MS analysis (ESIMS *m*/*z*: 256 $[M + Na]^+$ for **3**); (**F**) HPLC-positive ion ESI-MS analysis (ESIMS *m*/*z*: 233 $[M + Na]^+$ for **4**); (**G**) HPLC-positive ion ESI-MS analysis (ESIMS *m*/*z*: 233 $[M + Na]^+$ for **5**); (**H**) HPLC-negative ion ESI-MS analysis (ESIMS *m*/*z*: 209 $[M - H]^-$ for **5**); (**I**) HPLC-positive ion ESI-MS analysis (ESIMS *m*/*z*: 467 $[M + Na]^+$ for **6**); (**J**) HPLC-negative ion ESI-MS analysis (ESIMS *m*/*z*: 443 $[M - H]^-$ for **6**).

Extracted Positive Ion (*m/z* 267.0–268.0) Chromatograms

ESIMS Spectra in the Range of 24.0–26.5 min Retention Times

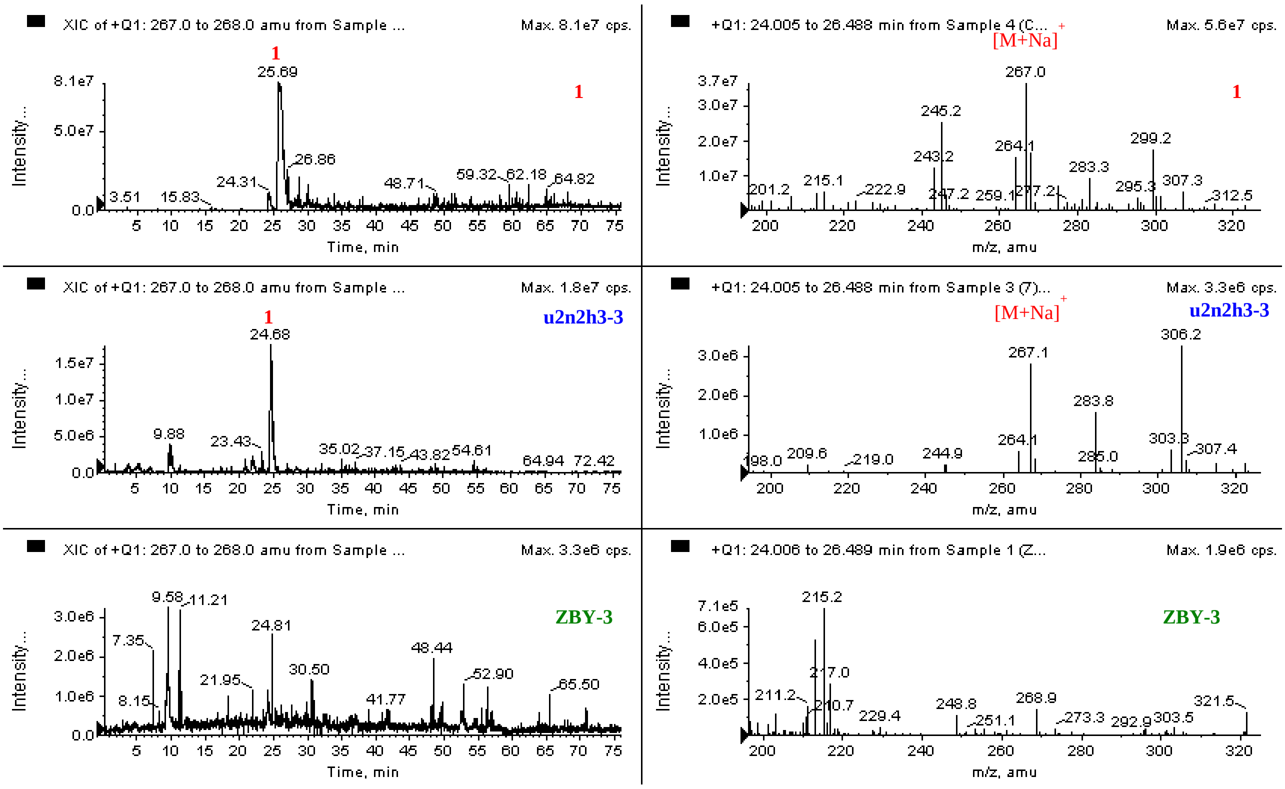

(A)

Figure S4. *Cont.*

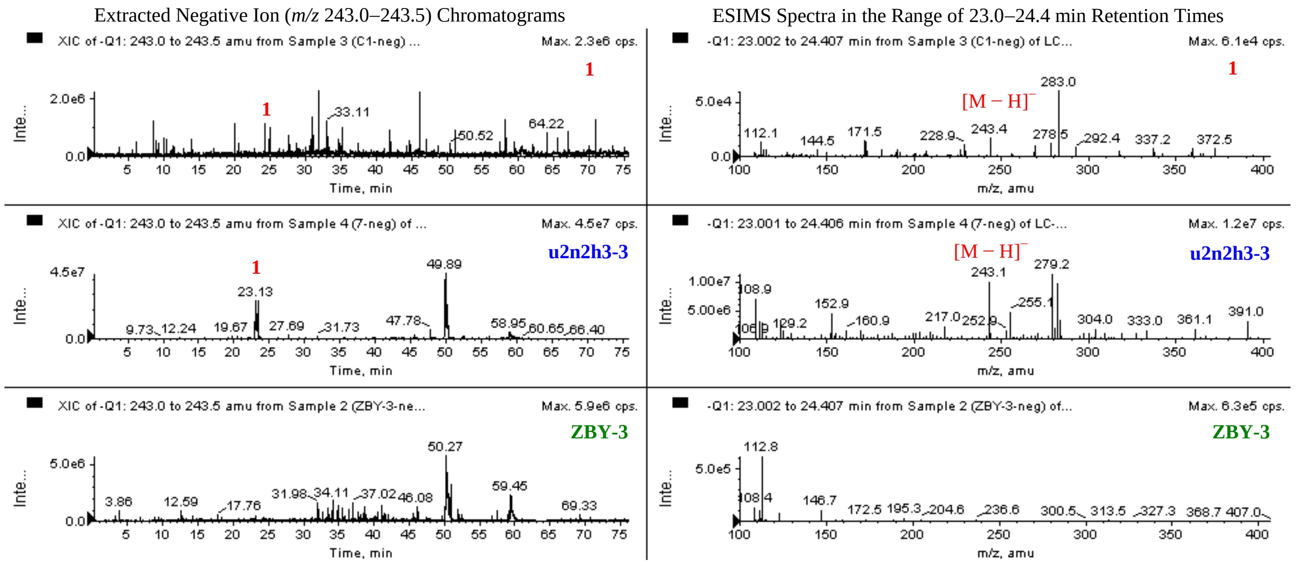

(B)

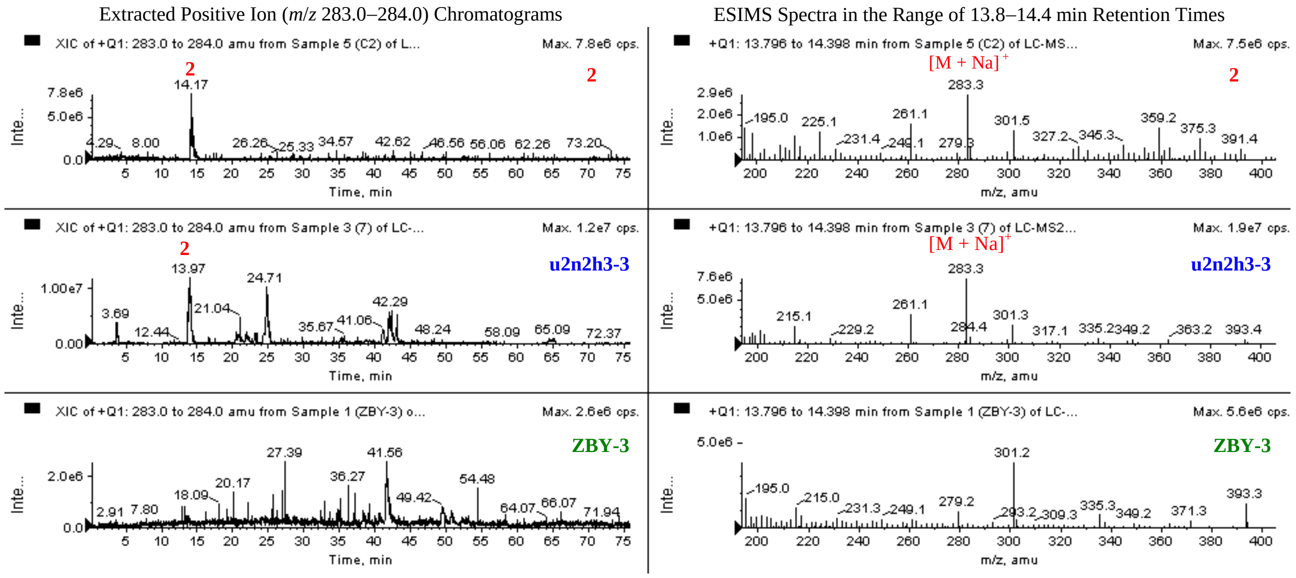

(C)

Figure S4. *Cont.*

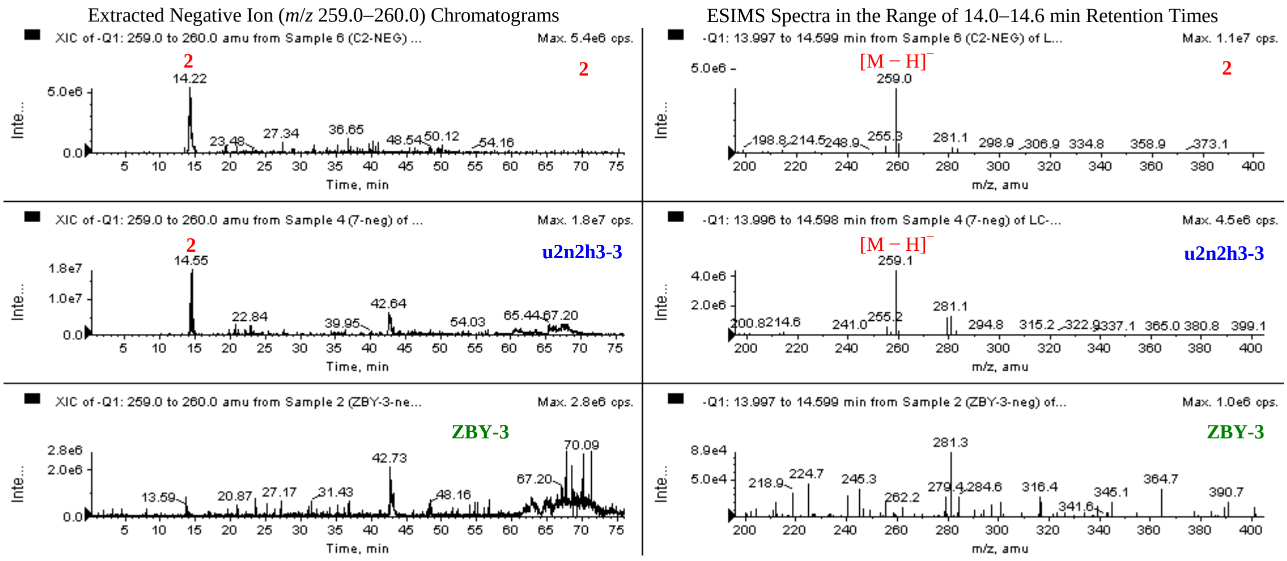

(**D**)

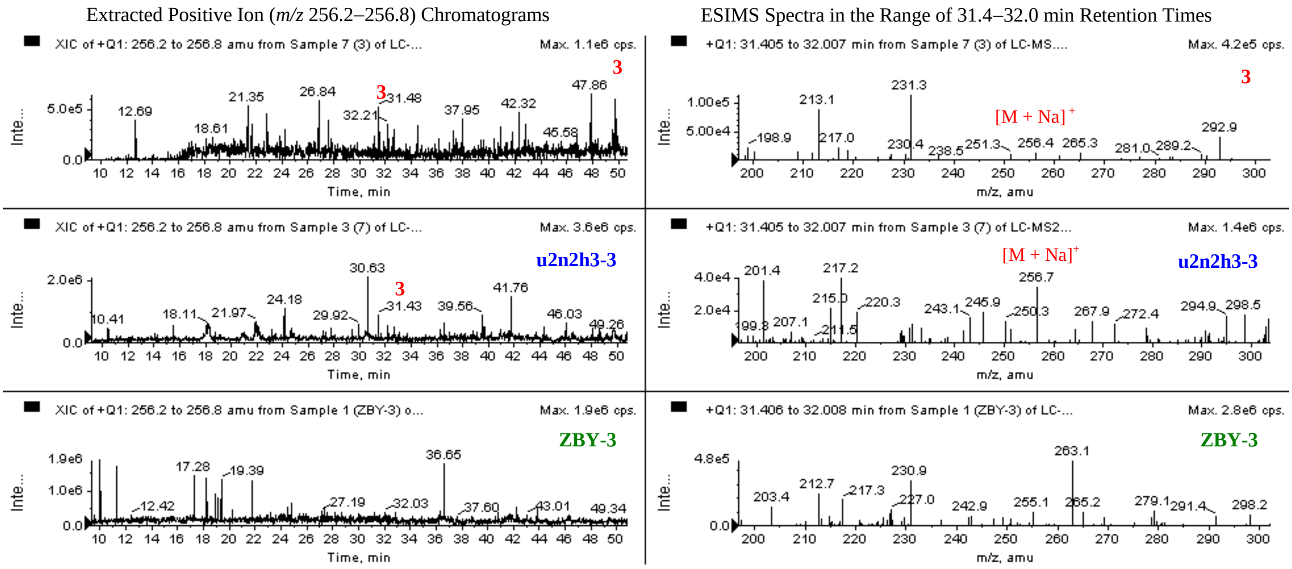

(**E**)

Figure S4. *Cont.*

Extracted Positive Ion (*m/z* 233.0–233.4) Chromatograms ESIMS Spectra in the Range of 20.6–22.0 min Retention Times

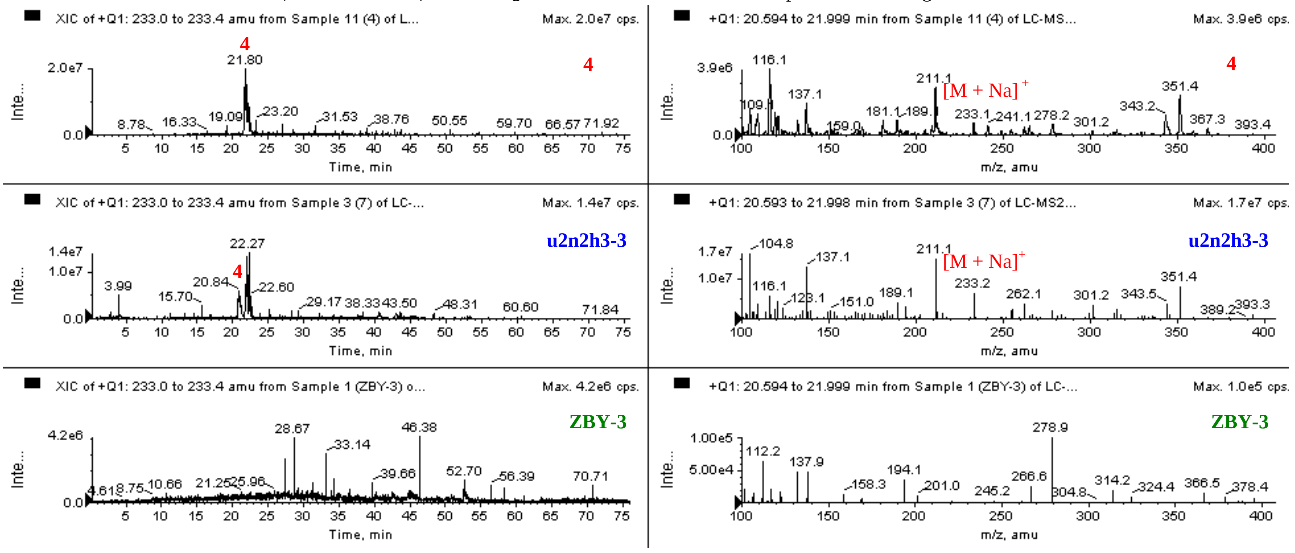

(F)

Extracted Positive Ion (*m/z* 233.0–233.4) Chromatograms ESIMS Spectra in the Range of 22.0–23.0 min Retention Times

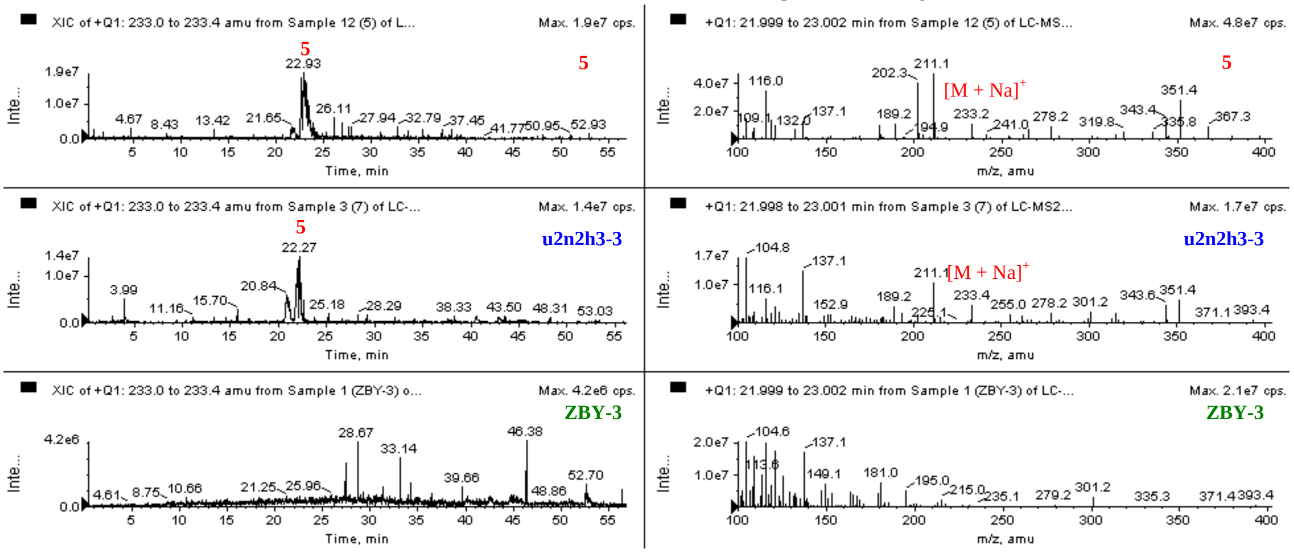

(G)

Figure S4. *Cont.*

Extracted Negative Ion (*m/z* 209.0–210.0) Chromatograms ESIMS Spectra in the Range of 21.0–23.0 min Retention Times

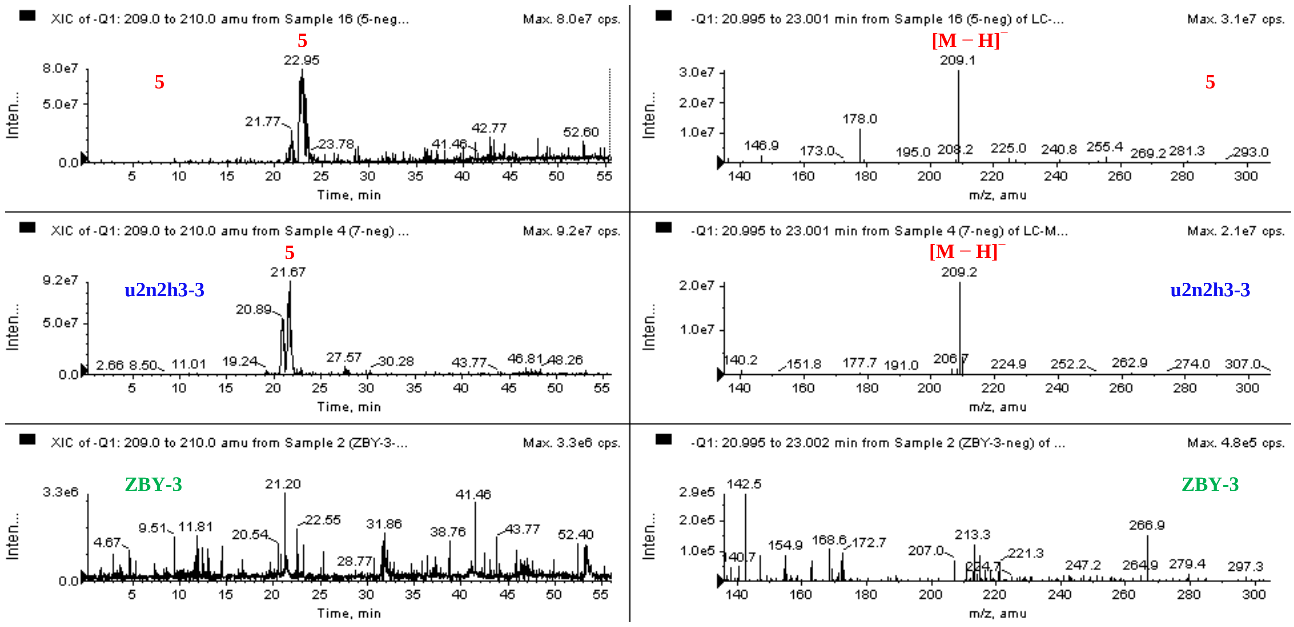

(H)

Extracted Positive Ion (*m/z* 467.0–468.0) Chromatograms ESIMS Spectra in the Range of 63.6–64.4 min Retention Times

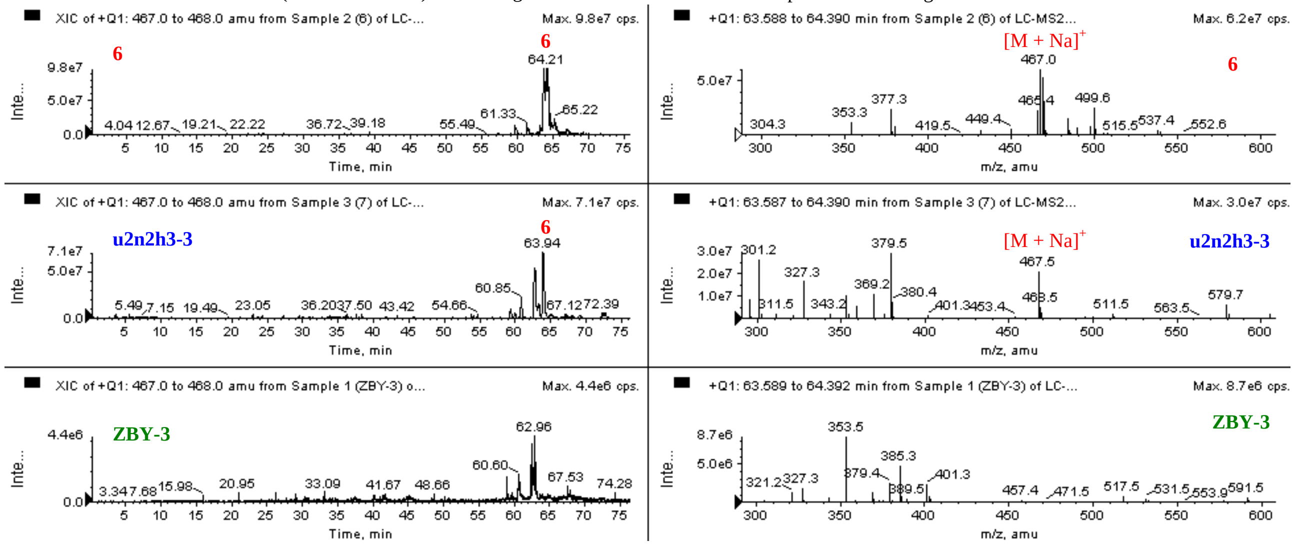

(I)

Figure S4. *Cont.*

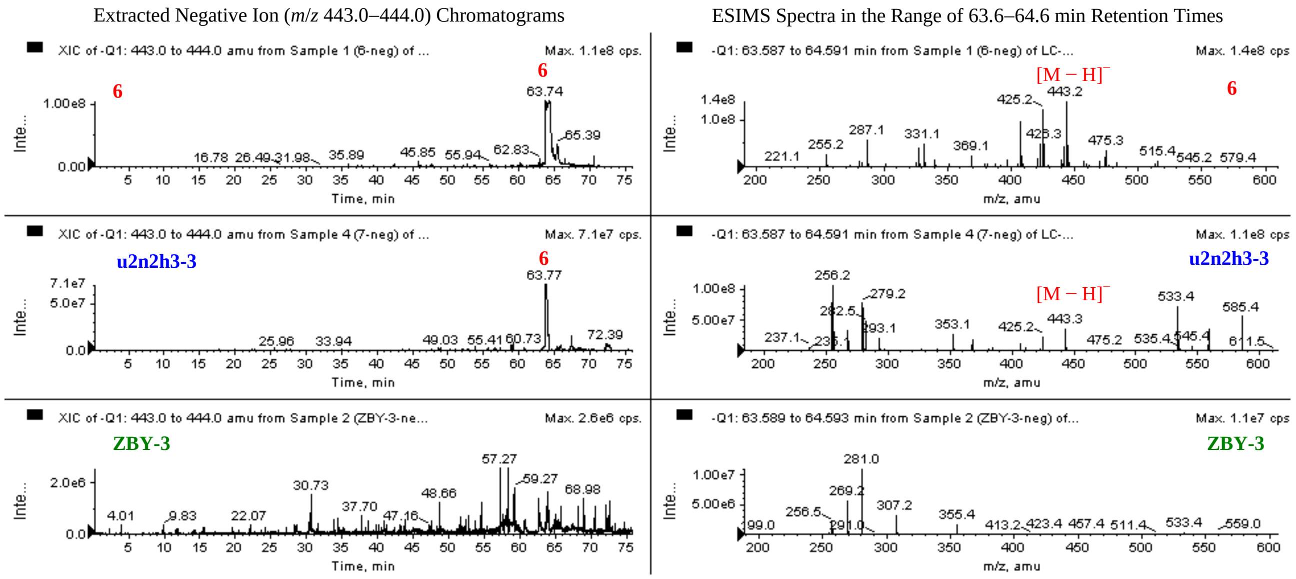

(J)

Figure S5. Positive ESI-MS and HR-ESI-MS of **3**.

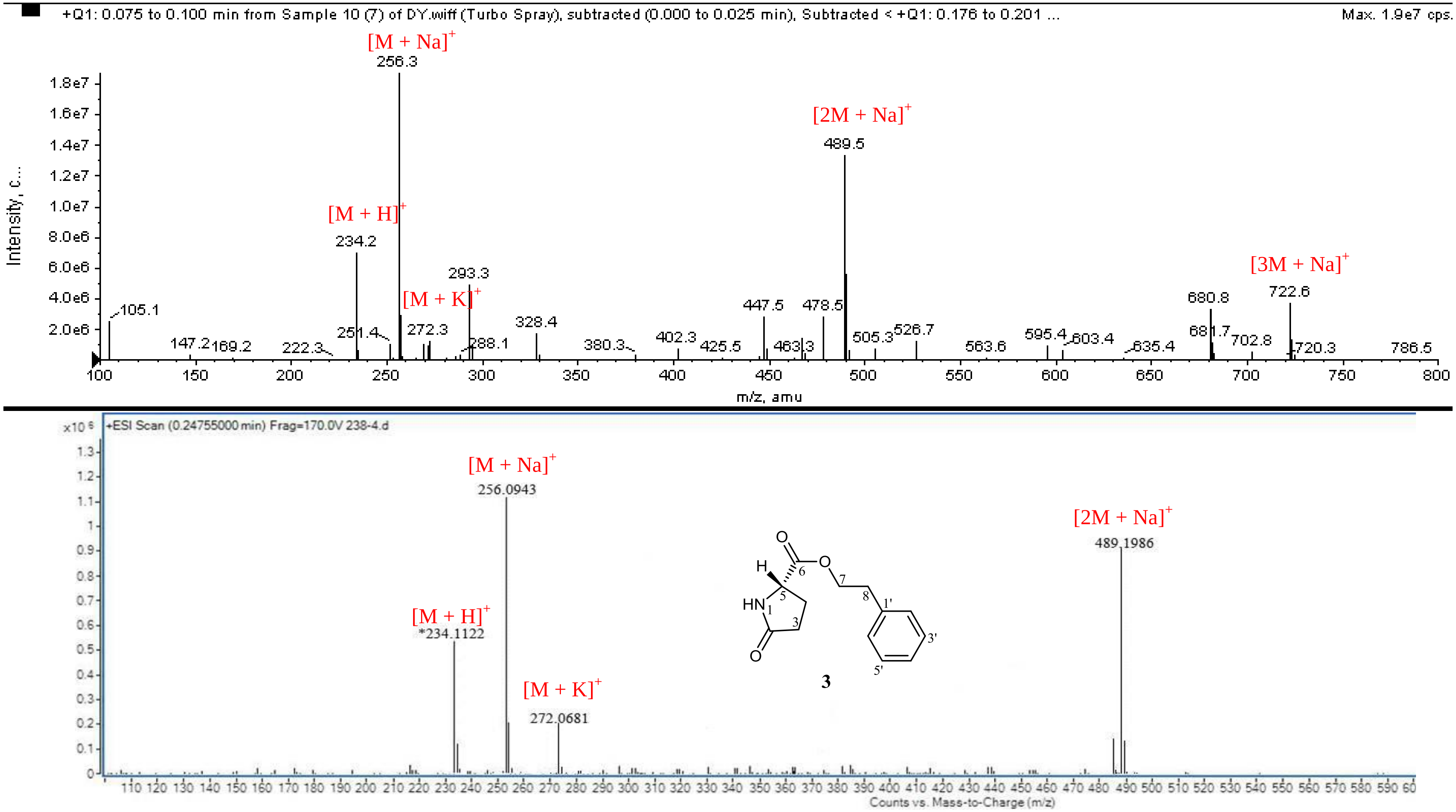

Figure S6. 400 MHz ^{1}H NMR of **3** in CD_3OD.

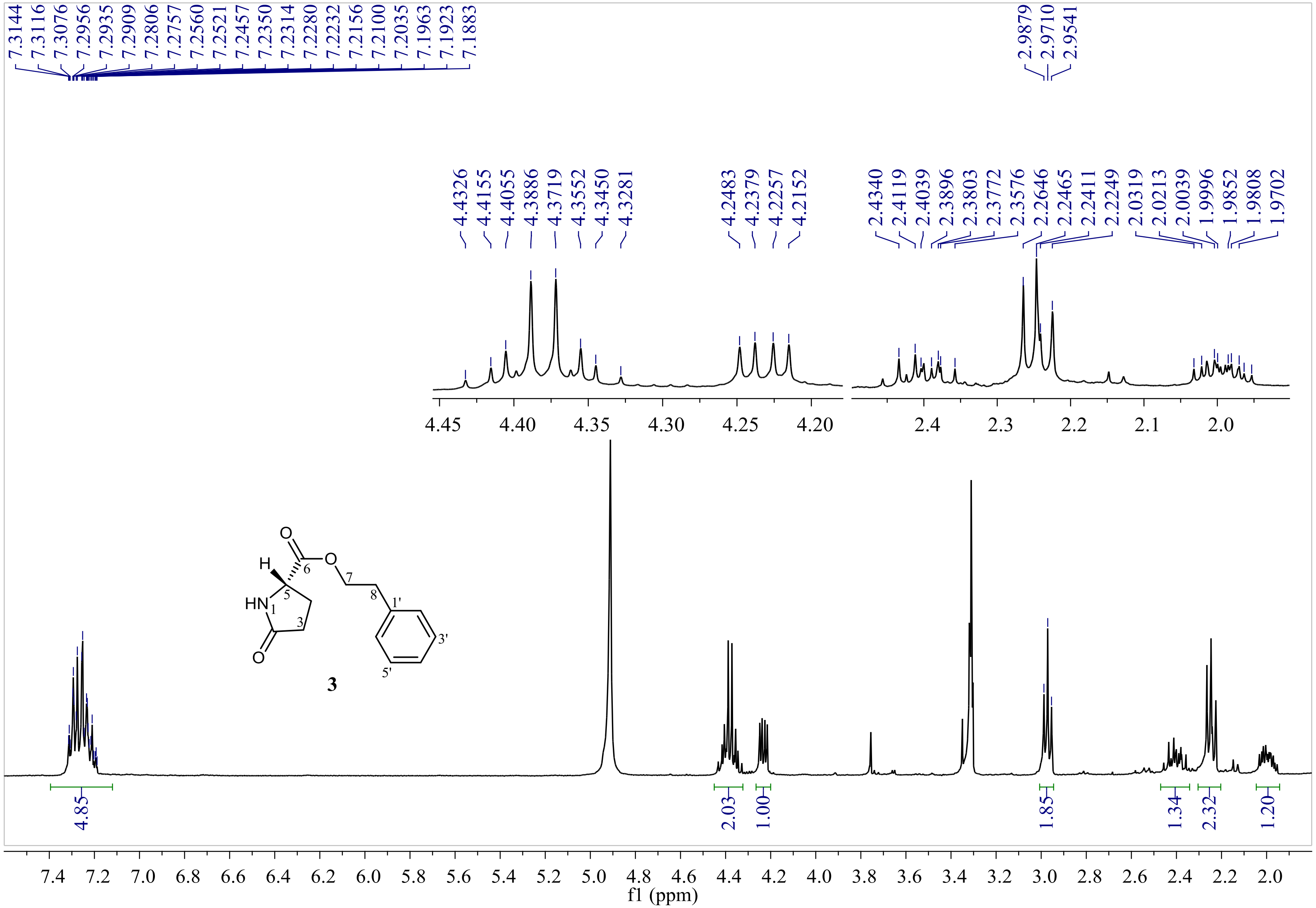

Figure S7. 100 MHz ^{13}C NMR of **3** in CD_3OD.

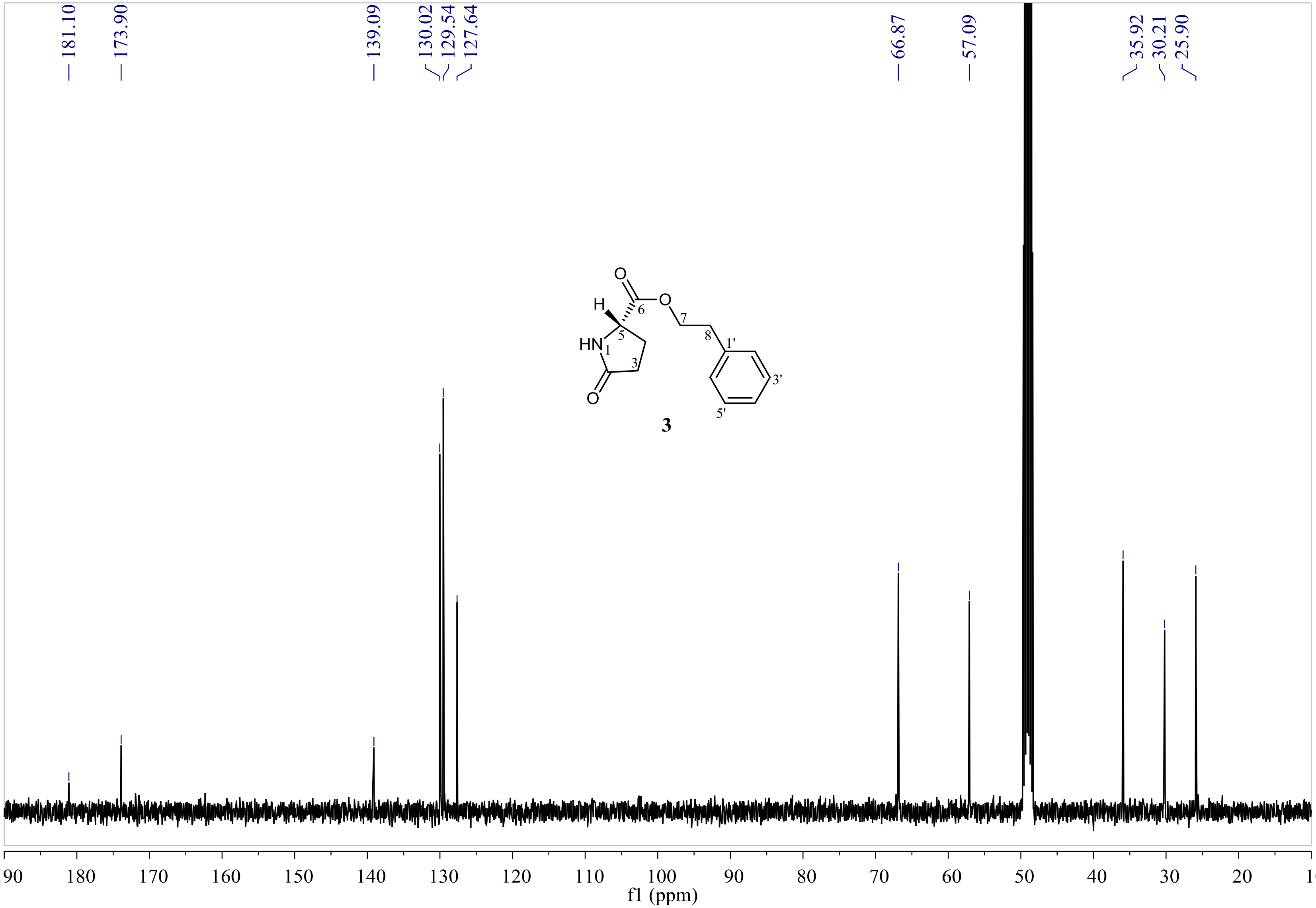

Figure S8. ^{1}H-^{1}H COSY spectrum of **3** in CD_3OD.

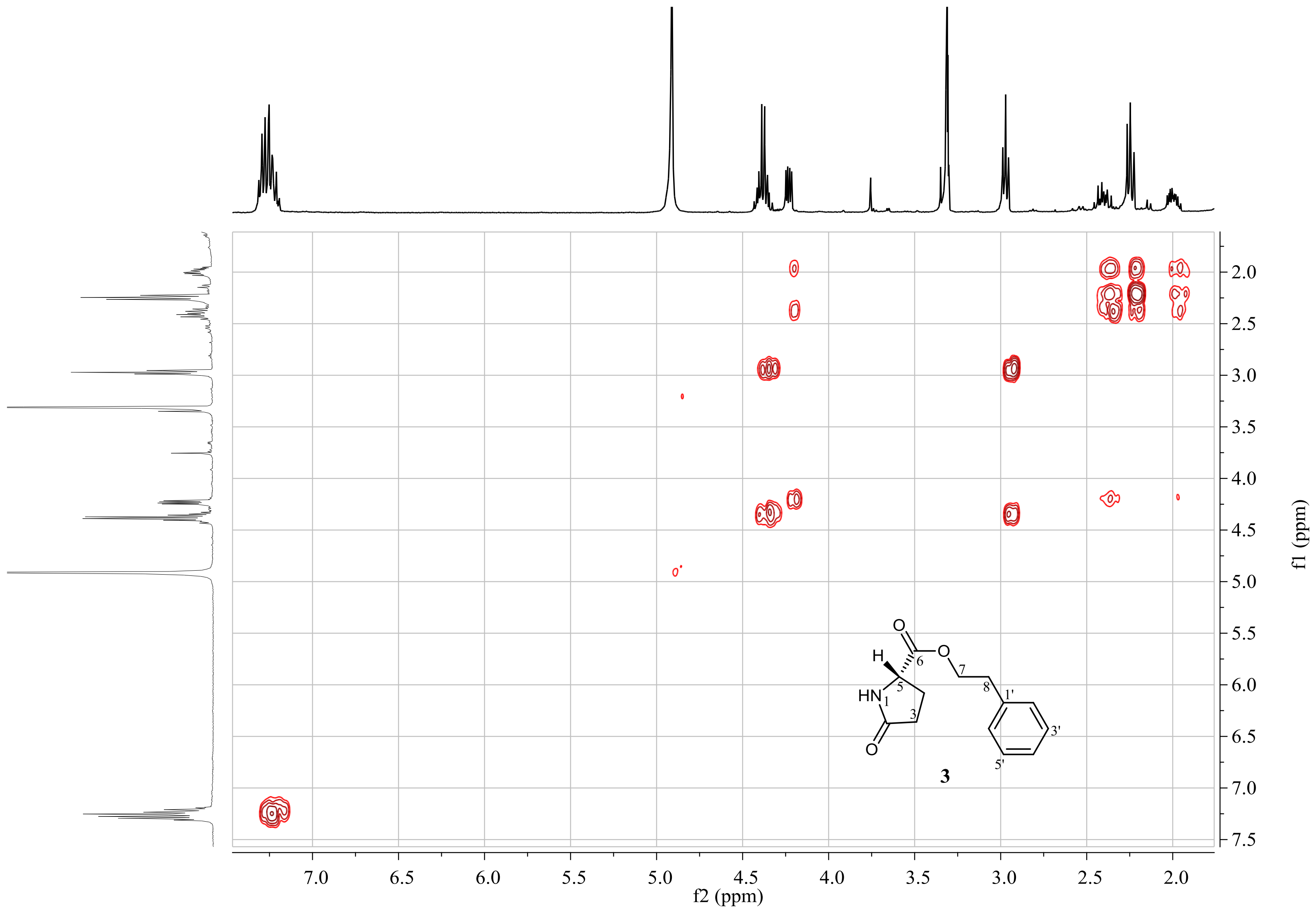

Figure S9. HMQC spectrum of **3** in CD_3OD.

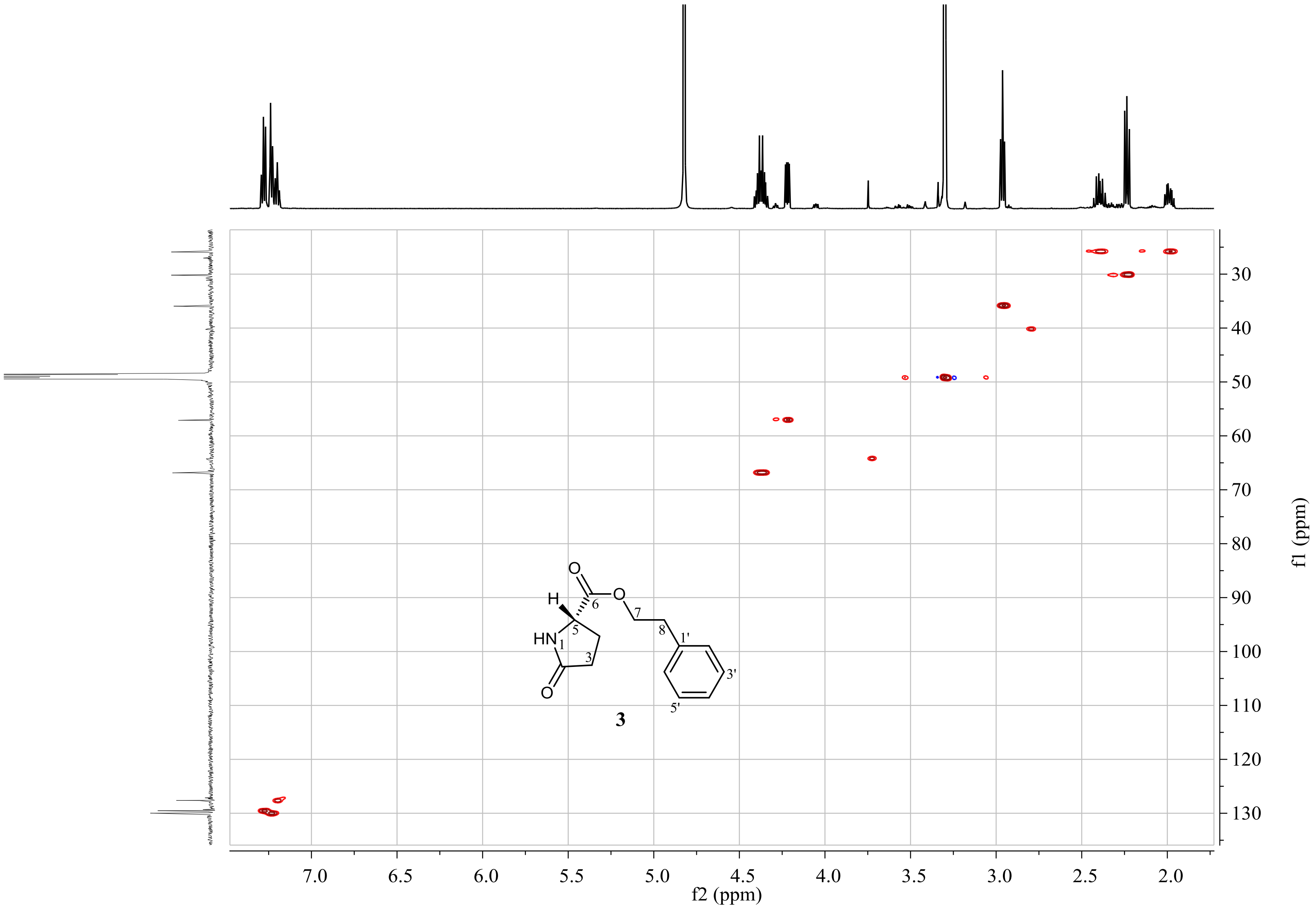

Figure S10. HMBC spectrum of **3** in CD_3OD.

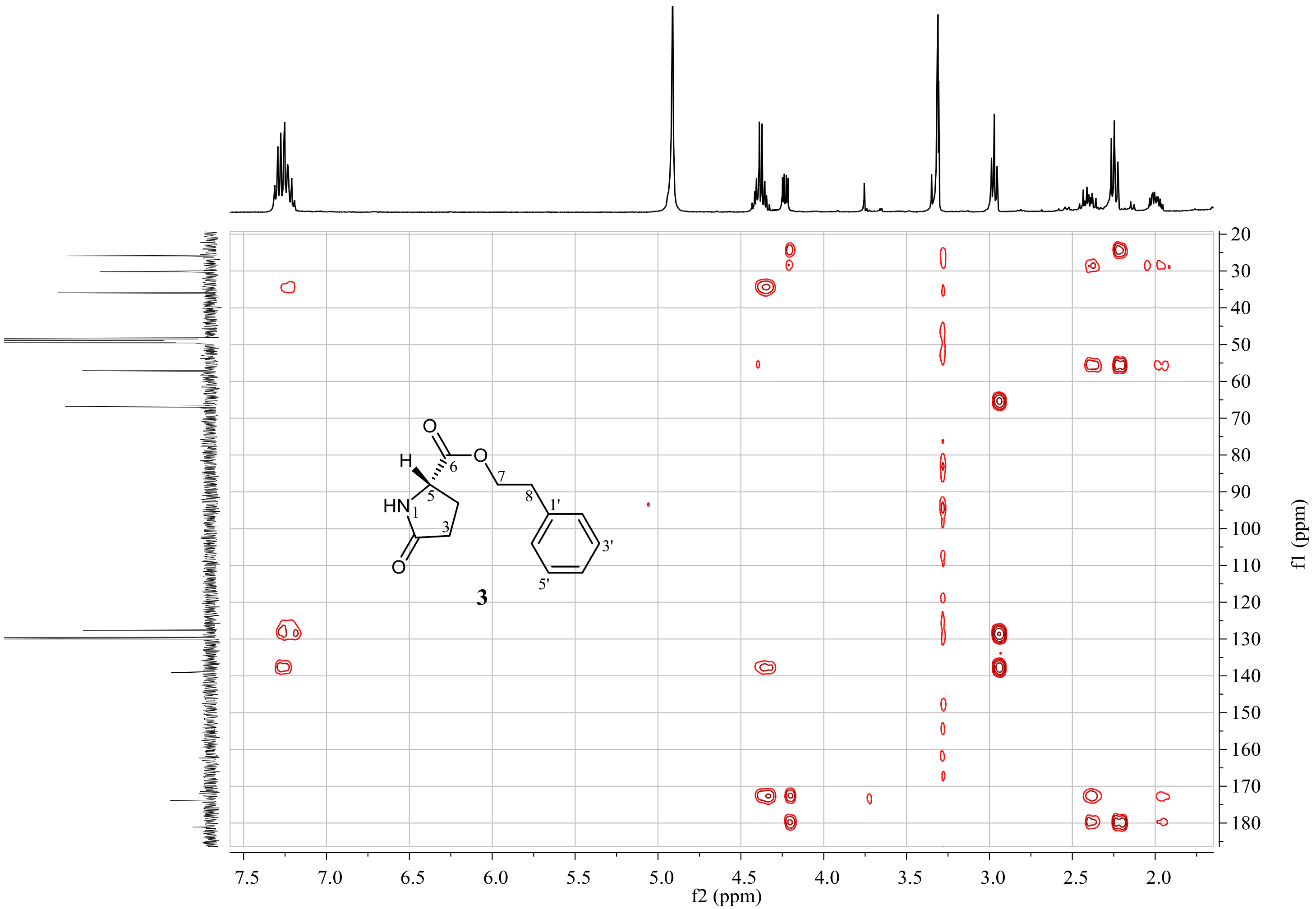

© 2014 by the authors; licensee MDPI, Basel, Switzerland. This article is an open access article distributed under the terms and conditions of the Creative Commons Attribution license (http://creativecommons.org/licenses/by/3.0/).
